# Exploration of the Interaction Strength at the Interface of Anionic Chalcogen Anchors and Gold (111)-Based Nanomaterials

**DOI:** 10.3390/nano10061237

**Published:** 2020-06-25

**Authors:** Sebastián Miranda-Rojas, Fernando Mendizabal

**Affiliations:** 1 Departamento de Ciencias Químicas, Facultad de Ciencias Exactas, Universidad Andres Bello, Av. República 275, Santiago PO 8370146, Chile; 2 Departamento de Química, Facultad de Ciencias, Universidad de Chile, Las Palmeras 3425, Ñuñoa, Santiago PO 7800003, Chile

**Keywords:** supramolecular chemistry, noncovalent Interaction, gold, chalcogenides

## Abstract

Nowadays, the use of sulfur-based ligands to modify gold-based materials has become a common trend. Here, we present a theoretical exploration of the modulation of the chalcogenides-gold interaction strength, using sulfur, selenium, and tellurium as anchor atoms. To characterize the chalcogenide-gold interaction, we designed a nanocluster of 42 gold atoms (Au_42_) to model a gold surface (111) and a series of 60 functionalized phenyl-chalcogenolate ligands to determine the ability of electron-donor and -withdrawing groups to modulate the interaction. The analysis of the interaction was performed by using energy decomposition analysis (EDA), non-covalent interactions index (NCI), and natural population analysis (NPA) to describe the charge transfer processes and to determine data correlation analyses. The results revealed that the magnitudes of the interaction energies increase following the order S < Se < Te, where this interaction strength can be augmented by electron-donor groups, under the donor-acceptor character the chalcogen–gold interaction. We also found that the functionalization in *meta* position leads to better control of the interaction strength than the *ortho* substitution due to the steric and inductive effects involved when functionalized in this position.

## 1. Introduction

During the last decade, it has been evidenced a significant increase in the interest of exploring the possibility of adapting and modifying surface properties of metal substrates with applications in several areas of modern technology. Among these, some of the most common are the modifications in the ability of adhesion and friction [1,2,3] for protection [4], corrosion inhibition [5], catalysis [6], wettability [7], and nanotechnology [8]. An appropriate strategy to modify the surface properties is to prepare self-assembled monolayers (SAM) [9,10,11,12]. The SAMs are defined as 2D surfaces semirigid polycrystalline molecules covalently attached to a suitable substrate, giving the surface a new chemical identity [13,14,15,16]. Molecules capable of forming SAMs are constituted by three essential parts: a head group that strongly bonds to the substrate; one tail (or terminal) group representing the outer surface; and a spacer separating the head group of the terminal group, which can be either an aliphatic chain or an aromatic moiety or even mixed [17,18,19].

Heretofore, it is common to have SAMs anchored to coinage metals through a thiol (–SH) as a head group [17,19], probably due to their ease of preparation and stability [5,20,21,22]. However, very recently, some alternatives have been sought for the anchor atom aiming to replace sulfur (S), where the first candidates are those elements that belong to the same group of chalcogens; specifically, selenium (Se) and tellurium (Te) [19,20,21,22,23,24,25,26,27,28,29,30,31,32]. This choice seemed to be appropriate if we consider that the affinity of S with Au depends on the electronic structure of the valence shell of the anchor atom [24]. Presently, there is experimental evidence exposing that the stability of SAMs prepared with Se is higher than their analog with S [24,25,26,27,28]. Even though some authors point out that the Se-Au interaction is stronger than S-Au, others indicate that the bond strength of S-Au is higher than their Se analogs generating controversy regarding this subject [33,34,35,36]. On the other hand, Te-based SAMs oxidize rapidly after their formation. They have limited stability, despite the strong interaction that this anchor atom can form with the substrate [33]. However, the addition of different functional groups in the *meta* position of the aromatic ring, for example, could partially protect the Te atom towards oxidation but without significantly affecting its interaction with the gold surface.

The disagreements, as mentioned above, can be attributed to the use of different aliphatic or aromatic systems, where the structural variability among the studies is high in both experimental and theoretical studies [17,18,19]. However, most of these discrepancies could be solved by a systematic analysis of the effect of the chemical structure of the SAM subunit has on the anchor atom. To partially explore the source of these discrepancies, we have recently reported two theoretical studies of systems that describe the nature of the chalcogen interaction with gold substrates, where the chalcogen was in three possible states: protonated, anionic, and radical. In both studies, we used *para*-phenyl substituted chalcogenide ligands R*_p_*-Ph-E and a nanocluster of 42 gold atoms as a model for the gold surface (111) [35,36]. Our results pointed out that the strongest E···Au interaction is achieved by Te–Au complexes, followed by Se–Au, and finally, S–Au. In all cases, the decomposition of the total interaction energy (ΔE_int_) of the E···Au interaction indicated a dominant contribution coming from electrostatic and covalent components, and one-third from dispersive contribution. On the other hand, we found that the anionic state of the ligands is the one able to form the most stable complexes. It was very promising as we also found that the E···Au interaction strength of the anionic state of the ligands can be modulated by the modification of the substituent in *para-* position of the phenyl system. In that study, we obtained differences in the interaction energy of up to 20 kcal/mol between systems with the same chalcogenide but with a different electron-donor (–NH_2_, –OCH_3_, etc.) and electron-withdrawing substituents (–NO_2_, –CN, etc.). We also performed charge transfer analyses, from which we found that the magnitudes of charge transferred decreases in the order Te > Se > S, which agrees with the data observed experimentally [36]. One point that has not been explored to this date in the area of gold SAMs using chalcogen as head and aromatic group as a spacer is the effect that a functional group in the spacer can have depending on its position and how this could be exploited for different applications.

Herein, we aim to determine the role of inductive and steric effects that the R group in *ortho* and *meta* positions has in the E···Au interaction strength through an exhaustive computational chemistry study. For this, we used substituents with electron-donor and electron-withdrawing character in either position and focused on the anionic state of the chalcogen. Also, we studied an additional set of multi-substituted ligands for the sake of completeness in the series here studied. Finally, we present a short comparison with our previous results on the *para*-substituted complexes to provide a complete picture of the E···Au interaction aiming to remark the significant advantages of using an aromatic anchor system for SAM preparation with selenium, which at this date have not been properly exploited.

## 2. Models, Computation, and Methodology

The system used to model the surface of Au(111) was a closed shell nanocluster of 42 Au atoms (Au_42_) constituted by three layers of 14 atoms each. This nanocluster has proved to be a suitable system to model complex interactions of compounds with gold nanomaterials providing reasonable accuracy to reproduce experimental results [17,18,19]. As in our previous studies, we incorporate sulfur-, selenium-, and tellurium-based ligands, focused in their anionic state [35,36]. A set of 10 substituents (R = –NH_2_, –OCH_3_, –CH_3_, –H, –F, –Cl, –OCOCH_3_, –CF_3_, –CN, –NO_2_) were chosen to provide a wide range of electron-withdrawing and electron-donor capacities to determine their effects on the E···Au (ΔE_int_) interaction strength.

**Figure 1:**
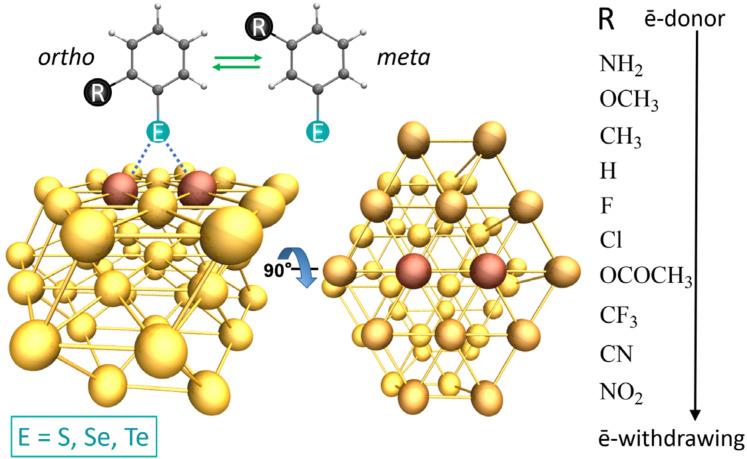
Schematic representation of the gold molecular system and the ligands used to model the gold-chalcogen interaction. The chalcogen atoms are represented by the sphere in cyan and it is pictured in the *ortho* substituted system (**left**) and *meta* substituted (**right**).

Thereby, each phenyl-chalcogenolate included a series of ligands with the substituent in *ortho* or *meta* positions in the phenyl ring (20 per chalcogen atom), thus providing a whole set of 60 ligands. Afterward, these were compared with their corresponding analogs substituted in *para* positions studied in our previous works [35,36]. To define an upper limit in the increase of the interaction strength driven by electron-donor substituents, we incorporated ligands with two and three –NH_2_ in several combinations for the substitutions in the phenyl ring, thus adding nine more ligands to the set. The starting structure of each complex was generated by placing the ligands on the upper face of the Au_42_ nanocluster (see Figure 1). All geometry optimizations were carried out using the software Turbomole version v.7.0 [37]. From a previous study, we demonstrated that the relaxation of the surface of the cluster due to the interaction with the ligand increases the interaction energy by only 1.6 kcal/mol, but exceedingly increasing the computational time [36]. Besides, the increase was similar for all the tested ligands, thus not affecting the conclusions drawn from the results. Therefore, the gold nanocluster (Au_42_) was fixed during the optimizations, allowing only the ligand to relax freely on the surface of the cluster, because of the extensive set of ligands here studied (60 mono-substituted and 9 multi-substituted). The calculations were carried out using density functional theory (DFT) in conjunction with the meta-GGA TPSS density functional [38], which has proven suitable to model these types of interactions by previous studies [39,40]. The ligands and the first layer of the nanocluster of Au_42_ were modeled using the def2-TZVPD [41] basis set while the second and third layers of the cluster were modeled using the def2-SVP basis set [42]. Additionally, Effective core pseudopotentials (ECPs) with 19 valence electrons (VEs) were used for all Au atoms [43,44]. ECPs were also employed in the case of Se and Te [45]. The atoms H, C, N, O, F, S, and Cl were treated with all their electrons. The Grimme dispersion correction [46,47,48] was used to improve the description of the E···Au interaction and its use is indicated by “DFT-D3”. The resolution of the identity (ri) [49,50] was also used to increase the computational efficiency of the calculations. The evaluation of the E···Au interaction energy was calculated by subtracting the sum of the Au_42_ nanoclusters and the corresponding ligand from the energy of the complex. To eliminate the basis set superposition error (BSSEs), the calculations of the interaction energies were corrected by the counterpoise correction. We carried out the natural population analysis (NPA) [51] to analyze the charge transfer process taking place in the formation of the study complexes. The energy decomposition analysis (EDA) was performed based on the Morokuma-Ziegler [52] partitioning scheme to obtain a clear picture of the contributions to the E-Au interaction, implemented in the ADF code [53]. This scheme takes into account that the interaction energy could decompose into an orbital and steric contribution (ΔE_int_ = ΔE_orb_ + ΔE_steric_) with an orbital contribution as a dominant stabilizing factor due to its covalent character, while the steric factor appears to destabilize. The latter is calculated as the sum of the electrostatic interaction (ΔE_elstat_), which stabilizes, and the Pauli repulsion (ΔE_Pauli_) destabilizer and principle contribution to the steric interaction. The TZP/TPSS was used, accompanied by the two component zero-order regular approximation (ZORA) [54] Hamiltonian to take the relativistic effects into account. Finally, for some selected cases in which it was necessary a clear picture of the contribution and topology of the non-covalent interactions, we calculated the non-covalent interaction index (NCI) [55,56]. From this analysis, it is possible to obtain a representation in real space of the non-covalent interactions taking place between the ligands and Au_42_; also, distinguishing between attractive and repulsive interactions. For interpretative purposes, the regions of the surface colored in blue denote strong stabilizing interactions, green indicates weak interactions usually associated with van der Waals interactions, and the colored in red are indicative of repulsive interactions. This type of analysis is also complementary to the SAPT approach for gold molecules [57].

## 3. Results and Discussion

### 3.1. Binding Mode Conformation

**Figure 2:**
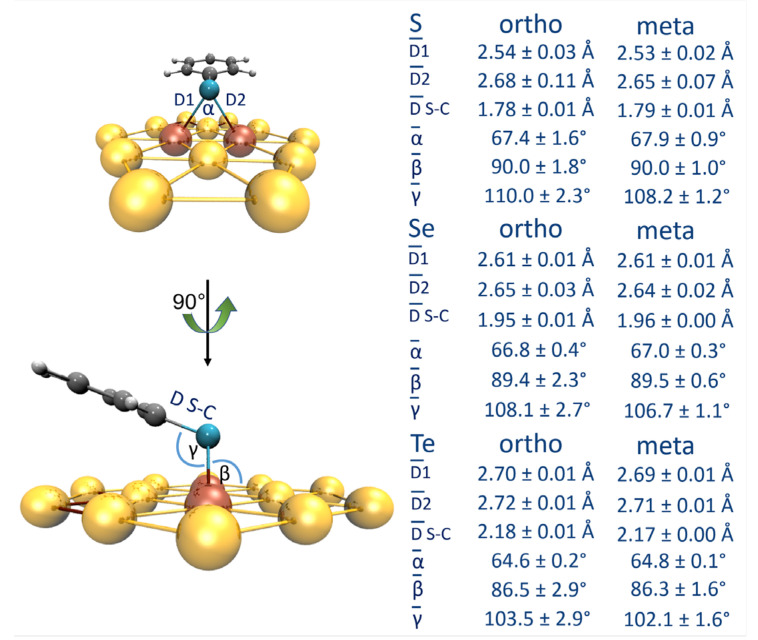
Graphic representation of the principal geometric parameters obtained for the systems with the substituent R in the *ortho* and *meta* positions (hydrogen-white, carbon-grey, gold-yellow and orange, chalcogen-cyan).

The absorption of the anion ligands substituted in the *meta* phenyl position on the surface of the Au_42_ clusters R_m_–Ph–E^−^∙∙∙Au_42_ (E: S, Se, and Te) is mainly driven by the interaction between the chalcogen atom and two gold atoms from the surface. The anchor atom E is positioned above the Au–Au bond at the center of the cluster adopting a bridge conformation. The distances between the chalcogen and each of the gold atoms are indicated by D_1_ and D_2_, where the former corresponds to the shorter E-Au distances and the latter to the largest. The Au–E–Au angle is defined as α, and the inclination of the plane containing the α angle concerning the Au(111) plane is the β angle. These parameters are described and summarized in Figure 2. The complete list of geometric data of the systems formed between the Au_42_ clusters and the anion ligands R–Ph–E^−^∙∙∙Au_42_ are listed in Tables S1–S6 of the supporting information.

For analysis purposes, we have calculated the average values of D̅_1_ and D̅_2_ distances and the standard deviation to determine the degree of variation among the series. The shallow values obtained for the standard deviation between these systems indicate that the structural configuration of the complexes is not affected by the electron donor or withdrawing character of the substituent. The interaction distances were very similar to the previously obtained for the systems *para-*substituted [35,36], showing that the change in the position of the substituent does not affect the interaction region. The values of D̅_1_ and D̅_2_ distances for the three chalcogen systems were as follows: 2.63 and 2.54 Å with S; 2.65 and 2.60 Å with Se; and 2.70 and 2.72 Å with Te, exposing that Se and Te arrived at more symmetrical interaction configurations, while S seems to interact strongly with one of the Au atoms. The resulting E-C distances for the *meta* substituted ligands were 1.79, 1.96, and 2.17 Å, for S, Se, and Te, respectively, following the increase of the chalcogen size. The angle α decreases as it passes from S to Se and then Te, which is also a consequence of the rise in the size of the chalcogen. The evolution of the β angle between the series indicates that the ligand R_m_ -Ph-E^−^ arranges more parallel to the surface of the Au_42_ clusters as we change the chalcogen in the order S→Se→Te.

The systems R_o_–Ph–E^−^∙∙∙Au_42_ with the substituent R located in the *ortho* phenyl position showed the same conformation of interaction with distances D̅_1_ and D̅_2_ of 2.67 and 2.55 Å for S, 2.62 and 2.61 Å for Se, and 2.70 and 2.72 Å for Te. As with the previous cases with R in *meta* position, the values of the standard deviation shown in Figure 2 indicate that the structural conformation of the complexes is not affected by the character of the substituent. The E-C distances, together with α and β, angles showed the same trends as the systems with R in *meta* position. At this point, it is possible to generalize that independent of the substituent position or the chalcogen, the anionic form of this SAM subunit adopts a bridge conformation when interacting with gold substrates.

### 3.2. Interaction Strength Analysis

**Figure 3:**
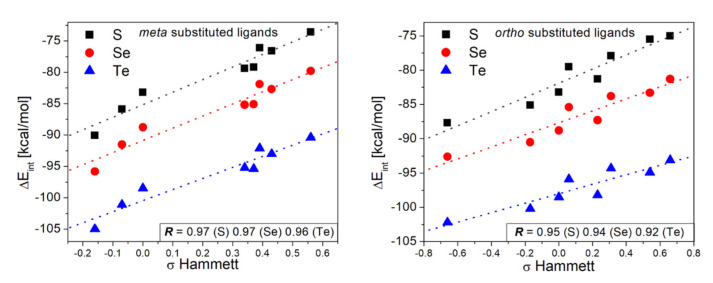
Plot of the interaction energies of the phenyl-chalcogenolates functionalized in *meta* (**left**) and *ortho* (**right**) positions versus the Hammett sigma constants σ_meta_ and σ_ortho_.

The interaction energies for the complexes [R_m_–Ph–E^−^∙∙∙Au_42_]^−^ (E = S, Se y Te) with R in the *meta* position are listed in Table 1. The three series of complexes presented the highest interaction energy when the phenyl group was functionalized with –NH_2_, with −90.1, −95.8, and −105.0 kcal/mol, for S, Se, and Te, respectively. Meanwhile, the lowest interaction energy for the three chalcogens was obtained with –CN. According to this, the ranges of variation between the highest and the lowest interaction energy were 16.5 for S, 16.0 for Se, and 14.6 kcal/mol for Te. The contribution of the dispersion forces to this interaction within the series was ~30–40% of the total interaction energy. Despite the significant contribution of the dispersion forces, there is a dominant covalent contribution to the Au-E interaction, which comprises 60–70% of the total interaction strength. To obtain insights about the relevance of the electron-donor or acceptor character on the interaction of the ligand with the gold substrate, the ΔE_int_ was plotted as a function of the sigma Hammett for *meta* substituted phenyl rings (σ_m_). In this particular context, we use the sigma Hammett constants to determine if there is a correlation between the binding process and the nature of the substituents. The results show a close-to-linear behavior, whose linear fit provided a Pearson correlation coefficient (*R*) of 0.94 for the three chalcolgens. From the set of ligands, –OCH_3_ and –NO_2_ seem to fall out of the trend, and their removal from the linear fit increased the correlation coefficient *R* to 0.97 for S and Se; while for Te increased to 0.96 as shown in Figure 3. The plots with the complete set of data are presented in Figure S1. The removal of the –NO_2_ ligand is justified by the fact that this ligand incorporates an additional contribution to the interaction strength, and its interaction strength is not purely due to the chalcolgen-gold interaction as observed for the rest of the ligands included in this study. For the –OCH_3_ ligand, we will see in the following section how the particular ability of the substituent to adapt its conformation favors the interaction energy and the covalent contribution. For the ligand with –NO_2_, it was expected to have the lowest interaction energy as it has the most electron-withdrawing group; however, its interaction strength was higher than with –CN as a functional group. This increase to –CN was due to a higher contribution of the dispersion term as exposed from the data in Table 1. In fact, the linear fit of the interaction energies without the dispersion contribution with the complete series has an *R* value of 0.97, indicative of the disrupting effect of dispersion on the correlation coefficient. Thereby, according to our results, the interaction strength can be modulated to some extent through the use of donor-acceptor effects at the *meta* position.

The interaction energies for the complexes functionalized in the *ortho* position [R_o_–Ph–E^−^∙∙∙Au_42_]^−^ are listed in Table 2. The systems with S presented interaction energies ranging from −75.0 to −89.3 kcal/mol, respectively, with a range of variation of 14.3 kcal/mol. Meanwhile, Se showed interaction energies that range between −81.3 kcal/mol and −95.1 kcal/mol, resulting in a range of variation of 13.8 kcal/mol. The same trend was reproduced with Te, with the lowest and the highest interaction energies of −93.1 and −105.3 kcal/mol, respectively, where its range of variation was 12.2 kcal/mol. In all three cases, the extreme interaction energies within the range of variation were obtained with –OCH_3_ instead of –NH_2_, and –CN in the lowest limit as substituents. Meanwhile, the dispersion contribution was ~32–44% across the series, which as in the case of the systems with the substitution in *meta*, points out the involvement of covalent contribution to the E∙∙∙Au interaction.

**Figure 4:**
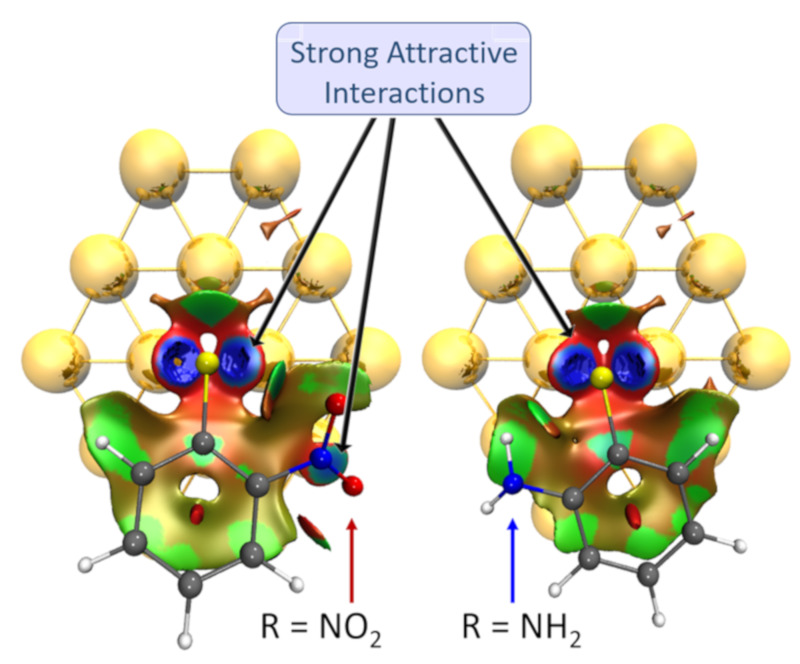
Graphical representation of the non-covalent interactions between the gold substrate and the phenyl-sulphonate ligands functionalized in *ortho* with –NO_2_ and NH_2_ substituents. (hydrogen—white; carbon—grey; sulfur—yellow; nitrogen—blue and the isovalue used as cutoff was defined at 0.5).

As with *meta* substituted ligands, the donor-acceptor character of the functionalization at *ortho* position was analyzed through the correlation between the ∆E_int_ and the sigma-Hammett for *para* (σ_p_) substituted phenyl rings, results shown in Figure 3. The use σ_p_ is based on the fact that both *ortho* and *para* positions provide similar resonance effects; however, care should be taken as there are other effects due to the proximity of the *ortho* position with the interaction interface. The calculated correlation coefficients *R* were of 0.92, 0.88, and 0.75 for S, Se, and Te; respectively. These results show a decrease in the relationship between donor-acceptor effects and the interaction strength, which is even lower for Se and Te ligands. These results point out that with the increase in the interaction strength by replacing S with Se or Te, a more significant local effect of the functional group is observed functioning as an indirect indicator of the steric and inductive effects on the interaction energy when functionalized in *ortho* position. These inductive effects can be understood in terms of dipole–dipole and charge–dipole interactions. A qualitative picture of the role of inductive effects is obtained if we assume that electron-withdrawing groups stabilize the negative charge of the chalcogenide anion. The removal of –OCH_3_ and –NO_2_ from the linear fit increased *R* to 0.96, 0.94, and 0.92 for S, Se, and Te; respectively. As observed for the *meta* substituted ligands, the ligand with –NO_2_ also has a larger contribution from dispersion than –CN and the rest of the ligands. Among the series, the S-based ligands showed a more considerable dispersion contribution, probably because of the closer proximity with the gold substrate for Se and Te, as the interaction distances increased with these two latter atoms. Therefore, to understand the source of the higher contribution of dispersion with NO_2_, we analyzed its interaction with the gold surface by using a non-covalent index (NCI) considering the S-based ligands functionalized in *ortho* position (highest dispersion contribution), results shown in Figure 4. We compared the ligand functionalized with –NO_2_ with the one substituted by –NH_2_, as the latter showed a lower dispersion contribution. The blue region indicates a strong interaction, according to which both complexes showed a strong non-covalent interaction region at the gold–ligand interface between S and two gold atoms, reinforcing the bridge nature of the interaction. However, between the NO_2_ group and the gold surface, there exists an additional strong non-covalent interaction that is missing in the NH_2_ complex. This effect was not observed for any of the other ligands (data not shown), denoting the exciting ability of the –NO_2_ group to interact with the gold substrate. Point aside, the behavior observed for the ligand with –OCH_3_ in *ortho* position was not related to the dispersion contribution, being the only possibility a source of the covalent character of the interaction as it will be detailed in the next section.

Upon comparing the ranges of variation of the systems functionalized in *ortho* and *meta* with those obtained for the systems with the substitutions in *para* position [35,36] (R_p_–Ph–E^−^∙∙∙Au_42_: 23.8, 21.6, and 19.4 kcal/mol, for S, Se, and Te, respectively), we observed an apparent decrease in what we define as the modulation range. This reveals that the position of the substituent may be chosen according to the application in which this molecular scaffold will be utilized. Finally, the order of the effect of donor-acceptor character on the interaction strength modulation follows the trend *para* > *meta* > *ortho.*

### 3.3. Energy Decomposition Analysis

To improve the understanding of the role of the substituent in the nature of the interaction between the chalcogen ligands and the gold substrate, we performed an energy decomposition analysis of the interaction energies. It was performed only for the extreme cases represented by the ligands functionalized with –NH_2_, –OCH_3_, –CN, and –NO_2_; data listed in Table 3 for the *meta* and *ortho* substituted ligands. Expressing the stabilizing contributions in terms of percentage the identification of the component responsible for the increase in the interaction strength when replacing S by Se or Te. The results reveal that in both *meta* and *ortho* substituted ligands the growth of the interaction strength is dominated by an increase in the electrostatic contribution as we replace S with Se or Te, which according to our results, is responsible for the favorable nature of the interaction.

**Figure 5:**
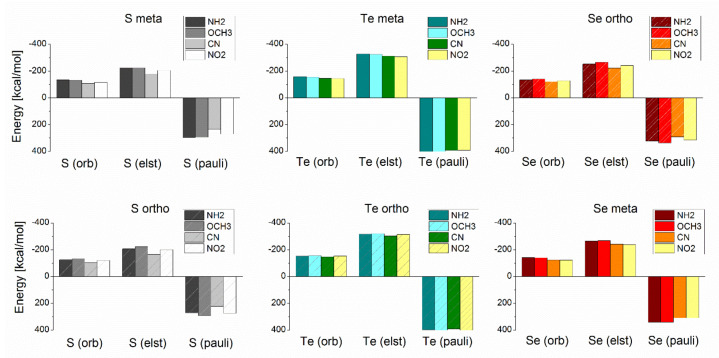
Comparison of the different components to the covalent contribution to the interaction energies obtained from the energy decomposition analysis of *ortho* and *meta* substituted ligands with –NH_2_, –OCH_3_, –CN, and –NO_2_ as functional group.

Through EDA analysis, we also found that for the *ortho* series, the ligand with –OCH_3_ presented higher interaction energy concerning –NH_2_, because the former ligands have a higher total orbital contribution (ΔE_orb_) (Figure 5). In principle, because of the resonance effect, a higher orbital contribution may be expected for the *ortho* substituted ligands than for the *meta* substituted. However, it becomes clear that the presence of the functional group does not allow adopting a closer interaction with the gold substrate and thus interfering with the interaction. Consequently, along with this series, we obtained slightly higher interaction energies for the *meta* ligands than with *ortho* ligands. However, the only exception was with –OCH_3_, something that we attribute to the unique ability of this substituent to rotate and decrease the steric clash with the gold surface, allowing the resonance effect to take part and increasing the orbital contribution as observed from our results.

### 3.4. Charge Transfer Analysis

When the ligand is in its anionic state, these act as charge donors, while the gold cluster acts as a charge acceptor, property that defines the nature of the interaction. The series of substituents used in this study were selected to determine the extent to which the interaction strength can be modulated by controlling the amount of charge donated by the ligand to the substrate to obtain insights about its relationship with the interaction energy. To explore this specific feature of the interaction process, we have carried out the NPA analysis to quantify the charge transfer process for the [R_m_–Ph–E^−^∙∙∙Au_42_]^−^ and [R_o_–Ph–E^−^∙∙∙Au_42_]^−^ complexes. The results obtained are listed in Table 4 and Table 5 for *meta* and *ortho* substituted ligands, respectively. The data is provided as the amount of charge donated to the gold cluster, and the difference of charge in the chalcogen atom before and after the binding to the substrate; the latter aiming to determine the ability of each chalcogen to donate charge and to which extent is affected by the functional group. In general, the amount of charge donated to the substrate correlated to the electron-donor and withdrawing properties of each substituent, where the ligands functionalized with –NH_2_ was able to transfer the largest amount of charge, whereas those with –NO_2_ the lowest. However, the increase of charge transfer from the non-substituted ligand (R=H) to the one with –NH_2_ was of only 0.03e and 0.04e for the *meta* and *ortho* S-substituted ligands, respectively; increase that was even lower for Se and Te ligands. This indicates that controlling the increase in charge transfer is not easily modulated. Meanwhile, the decrease of charge transfer from the non-substituted to the ligand with -NO_2_ involves a reduction in 0.12e and 0.13e for *meta* and *ortho* S-substituted ligands, respectively; revealing a higher degree of flexibility concerning the control of decreasing the charge transfer. Regarding the intrinsic properties of the chalcogens, the exchange between chalcogens from S to Se and then Te is followed by a continuous increase in the charge donated to the substrate. These results expose the ability of Se and Te to donate more significant amounts of charge when compared to the commonly used S-based ligands.

**Figure 6:**
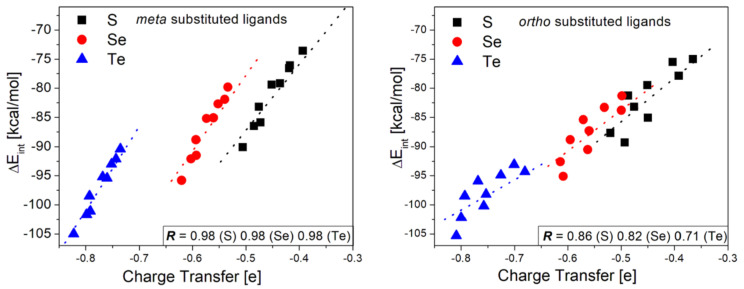
Plot of the amount of charge transferred to the gold substrate from the phenyl-chalcogenolates functionalized in *meta* (**left**) and *ortho* (**right**) positions.

An interesting correlation was obtained when analyzing the relationship between the interaction energies and the amount of charge transferred for *meta* substituted ligands. Because the ligand with –NO_2_ as a substituent seems to behave differently to the rest of the ligands due to its higher dispersion contribution, this was removed from this analysis. However, the plots with the complete set of data are presented in Figure S2. After linear fitting, we obtained a correlation coefficient *R* of 0.98 for the three chalcogens as shown in Figure 6, revealing a high degree of correlation between the interaction strength and the amount of charge transferred to the substrate. This result perfectly complements our analysis provided by the energy decomposition analysis. As explained above, the interaction strength is dominated by a rise in the electrostatic contribution to the interaction energy, which is expected to increase with the increase of the difference of charge between the ligand and the gold substrate. Thereby, this difference of charge and thus the electrostatic contribution depends on the amount of charge transferred from the ligand to the substrate. Results provide a detailed picture of the interaction mechanism. Meanwhile, the *ortho* substituted ligands do not show the same correlation, because there is a steric component involved that prevents a proper approaching of the chalcogen atom to the surface. In addition to the lack of relationship, the *meta* substituted ligands were able to transfer more charge than the functionalized in *ortho*, although the differences were low. If we consider the effects of functionalizing in *para* position from results previously reported [35,36], the position that better allows modulating the charge transfer is the *para* position, followed by meta, with *ortho* being the less favorable position to functionalize the ligand.

### 3.5. Multi-Substituted Selenophenolate Ligands

**Figure 7:**
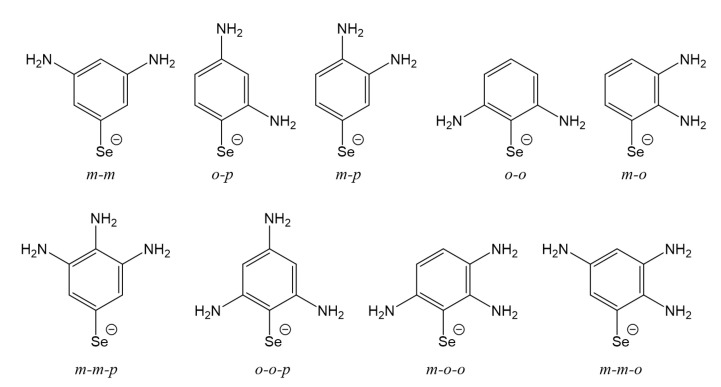
Representation of the systems used to assess the effect in the interaction energy of multi-substitution of phenyl-selenophenolate ligands with two and three NH_2_ functional group.

For the sake of completeness, the final assessment would be to test the effect of multi-substitution. According to our results, the increase of the interaction energy is dominated by the charge transfer process, responsible for the increase in the electrostatic contribution. However, it turned out that there are limitations regarding the rise of the charge transfer using electron-donating groups. Therefore, we focus on the incorporation of one and two additional –NH_2_ as an electron-donor group in different combinations to determine the ability to modify the interaction strength and somehow to establish an upper limit for this property. For this purpose, we selected selenium-based ligands as these represent the best alternative to sulfur based-ligands, because of their stability and lower toxicity than tellurium for biological applications. The list of ligands and their interaction energies are presented in Table 6. The ligands used for this part of the study are shown in Figure 7.

The complexes formed with the multi-substituted ligands adopted the bridge conformation as expected from our results for anionic chalcogenide ligands. The geometric parameters listed in Table S7 show a generalized increase in the interaction distance compared to the mono-substituted ligand with –NH_2_. This seems to be a consequence of the increase of the steric effects coming from the larger size of these ligands. In terms of interaction energies (see Table 6), the ligands with three substituents achieved higher interaction energies than the ones with two. However, all of the ligands with two substituents presented lower interaction energies compared with the selenophenolate ligand substituted in *meta* position with the analogous –NH_2_, which has interaction energy of −95.8 kcal/mol. The highest interaction energy was obtained with the combination *meta-meta* from the bi-substituted ligands, while the lowest with the *meta-ortho*. From the tri-substituted, only the combinations *ortho-ortho-para* and *meta-meta-para* achieved higher interaction energies than the mono-substituted ligand above mentioned (*meta*–NH_2_). However, the increases were only of 0.5 kcal/mol and 1.5 kcal/mol respectively, denoting the difficulties in increasing the interaction strength, it would be necessary for any specific application. The EDA analysis for the multi-substituted ligands, as shown in Table S8 denotes the same behavior with the electrostatic contribution being the dominant stabilizing interaction. The low increase in the interaction energy after the incorporation of new –NH_2_ groups, was also reflected in the moderate increase in the charge transfer as denoted from the results presented in Table S9. From these data, a maximum increase of 0.14e is found when comparing the *ortho-ortho-para* and the *meta*–NH_2_ ligand.

## 4. Conclusions

We described of the physicochemical components that contribute to the interaction between phenyl chacogenolate ligands with gold-based nanomaterials. The nature of this interaction comprises 30–40% of dispersion forces, while the rest corresponds to a covalent contribution dominated by electrostatic interaction. Interestingly, this electrostatic interaction is triggered by the charge transfer process that takes place during the complex formation. Thereby, the modulation of the amount of charge transfer by using electron-donor or withdrawing groups leads to control of this electrostatic component, and thus of the interaction strength. In this context, functionalization in *ortho* position involves inductive and steric effects that prevent a systematic control of this interaction. However, the use of systems functionalized in *ortho* position may act as a protective system against the oxidation of Te-based ligands, which can be advantageous when very high interaction energies are needed for example in the design of electronic devices. The use of multisubstituted ligands proved that there is a limit in which this interaction can be strengthened. On the other hand, there is no substantial advantage in using multi substituted versus monosubstituted ligands in this regard. As a concluding remark, the magnitudes of the interaction strength in terms of the position of functionalization and independent of the chalcogenide followed the trend *para*→*ortho*→*meta*, which could be used for the design of new devices, where Se and Te represent an interesting and promising new alternative to S-based systems.

## Figures and Tables

**Table 1:** Interaction energies between Au_42_ and *ortho* substituted chalcogen (E = S, Se, Te). The energies listed correspond to the total interaction energies (ΔE_int_(TPSS-D3)) and without the dispersion contribution (ΔE_int_(TPSS)). The percentage of dispersion term is included. All energies are in kcal/mol.

[Au_42_-EPhR_o_]^−^	ΔE_int_(TPSS-D3)			ΔE_int_(TPSS)			% Dispersion		
	S	Se	Te	S	Se	Te	S	Se	Te
Au_42_-EC_6_H_4_(NH_2_)_o_	−87.7	−92.6	−102.2	−55.4	−59.2	−66.6	36.8	36.0	34.8
Au_42_-EC_6_H_4_(OCH_3_)_o_	−89.3	−95.1	−105.3	−57.1	−61.2	−69.3	36.1	35.7	34.1
Au_42_-EC_6_H_4_(CH_3_)_o_	−85.1	−90.5	−100.2	−54.4	−58.0	−65.3	36.1	35.9	34.8
Au_42_-EC_6_H_5_	−83.2	−88.8	−98.5	−55.4	−59.1	−66.7	33.4	33.5	32.3
Au_42_-EC_6_H_4_(F)_o_	−79.5	−85.4	−95.9	−51.4	−55.5	−64.1	35.4	35.0	33.2
Au_42_-EC_6_H_4_(Cl)_o_	−81.3	−87.3	−98.2	−50.0	−54.0	−62.7	38.5	38.1	36.1
Au_42_-EC_6_H_4_(OCOCH_3_)_o_	−77.9	−83.8	−94.3	−43.8	−48.1	−57.2	43.7	42.6	39.4
Au_42_-EC_6_H_4_(CF_3_)_o_	−75.5	−83.3	−94.9	−45.3	−51.7	−61.3	40.0	37.9	35.4
Au_42_-EC_6_H_4_(CN)_o_	−75.0	−81.3	−93.1	−43.6	−48.1	−57.3	41.8	40.9	38.5
Au_42_-EC_6_H_4_(NO_2_)_o_	−76.7	−84.3	−97.8	−42.9	−48.5	−59.5	44.0	42.5	39.1

**Table 2:** Interaction energies between Au_42_ and *meta* substituted chalcogen (E = S, Se, Te). Energies listed correspond to the total interaction energies (ΔE_int_(TPSS-D3)) and without the dispersion contribution (ΔE_int_(TPSS)). Percentage of dispersion term is included. All energies are in kcal/mol.

[Au_42_–EPhR_o_]^−^	ΔE_int_(TPSS-D3)			ΔE_int_(TPSS)			% Dispersion		
	S	Se	Te	S	Se	Te	S	Se	Te
Au_42_-EC_6_H_4_(NH_2_)_m_	−90.1	−95.8	−105.0	−58.7	−62.5	−69.4	34.9	34.7	33.9
Au_42_-EC_6_H_4_(OCH_3_)_m_	−86.5	−92.1	−101.7	−55.7	−59.5	−67.0	35.6	35.4	34.1
Au_42_-EC_6_H_4_(CH_3_)_m_	−85.9	−91.5	−101.1	−55.6	−59.6	−67.3	35.3	34.9	33.5
Au_42_-EC_6_H_5_	−83.2	−88.8	−98.5	−55.4	−59.0	−66.7	33.4	33.6	32.3
Au_42_-EC_6_H_4_(F)_m_	−79.4	−85.2	−95.2	−51.4	−55.4	−63.1	35.3	35.0	33.7
Au_42_-EC_6_H_4_(Cl)_m_	−79.2	−85.1	−95.4	−48.9	−53.2	−61.5	38.3	37.5	35.5
Au_42_-EC_6_H_4_(OCOCH_3_)_m_	−76.1	−81.9	−92.1	−47.0	−51.3	−59.2	38.2	37.4	35.7
Au_42_-EC_6_H_4_(CF_3_)_m_	−76.6	−82.7	−93.0	−46.9	−51.2	−59.3	38.8	38.1	36.2
Au_42_-EC_6_H_4_(CN)_m_	−73.6	−79.8	−90.4	−44.4	−49.0	−57.4	39.7	38.6	36.5
Au_42_-EC_6_H_4_(NO_2_)_m_	−74.7	−80.6	−91.0	−43.3	−47.7	−56.0	42.1	40.8	38.4

**Table 3:** Stabilizing contributions to the interaction energy calculated from EDA analysis between the ligands functionalized in *meta* and *ortho* position with the gold substrate

Model System	% Orb (*meta*)	% Orb (*ortho*)	% Elect (*meta*)	% Elect (*ortho*)
Au_42_-SC_6_H_4_(NH_2_)	37.4	37.6	62.6	62.4
Au_42_-SeC_6_H_4_(NH_2_)	34.8	34.7	65.2	65.3
Au_42_-TeC_6_H_4_(NH_2_)	32.6	32.4	67.4	67.6
Au_42_-SC_6_H_4_(OCH_3_)	37.0	37.2	63.0	62.8
Au_42_-SeC_6_H_4_(OCH_3_)	34.3	34.5	65.7	65.5
Au_42_-TeC_6_H_4_(OCH_3_)	32.3	32.7	67.7	67.3
Au_42_-SC_6_H_4_(CN)	37.6	38.7	62.4	61.3
Au_42_-SeC_6_H_4_(CN)	33.8	34.7	66.2	65.3
Au_42_-TeC_6_H_4_(CN)	31.6	32.5	68.4	67.5
Au_42_-SC_6_H_4_(NO_2_)	36.0	37.4	64.0	62.6
Au_42_-SeC_6_H_4_(NO_2_)	33.9	35.1	66.1	64.9
Au_42_-TeC_6_H_4_(NO_2_)	31.9	32.7	68.1	67.3

**Table 4:** Quantification of the charge transfer process between the *ortho*-substituted ligands and the gold-based substrate. These were obtained from the difference between the NPA charges of the complexes and the free fragments.

	*S*		*Se*		*Te*	
Substituent	Au_42_	∆S ^a^	Au_42_	∆Se ^a^	Au_42_	∆Te ^a^
-NH_2_	−0.52	0.28	−0.61	0.41	−0.80	0.65
-OCH_3_	−0.49	0.26	−0.61	0.41	−0.81	0.66
-CH_3_	−0.45	0.25	−0.56	0.39	−0.76	0.63
-H	−0.48	0.27	−0.60	0.42	−0.79	0.66
-F	−0.45	0.24	−0.57	0.39	−0.77	0.63
-Cl	−0.49	0.23	−0.56	0.36	−0.75	0.61
-OCOCH_3_	−0.39	0.18	−0.50	0.32	−0.68	0.54
-CF_3_	−0.40	0.20	−0.53	0.34	−0.73	0.58
-CN	−0.37	0.16	−0.50	0.30	−0.70	0.55
-NO_2_	−0.35	0.14	−0.46	0.27	−0.66	0.50

[^a^] Values represent the amount of charge donated directly by the chalcogen atom and it was obtained from the difference between the chalcogen charge in the complex and the free ligand.

**Table 5:** Quantification of the charge transfer process between the *meta*-substituted ligands and the gold-based substrate. These were obtained from the difference between the NPA charges of the complexes and the free fragments.

	*S*		*Se*		*Te*	
Substituent	Au_42_	∆S^a^	Au_42_	∆Se ^a^	Au_42_	∆Te ^a^
-NH_2_	−0.51	0.27	−0.62	0.42	−0.82	0.67
-OCH_3_	−0.48	0.27	−0.60	0.42	−0.80	0.66
-CH_3_	−0.47	0.27	−0.59	0.42	−0.79	0.66
-H	−0.48	0.27	−0.59	0.42	−0.79	0.66
-F	−0.45	0.25	−0.57	0.40	−0.77	0.64
-Cl	−0.44	0.23	−0.56	0.38	−0.76	0.63
-OCOCH_3_	−0.42	0.22	−0.54	0.36	−0.74	0.62
-CF_3_	−0.42	0.22	−0.55	0.38	−0.75	0.63
-CN	−0.39	0.21	−0.53	0.36	−0.74	0.61
-NO_2_	−0.36	0.20	−0.49	0.34	−0.71	0.58

[^a^] Values represent the amount of charge donated directly by the chalcogen atom and it was obtained from the difference between the chalcogen charge in the complex and the free ligand.

**Table 6:** Interaction energies between Au_42_ and multi-substituted selenophenolates. The energies listed correspond to the total interaction energies (ΔE_int_(TPSS-D3)) and without the dispersion contribution (ΔE_int_(TPSS)). The percentage of dispersion term is included. All energies are in kcal/mol.

	ΔE_int_(TPSS-D3)	ΔE_int_(TPSS)	% Dispersion
(NH_2_)m-m-p	−97.3	−71.0	37.2
(NH_2_)o-o-p	−96.3	−68.7	40.2
(NH_2_)m-o-o	−95.3	−67.0	42.4
(NH_2_)m-m-o	−94.5	−67.3	40.5
(NH_2_)m-m	−93.0	−68.7	35.3
(NH_2_)o-p	−92.9	−68.6	35.4
(NH_2_)m-p	−91.8	−68.1	34.8
(NH_2_)o-o	−90.7	−64.7	40.2
(NH_2_)m-o	−87.9	−62.2	41.3

## Supplementary Materials

The following are available online at https://www.mdpi.com/2079-4991/10/6/1237/s1, Table S1: Selected geometric parameters for the anionic *meta* substituted thiophenolate-gold complexes; Table S2: Selected geometric parameters for the anionic *meta* substituted selenophenolate-gold complexes; Table S3: Selected geometric parameters for the anionic *meta* substituted telurophenolates-gold complexes; Table S4: Selected geometric parameters for the anionic *ortho* substituted thiophenolate-gold complexes; Table S5: Selected geometric parameters for the anionic *ortho* substituted selenophenolate-gold complexes; Table S6: Selected geometric parameters for the anionic *ortho* substituted telurophenolates-gold complexes; Table S7: Selected geometric parameters for the anionic multi-substituted selenophenolate-gold complexes; Table S8: Energy decomposition analysis of the interaction energies calculated for the multisubstituted ligands. The data is presented as percentages of contribution to the stabilizing covalent component, Table S9: Quantification of the charge transfer process between the multi-substituted ligands and the gold-based substrate. These were obtained from the difference between the NPA charges of the complexes and the free fragments. Figure S1. Plot including the complete series of the interaction energies of the phenyl-chalcogenolates functionalized in *meta* (left) and *ortho* (right) positions versus the Hammett sigma constants σ_meta_ and σ_ortho_. Figure S2. Plot including the complete series of the amount of charge transferred to the gold substrate from the phenyl-chalcogenolates functionalized in *meta* (left) and *ortho* (right) positions. All coordinates from the chalcogen-based ligands systems.
