# Supplementary material for: Exploration of the Interaction Strength at the Interface of Anionic Chalcogen Anchors and Gold (111)-Based Nanomaterials
Source: Nanomaterials (Basel). 2020 Jun 25;10(6):1237. doi: 10.3390/nano10061237 (PMC7353086; doi:10.3390/nano10061237)
Supplement: Supplementary file 1 [file nanomaterials-10-01237-s001.pdf]

Exploration of the Interaction Strength at the Interface of Anionic Chalcogen Anchors and Gold (111)-Based Nanomaterials

Sebastián Miranda-Rojas[1,*] and Fernando Mendizabal[2,*]

[1] Departamento de Ciencias Químicas, Facultad de Ciencias Exactas, Universidad Andres Bello, Av. República 275, Santiafo (Chile) PO 8370146; sebastian.miranda@unab.cl

[2] Departamento de Química, Facultad de Ciências, Universidad de Chile, Lãs Palmeras 3425, Ñuñoa, Santiago (Chile) PO 7800003; hagua@uchile.cl

* Correspondence: sebastian.miranda@unab.cl (S.M-R); hagua@uchile.cl (F.M).

1. Table S1………………………………………………………………… 2
2. Table S2…………………………………………………………………3
3. Table S3…………………………………………………………………4
4. Table S4…………………………………………………………………5
5. Table S5…………………………………………………………………6
6. Table S6…………………………………………………………………7
7. Table S7…………………………………………………………………8
8. Table S8…………………………………………………………………9
9. Table S9…………………………………………………………………10
10. Figure S1……………………………………………………………...11
11. Figure S2……………………………………………………………12
12. Coordinates from the Sulfur-based Ligands (META)………………………13
13. Coordinates from the Sulfur-based Ligands (ORTHO)……………………..33
14. Coordinates from the Selenium-based Ligands (META)…………………...53
15. Coordinates from the Selenium-based Ligands (ORTHO)…………………74
16. Coordinates from the Tellurium-based Ligands (META)…………………...93
17. Coordinates from the Tellurium-based Ligands (ORTHO)…………………114

Table S1. Selected geometric parameters for the anionic *meta* substituted thiophenolate-gold complexes[a] (distances in Å and angles in deg).

**Au_{42}-Thiophenolate (S^-)**	**D_1**	**D_2**	**$D_{C\text{-}S}$**	**α**	**β**	**γ**
Au_{42}-$SC_6H_4NH_2$	2.56	2.62	1.79	68.0	90.6	106.7
Au_{42}-$SC_6H_4OCH_3$	2.52	2.62	1.80	68.5	91.7	109.6
Au_{42}-$SC_6H_4CH_3$	2.53	2.60	1.80	68.8	90.6	108.7
Au_{42}-SC_6H_5	2.52	2.60	1.80	69.0	91.4	110.1
Au_{42}-SC_6H_4F	2.52	2.64	1.79	68.3	90.2	109.1
Au_{42}-SC_6H_4Cl	2.53	2.66	1.79	67.7	89.7	108.1
Au_{42}-$SC_6H_4OCOCH_3$	2.51	2.72	1.79	67.2	89.5	108.0
Au_{42}-$SC_6H_4CF_3$	2.52	2.67	1.79	67.8	89.0	108.0
Au_{42}-SC_6H_4CN	2.50	2.81	1.78	65.7	88.8	106.5
Au_{42}-$SC_6H_4NO_2$	2.57	2.59	1.78	68.3	88.8	107.6

[a] The nomenclature used on the column headings for the definition of the geometric parameters is detailed on Figure 2 from the manuscript.

Table S2. Selected geometric parameters for the anionic *meta* substituted selenophenolate-gold complexes[a] (distances in Ǻ and angles in deg).

**Au_{42}-Selenophenolate (Se^-)**	**D_1**	**D_2**	**D_{C-S}**	**α**	**β**	**γ**
Au_{42}-$SeC_6H_4NH_2$	2.60	2.66	1.96	66.8	88.8	104.0
Au_{42}-$SeC_6H_4OCH_3$	2.60	2.62	1.96	67.3	90.4	107.0
Au_{42}-$SeC_6H_4CH_3$	2.61	2.62	1.96	67.3	89.8	106.7
Au_{42}-SeC_6H_5	2.60	2.63	1.96	67.3	89.9	107.5
Au_{42}-SeC_6H_4F	2.60	2.63	1.96	67.3	89.4	107.1
Au_{42}-SeC_6H_4Cl	2.61	2.64	1.96	67.0	89.6	106.7
Au_{42}-$SeC_6H_4OCOCH_3$	2.60	2.70	1.95	66.3	89.0	106.7
Au_{42}-$SeC_6H_4CF_3$	2.61	2.63	1.96	67.1	89.0	107.0
Au_{42}-SeC_6H_4CN	2.60	2.66	1.96	66.8	90.5	108.0
Au_{42}-$SeC_6H_4NO_2$	2.62	2.64	1.95	66.8	89.0	106.1

[a] The nomenclature used on the column headings for the definition of the geometric parameters is detailed on Figure 2 from the manuscript.

Table S3. Selected geometric parameters for the anionic *meta* substituted telurophenolates-gold complexes[a] (distances in Å and angles in deg).

**Au_{42}-Telurophenolate (Te^-)**	**D_1**	**D_2**	**D_{C-S}**	**α**	**β**	**γ**
Au_{42}-$TeC_6H_4NH_2$	2.69	2.73	2.17	64.6	84.1	98.8
Au_{42}-$TeC_6H_4OCH_3$	2.70	2.72	2.17	64.7	88.4	103.4
Au_{42}-$TeC_6H_4CH_3$	2.69	2.72	2.17	64.7	87.9	103.4
Au_{42}-TeC_6H_5	2.69	2.70	2.17	64.9	86.3	102.5
Au_{42}-TeC_6H_4F	2.70	2.71	2.17	64.7	86.9	102.9
Au_{42}-TeC_6H_4Cl	2.70	2.71	2.18	64.7	87.5	103.5
Au_{42}-$TeC_6H_4OCOCH_3$	2.70	2.71	2.18	64.7	87.7	103.6
Au_{42}-$TeC_6H_4CF_3$	2.69	2.71	2.18	64.9	84.1	100.6
Au_{42}-TeC_6H_4CN	2.70	2.70	2.18	64.8	85.8	102.1
Au_{42}-$TeC_6H_4NO_2$	2.69	2.72	2.18	64.8	84.3	100.7

[a] The nomenclature used on the column headings for the definition of the geometric parameters is detailed on Figure 2 from the manuscript.

Table S4. Selected geometric parameters for the anionic *ortho* substituted thiophenolate-gold complexes[a] (distances in Å and angles in deg).

**Au_{42}-Thiophenolate (S^-)**	**D_1**	**D_2**	**$D_{C\text{-}S}$**	**α**	**β**	**γ**
Au_{42}-$SC_6H_4NH_2$	2.60	2.64	1.78	67.1	91.2	107.1
Au_{42}-$SC_6H_4OCH_3$	2.54	2.59	1.79	68.8	90.6	109.9
Au_{42}-$SC_6H_4CH_3$	2.52	2.67	1.79	67.8	88.6	108.9
Au_{42}-SC_6H_5	2.52	2.60	1.80	69.0	91.4	110.1
Au_{42}-SC_6H_4F	2.52	2.67	1.78	67.7	89.2	108.8
Au_{42}-SC_6H_4Cl	2.56	2.60	1.79	68.3	92.4	113.0
Au_{42}-$SC_6H_4OCOCH_3$	2.56	2.60	1.78	68.2	90.6	111.6
Au_{42}-$SC_6H_4CF_3$	2.50	2.92	1.78	64.1	91.3	113.7
Au_{42}-SC_6H_4CN	2.53	2.84	1.76	65.0	87.1	106.6
Au_{42}-$SC_6H_4NO_2$	2.54	2.65	1.77	67.8	87.8	110.2

[a] The nomenclature used on the column headings for the definition of the geometric parameters is detailed on Figure 2 from the manuscript.

Table S5. Selected geometric parameters for the anionic *ortho* substituted selenophenolate-gold complexes[a] (distances in Å and angles in deg).

**Au_{42}-Selenophenolate (Se^-)**	**D_1**	**D_2**	**$D_{C\text{-}S}$**	**α**	**β**	**γ**
Au_{42}-$SeC_6H_4NH_2$	2.62	2.67	1.95	66.4	91.2	107.5
Au_{42}-$SeC_6H_4OCH_3$	2.61	2.61	1.96	67.4	88.7	106.6
Au_{42}-$SeC_6H_4CH_3$	2.61	2.65	1.96	66.8	87.7	106.9
Au_{42}-SeC_6H_5	2.60	2.63	1.96	67.3	90.5	107.5
Au_{42}-SeC_6H_4F	2.61	2.65	1.95	66.8	89.3	107.5
Au_{42}-SeC_6H_4Cl	2.61	2.65	1.95	66.8	88.4	107.3
Au_{42}-$SeC_6H_4OCOCH_3$	2.63	2.63	1.95	66.8	90.6	109.6
Au_{42}-$SeC_6H_4CF_3$	2.59	2.72	1.97	66.1	94.1	115.4
Au_{42}-SeC_6H_4CN	2.61	2.69	1.95	66.3	87.0	106.3
Au_{42}-$SeC_6H_4NO_2$	2.61	2.62	1.95	67.2	86.3	106.3

[a] The nomenclature used on the column headings for the definition of the geometric parameters is detailed on Figure 2 from the manuscript.

Table S6. Selected geometric parameters for the anionic *ortho* substituted telurophenolates-gold complexes[a] (distances in Å and angles in deg).

Au_{42}-Telurophenolate (Te^-)	D_1	D_2	D_{C-S}	α	β	γ
Au_{42}-$TeC_6H_4NH_2$	2.71	2.74	2.17	64.2	89.9	104.3
Au_{42}-$TeC_6H_4OCH_3$	2.69	2.72	2.17	64.8	86.9	103.2
Au_{42}-$TeC_6H_4CH_3$	2.70	2.72	2.18	64.5	85.1	102.4
Au_{42}-TeC_6H_5	2.69	2.70	2.17	64.9	86.3	102.5
Au_{42}-TeC_6H_4F	2.70	2.72	2.17	64.6	87.5	104.5
Au_{42}-TeC_6H_4Cl	2.70	2.71	2.18	64.7	85.7	102.8
Au_{42}-$TeC_6H_4OCOCH_3$	2.70	2.73	2.17	64.5	90.5	107.1
Au_{42}-$TeC_6H_4CF_3$	2.73	2.70	2.20	64.5	89.3	108.7
Au_{42}-TeC_6H_4CN	2.71	2.71	2.18	64.7	81.8	99.4
Au_{42}-$TeC_6H_4NO_2$	2.69	2.71	2.19	65.0	82.2	100.1

[a] The nomenclature used on the column headings for the definition of the geometric parameters is detailed on Figure 2 from the manuscript.

Table S7. Selected geometric parameters for the anionic multi-substituted selenophenolate-gold complexes[a] (distances in Å and angles in deg).

**Au_{42}-Selenophenolate (Se^-)**	**D_1**	**D_2**	**D_{C-Se}**	**α**	**β**	γ
(NH_2)m-m	2.63	2.75	1.95	65.1	88.0	105.3
(NH_2)m-o	2.71	2.77	1.95	63.8	92.7	107.0
(NH_2)m-p	2.63	2.65	1.95	66.5	87.7	107.7
(NH_2)o-o	2.69	2.78	1.95	63.9	95.8	111.9
(NH_2)o-p	2.66	2.72	1.95	65.1	94.7	111.2
(NH_2)m-m-o	2.68	2.75	1.95	64.4	87.2	104.4
(NH_2)m-m-p	2.64	2.71	1.95	65.6	86.3	103.9
(NH_2)m-o-o	2.73	2.81	1.94	63.1	90.8	106.6
(NH_2)o-o-p	2.64	2.85	1.93	63.5	91.7	109.0

Table S8. Energy decomposition analysis of the interaction energies calculated for the multisubstituted ligands. The data is presented as percentages of contribution to the stabilizing covalent component.

**Model System**	**% Orb**	**% Elect**
(NH_2)m-m-p	35.5	64.5
(NH_2)o-o-p	36.5	63.5
(NH_2)m-o-o	34.5	65.5
(NH_2)m-m-o	34.8	65.2
(NH_2)m-m	34.9	65.1
(NH_2)o-p	34.7	65.3
(NH_2)m-p	39.1	60.9
(NH_2)o-o	34.8	65.2
(NH_2)m-o	35.9	64.1

Table S9. Quantification of the charge transfer process between the multi-substituted ligands and the gold-based substrate. These were obtained from the difference between the NPA charges of the complexes and the free fragments.

	Se	
**Model**	**Au_{42}**	**ΔSe^{a}**
(NH_2)m-m-p	-0.70	0.42
(NH_2)o-o-p	-0.76	0.44
(NH_2)m-o-o	-0.71	0.37
(NH_2)m-m-o	-0.68	0.38
(NH_2)m-m	-0.66	0.41
(NH_2)o-p	-0.70	0.45
(NH_2)m-p	-0.69	0.47
(NH_2)o-o	-0.68	0.41
(NH_2)m-o	-0.65	0.41

[a] Values represent the amount of charge donated directly by the chalcogen atom and it was obtained from the difference between the chalcogen charge in the complex and the free ligand.

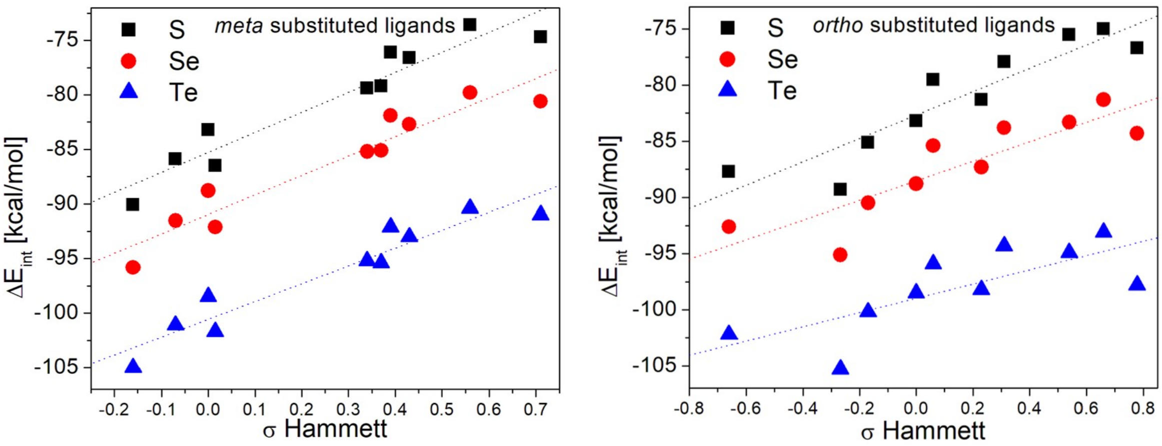

Figure S1. Plot including the complete series of the interaction energies of the phenyl-chalcogenolates functionalized in *meta* (left) and *ortho* (right) positions versus the Hammett sigma constants σ_{meta} and σ_{ortho}.

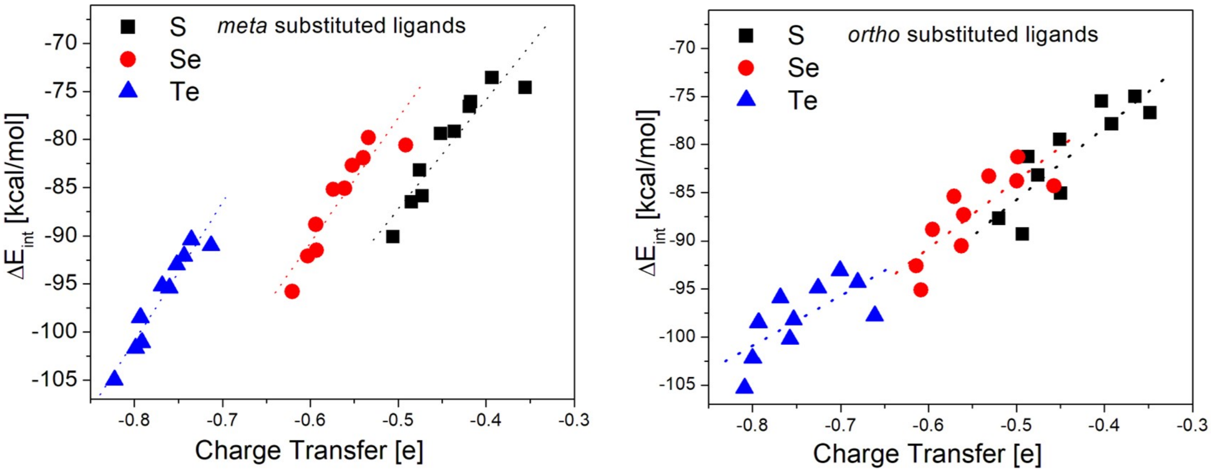

Figure S2. Plot including the complete series of the amount of charge transferred to the gold substrate from the phenyl-chalcogenolates functionalized in *meta* (left) and *ortho* (right) positions.

Coordinates from the Sulfur-based Ligands

META

s-m-nh2

S	-0.7605436	0.1012865	-3.1607091
C	0.9017681	0.5795325	-3.6334441
C	1.8715701	-0.4218389	-3.8638649
C	1.2532847	1.9285309	-3.7642419
C	3.1795406	-0.0431653	-4.1735849
H	1.5876664	-1.4667022	-3.7975182
C	2.5766156	2.3034764	-4.0730545
C	3.5466989	1.3003935	-4.2531616
H	3.9324794	-0.8113790	-4.3260616
H	4.5741382	1.5778452	-4.4727552
H	0.4969431	2.6935424	-3.6076240
N	2.8936355	3.6491616	-4.2124812
H	2.2628191	4.2795694	-3.7212820
H	3.8617963	3.8780750	-4.0108302
Au	-4.4117667	-5.2868588	1.3070186
Au	-1.5183685	-5.2670588	1.3222186
Au	-2.9540671	-2.7800605	1.3118186
Au	-0.0861689	-2.7894605	1.3070186
Au	2.8072293	-2.7696605	1.3222186
Au	1.3715307	-0.2827622	1.3118186
Au	-4.4117667	-0.2921622	1.3070186
Au	-1.5183685	-0.2723622	1.3222186
Au	-2.9540671	2.2146371	1.3118186
Au	-0.0861689	2.2052371	1.3070186

Au	2.8072293	2.2250371	1.3222186
Au	1.3715307	-5.2774588	1.3118186
Au	4.2606289	-0.2802622	1.2668186
Au	-5.8543653	-2.7756605	1.3110186
Au	-4.4289667	-3.6872602	3.5957180
Au	-1.5354685	-3.7019602	3.5963180
Au	-2.9415671	-1.1988609	3.6597169
Au	-0.0739689	-1.2418609	3.6397179
Au	2.8194293	-1.2565609	3.6404179
Au	1.4134307	1.2464374	3.7037169
Au	-4.3694667	1.3053374	3.7287169
Au	-1.4760685	1.2906374	3.7294169
Au	-2.8820671	3.7937357	3.7927169
Au	-0.0144689	3.7507357	3.7728169
Au	2.8789292	3.7360357	3.7734169
Au	1.3539307	-3.7460602	3.5706180
Au	4.3021288	1.2161374	3.6438169
Au	-5.8415653	-1.1602609	3.6741169
Au	2.7635293	3.8821357	-0.9514798
Au	-0.1291689	3.8277357	-0.9172798
Au	1.3352307	1.3589374	-1.0003798
Au	-1.5325685	1.3346374	-0.9855798
Au	-4.4253667	1.2803374	-0.9512798
Au	-2.9609671	-1.1883609	-1.0344797
Au	2.8216293	-1.1106609	-1.0781797
Au	-0.0711689	-1.1649609	-1.0438797
Au	1.3932307	-3.6337602	-1.1270797

Au	-1.4745685	-3.6581602	-1.1122797
Au	-4.3672667	-3.7124602	-1.0779797
Au	-3.0190671	3.8043357	-0.9078798
Au	-5.8500653	-1.2238609	-1.0599797
Au	4.2352289	1.3888374	-1.0209797

s-m-och3

S	-0.5636987	-0.2396894	-2.9616881
C	0.8077818	0.7987156	-3.4805672
C	2.0911884	0.2581181	-3.6720227
C	0.5673381	2.1573809	-3.7551820
C	3.1349880	1.0996081	-4.0781100
H	2.2571178	-0.8013893	-3.5014191
C	1.6267854	2.9818774	-4.1343849
C	2.9185042	2.4526375	-4.2958326
H	4.1328857	0.6874475	-4.1970493
H	3.7280837	3.1203026	-4.5701654
H	-0.4330538	2.5454395	-3.6021139
C	0.2510276	4.9288890	-3.9993782
O	1.5054618	4.3276849	-4.3427129
H	0.3907087	6.0008591	-4.1417814
H	-0.0019737	4.7197793	-2.9457156
H	-0.5566117	4.5640073	-4.6468565
Au	-4.1767806	-5.7670950	1.4801705
Au	-1.2833824	-5.7472950	1.4953705
Au	-2.7190810	-3.2602967	1.4849705
Au	0.1488172	-3.2696967	1.4801705

Au	3.0422153	-3.2498967	1.4953705
Au	1.6065167	-0.7629984	1.4849705
Au	-4.1767806	-0.7723984	1.4801705
Au	-1.2833824	-0.7525984	1.4953705
Au	-2.7190810	1.7344009	1.4849705
Au	0.1488172	1.7250009	1.4801705
Au	3.0422153	1.7448009	1.4953705
Au	1.6065167	-5.7576950	1.4849705
Au	4.4956149	-0.7604984	1.4399705
Au	-5.6193792	-3.2558967	1.4841705
Au	-4.1939806	-4.1674964	3.7688699
Au	-1.3004824	-4.1821964	3.7694699
Au	-2.7065810	-1.6790971	3.8328689
Au	0.1610171	-1.7220971	3.8128699
Au	3.0544153	-1.7367971	3.8135699
Au	1.6484167	0.7662012	3.8768689
Au	-4.1344806	0.8251012	3.9018689
Au	-1.2410825	0.8104012	3.9025689
Au	-2.6470811	3.3134995	3.9658688
Au	0.2205171	3.2704995	3.9459688
Au	3.1139153	3.2557995	3.9465688
Au	1.5889167	-4.2262964	3.7437699
Au	4.5371149	0.7359012	3.8169689
Au	-5.6065792	-1.6404971	3.8472689
Au	2.9985154	3.4018995	-0.7783278
Au	0.1058172	3.3474995	-0.7441278
Au	1.5702168	0.8787012	-0.8272278

Au	-1.2975824	0.8544012	-0.8124278
Au	-4.1903806	0.8001012	-0.7781278
Au	-2.7259810	-1.6685971	-0.8613278
Au	3.0566153	-1.5908971	-0.9050278
Au	0.1638171	-1.6451971	-0.8707278
Au	1.6282167	-4.1139964	-0.9539278
Au	-1.2395825	-4.1383964	-0.9391278
Au	-4.1322806	-4.1926964	-0.9048278
Au	-2.7840810	3.3240995	-0.7347278
Au	-5.6150792	-1.7040971	-0.8868278
Au	4.4702149	0.9086012	-0.8478278

s-m-ch3

S	-0.6572421	-0.1744713	-3.0069306
C	0.7234549	0.8461654	-3.5358839
C	2.0037051	0.2935325	-3.7361166
C	0.5121631	2.2092549	-3.7945845
C	3.0553516	1.1279251	-4.1226826
H	2.1552293	-0.7712478	-3.5824877
C	1.5645186	3.0472572	-4.1683236
C	2.8435819	2.4926197	-4.3163226
H	4.0494019	0.7076161	-4.2472705
H	3.6794294	3.1357475	-4.5782170
H	-0.4843732	2.6173255	-3.6419215
C	1.3285700	4.5299375	-4.2873146
H	0.3723453	4.7454981	-4.7748773
H	1.2922072	4.9733070	-3.2807160

H	2.1292451	5.0258038	-4.8441559
Au	-4.2476099	-5.6585513	1.4258794
Au	-1.3542117	-5.6387513	1.4410794
Au	-2.7899103	-3.1517530	1.4306794
Au	0.0779879	-3.1611530	1.4258794
Au	2.9713861	-3.1413530	1.4410794
Au	1.5356875	-0.6544547	1.4306794
Au	-4.2476099	-0.6638547	1.4258794
Au	-1.3542117	-0.6440547	1.4410794
Au	-2.7899103	1.8429446	1.4306794
Au	0.0779879	1.8335446	1.4258794
Au	2.9713861	1.8533446	1.4410794
Au	1.5356875	-5.6491513	1.4306794
Au	4.4247857	-0.6519547	1.3856794
Au	-5.6902085	-3.1473530	1.4298794
Au	-4.2648099	-4.0589528	3.7145787
Au	-1.3713117	-4.0736528	3.7151787
Au	-2.7774103	-1.5705535	3.7785777
Au	0.0901879	-1.6135535	3.7585787
Au	2.9835861	-1.6282535	3.7592787
Au	1.5775875	0.8747448	3.8225777
Au	-4.2053099	0.9336448	3.8475777
Au	-1.3119117	0.9189448	3.8482777
Au	-2.7179103	3.4220431	3.9115777
Au	0.1496879	3.3790431	3.8916777
Au	3.0430861	3.3643431	3.8922777
Au	1.5180875	-4.1177528	3.6894788

Au	4.4662857	0.8444449	3.7626777
Au	-5.6774085	-1.5319535	3.7929777
Au	2.9276861	3.5104431	-0.8326190
Au	0.0349879	3.4560431	-0.7984190
Au	1.4993875	0.9872448	-0.8815190
Au	-1.3684117	0.9629448	-0.8667190
Au	-4.2612099	0.9086448	-0.8324190
Au	-2.7968103	-1.5600535	-0.9156190
Au	2.9857861	-1.4823535	-0.9593189
Au	0.0929879	-1.5366535	-0.9250189
Au	1.5573875	-4.0054528	-1.0082189
Au	-1.3104117	-4.0298528	-0.9934189
Au	-4.2031099	-4.0841528	-0.9591189
Au	-2.8549103	3.4326431	-0.7890190
Au	-5.6859085	-1.5955535	-0.9411189
Au	4.3993857	1.0171448	-0.9021190

s-m-h

S	-0.5219586	0.1930633	-3.1847429
C	0.9261716	1.1015253	-3.7391009
C	2.1321262	0.4176103	-3.9869070
C	0.8433533	2.4766805	-4.0180619
C	3.2518791	1.1192221	-4.4345804
H	2.1773325	-0.6534366	-3.8082237
C	1.9686154	3.1657989	-4.4607235
H	-0.0932230	2.9954894	-3.8319646
C	3.1784502	2.4947906	-4.6568404

H	4.1854516	0.5867643	-4.5917845
H	1.9039676	4.2364375	-4.6318278
H	4.0596122	3.0419981	-4.9778534
Au	-4.1330848	-5.3128399	1.2404040
Au	-1.2396864	-5.2930400	1.2556040
Au	-2.6753856	-2.8060414	1.2452040
Au	0.1925128	-2.8154413	1.2404040
Au	3.0859112	-2.7956414	1.2556040
Au	1.6502120	-0.3087428	1.2452040
Au	-4.1330848	-0.3181428	1.2404040
Au	-1.2396864	-0.2983428	1.2556040
Au	-2.6753856	2.1886558	1.2452040
Au	0.1925128	2.1792558	1.2404040
Au	3.0859112	2.1990558	1.2556040
Au	1.6502120	-5.3034400	1.2452040
Au	4.5393104	-0.3062428	1.2002041
Au	-5.5756840	-2.8016414	1.2444040
Au	-4.1502848	-3.7132409	3.5291027
Au	-1.2567864	-3.7279409	3.5297027
Au	-2.6628856	-1.2248423	3.5931027
Au	0.2047128	-1.2678423	3.5731027
Au	3.0981112	-1.2825423	3.5738027
Au	1.6921120	1.2204563	3.6371027
Au	-4.0907848	1.2793563	3.6621027
Au	-1.1973864	1.2646563	3.6628027
Au	-2.6033856	3.7677549	3.7261027
Au	0.2642128	3.7247549	3.7062027

Au	3.1576112	3.7100549	3.7068027
Au	1.6326120	-3.7720409	3.5040027
Au	4.5808104	1.1901563	3.5772027
Au	-5.5628840	-1.1862423	3.6075027
Au	3.0422112	3.8561549	-1.0180947
Au	0.1495128	3.8017549	-0.9838947
Au	1.6139120	1.3329563	-1.0669947
Au	-1.2538864	1.3086563	-1.0521947
Au	-4.1466848	1.2543563	-1.0178947
Au	-2.6822856	-1.2143423	-1.1010947
Au	3.1003112	-1.1366423	-1.1447947
Au	0.2075128	-1.1909423	-1.1104947
Au	1.6719120	-3.6597409	-1.1936947
Au	-1.1958864	-3.6841409	-1.1788947
Au	-4.0885848	-3.7384409	-1.1445947
Au	-2.7403856	3.7783549	-0.9744947
Au	-5.5713840	-1.2498423	-1.1265947
Au	4.5139104	1.3628563	-1.0875947

s-m-f

S	-0.6119490	0.1422281	-3.2520089
C	0.7637757	1.1493510	-3.7983343
C	2.0493418	0.5930101	-3.9576625
C	0.5527036	2.4996634	-4.1251458
C	3.1160054	1.3986757	-4.3632978
H	2.1973257	-0.4631365	-3.7513940
C	1.6348637	3.2586393	-4.5328048

C	2.9257031	2.7506609	-4.6426814
H	4.1082104	0.9649178	-4.4439152
H	3.7426877	3.4002644	-4.9344465
H	-0.4252731	2.9543049	-4.0079506
F	1.4292793	4.5748579	-4.8114313
Au	-4.1689285	-5.3719330	1.1991253
Au	-1.2755304	-5.3521330	1.2143253
Au	-2.7112290	-2.8651347	1.2039253
Au	0.1566692	-2.8745347	1.1991253
Au	3.0500674	-2.8547347	1.2143253
Au	1.6143688	-0.3678364	1.2039253
Au	-4.1689285	-0.3772364	1.1991253
Au	-1.2755304	-0.3574364	1.2143253
Au	-2.7112290	2.1295629	1.2039253
Au	0.1566692	2.1201629	1.1991253
Au	3.0500674	2.1399629	1.2143253
Au	1.6143688	-5.3625330	1.2039253
Au	4.5034670	-0.3653364	1.1589253
Au	-5.6115271	-2.8607347	1.2031253
Au	-4.1861285	-3.7723344	3.4878246
Au	-1.2926304	-3.7870344	3.4884246
Au	-2.6987290	-1.2839351	3.5518236
Au	0.1688692	-1.3269351	3.5318246
Au	3.0622674	-1.3416351	3.5325246
Au	1.6562688	1.1613632	3.5958236
Au	-4.1266286	1.2202632	3.6208236
Au	-1.2332304	1.2055632	3.6215236

Au	-2.6392290	3.7086615	3.6848236
Au	0.2283692	3.6656615	3.6649236
Au	3.1217674	3.6509615	3.6655236
Au	1.5967688	-3.8311344	3.4627246
Au	4.5449670	1.1310632	3.5359236
Au	-5.5987271	-1.2453352	3.5662236
Au	3.0063674	3.7970614	-1.0593731
Au	0.1136692	3.7426614	-1.0251731
Au	1.5780688	1.2738631	-1.1082731
Au	-1.2897304	1.2495631	-1.0934731
Au	-4.1825285	1.1952632	-1.0591731
Au	-2.7181290	-1.2734351	-1.1423731
Au	3.0644674	-1.1957352	-1.1860730
Au	0.1716692	-1.2500352	-1.1517731
Au	1.6360688	-3.7188345	-1.2349730
Au	-1.2317304	-3.7432345	-1.2201730
Au	-4.1244286	-3.7975344	-1.1858730
Au	-2.7762289	3.7192615	-1.0157731
Au	-5.6072271	-1.3089351	-1.1678731
Au	4.4780670	1.3037631	-1.1288731

s-m-cl

S	-0.6386960	0.1327476	-3.2870365
C	0.7417027	1.1372321	-3.8202026
C	2.0160279	0.5703726	-4.0262315
C	0.5559474	2.5120810	-4.0409578
C	3.0899457	1.3858261	-4.3876343

H	2.1515662	-0.4973539	-3.8815430
C	1.6485927	3.2994974	-4.3809617
C	2.9252282	2.7609261	-4.5483692
H	4.0755502	0.9462420	-4.5094218
H	3.7648708	3.4048270	-4.7816192
H	-0.4196224	2.9586679	-3.8795512
Cl	1.4233859	5.0304408	-4.5049614
Au	-4.1708603	-5.3835566	1.1860146
Au	-1.2774621	-5.3637566	1.2012146
Au	-2.7131607	-2.8767583	1.1908146
Au	0.1547375	-2.8861583	1.1860146
Au	3.0481357	-2.8663583	1.2012146
Au	1.6124371	-0.3794600	1.1908146
Au	-4.1708603	-0.3888600	1.1860146
Au	-1.2774621	-0.3690600	1.2012146
Au	-2.7131607	2.1179393	1.1908146
Au	0.1547375	2.1085393	1.1860146
Au	3.0481357	2.1283393	1.2012146
Au	1.6124371	-5.3741566	1.1908146
Au	4.5015353	-0.3769600	1.1458146
Au	-5.6134589	-2.8723583	1.1900146
Au	-4.1880603	-3.7839580	3.4747139
Au	-1.2945621	-3.7986580	3.4753139
Au	-2.7006607	-1.2955587	3.5387129
Au	0.1669375	-1.3385587	3.5187139
Au	3.0603357	-1.3532587	3.5194139
Au	1.6543371	1.1497396	3.5827129

Au	-4.1285603	1.2086396	3.6077129
Au	-1.2351621	1.1939396	3.6084129
Au	-2.6411607	3.6970379	3.6717129
Au	0.2264375	3.6540379	3.6518129
Au	3.1198357	3.6393379	3.6524129
Au	1.5948371	-3.8427580	3.4496139
Au	4.5430353	1.1194396	3.5228129
Au	-5.6006589	-1.2569587	3.5531129
Au	3.0044357	3.7854379	-1.0724838
Au	0.1117375	3.7310379	-1.0382838
Au	1.5761371	1.2622396	-1.1213838
Au	-1.2916621	1.2379396	-1.1065838
Au	-4.1844603	1.1836396	-1.0722838
Au	-2.7200607	-1.2850587	-1.1554838
Au	3.0625357	-1.2073587	-1.1991838
Au	0.1697375	-1.2616587	-1.1648838
Au	1.6341371	-3.7304580	-1.2480837
Au	-1.2336621	-3.7548580	-1.2332837
Au	-4.1263603	-3.8091580	-1.1989838
Au	-2.7781607	3.7076379	-1.0288838
Au	-5.6091589	-1.3205587	-1.1809838
Au	4.4761353	1.2921396	-1.1419838

s-m-ococh3

S	-0.6885809	-0.2675327	-2.6285471
C	0.6344727	0.8123561	-3.1510600
C	1.9459922	0.3221030	-3.3142509

C	0.3675045	2.1628226	-3.4313744
C	2.9732805	1.1897366	-3.6960465
H	2.1440171	-0.7289169	-3.1280945
C	1.4014461	2.9976445	-3.8309916
C	2.7148714	2.5361531	-3.9443576
H	3.9875975	0.8102413	-3.7758226
H	3.5062096	3.2278917	-4.2114719
H	-0.6328101	2.5599432	-3.2872013
C	0.5914347	4.8364990	-5.1874646
O	1.1548167	4.3646802	-4.0142926
O	0.3823718	6.0202892	-5.2826553
C	0.2959367	3.8127797	-6.2602553
H	-0.0065464	4.3419523	-7.1634118
H	1.1731524	3.1881089	-6.4558461
H	-0.5079592	3.1447105	-5.9360594
Au	-4.1766404	-5.8429033	1.8577168
Au	-1.2832422	-5.8231033	1.8729168
Au	-2.7189408	-3.3361050	1.8625168
Au	0.1489574	-3.3455050	1.8577168
Au	3.0423555	-3.3257050	1.8729168
Au	1.6066569	-0.8388067	1.8625168
Au	-4.1766404	-0.8482067	1.8577168
Au	-1.2832422	-0.8284068	1.8729168
Au	-2.7189408	1.6585926	1.8625168
Au	0.1489574	1.6491926	1.8577168
Au	3.0423555	1.6689925	1.8729168
Au	1.6066569	-5.8335033	1.8625168

Au	4.4957551	-0.8363067	1.8175168
Au	-5.6192390	-3.3317050	1.8617168
Au	-4.1938404	-4.2433048	4.1464162
Au	-1.3003422	-4.2580048	4.1470162
Au	-2.7064408	-1.7549055	4.2104152
Au	0.1611574	-1.7979055	4.1904162
Au	3.0545555	-1.8126055	4.1911162
Au	1.6485569	0.6903928	4.2544151
Au	-4.1343404	0.7492928	4.2794151
Au	-1.2409423	0.7345928	4.2801151
Au	-2.6469409	3.2376911	4.3434151
Au	0.2206573	3.1946911	4.3235151
Au	3.1140555	3.1799911	4.3241151
Au	1.5890570	-4.3021048	4.1213162
Au	4.5372551	0.6600928	4.1945152
Au	-5.6064390	-1.7163055	4.2248151
Au	2.9986556	3.3260911	-0.4007816
Au	0.1059574	3.2716911	-0.3665816
Au	1.5703570	0.8028928	-0.4496815
Au	-1.2974422	0.7785928	-0.4348815
Au	-4.1902404	0.7242928	-0.4005816
Au	-2.7258408	-1.7444055	-0.4837815
Au	3.0567555	-1.6667055	-0.5274815
Au	0.1639574	-1.7210055	-0.4931815
Au	1.6283569	-4.1898048	-0.5763815
Au	-1.2394423	-4.2142048	-0.5615815
Au	-4.1321404	-4.2685048	-0.5272815

Au	-2.7839408	3.2482911	-0.3571816
Au	-5.6149390	-1.7799055	-0.5092815
Au	4.4703551	0.8327928	-0.4702815

s-m-cf3

S	-0.7086957	-0.1153058	-3.0187004
C	0.6764218	0.8695091	-3.5662092
C	1.9494914	0.2922270	-3.7360072
C	0.5000408	2.2309103	-3.8626355
C	3.0316281	1.0770736	-4.1402768
H	2.0766384	-0.7675393	-3.5344412
C	1.5877149	2.9974516	-4.2655493
C	2.8623181	2.4339913	-4.3957343
H	4.0143906	0.6244487	-4.2303718
Au	-4.2138633	-5.6289344	1.4500993
Au	-1.3204651	-5.6091344	1.4652993
Au	-2.7561637	-3.1221361	1.4548993
Au	0.1117345	-3.1315361	1.4500993
Au	3.0051326	-3.1117361	1.4652993
Au	1.5694341	-0.6248378	1.4548993
Au	-4.2138633	-0.6342378	1.4500993
Au	-1.3204651	-0.6144378	1.4652993
Au	-2.7561637	1.8725615	1.4548993
Au	0.1117345	1.8631615	1.4500993
Au	3.0051326	1.8829615	1.4652993
Au	1.5694341	-5.6195344	1.4548993
Au	4.4585322	-0.6223378	1.4098993

Au	-5.6564619	-3.1177361	1.4540993
Au	-4.2310633	-4.0293358	3.7387987
Au	-1.3375651	-4.0440358	3.7393987
Au	-2.7436637	-1.5409365	3.8027977
Au	0.1239345	-1.5839365	3.7827987
Au	3.0173326	-1.5986365	3.7834987
Au	1.6113340	0.9043618	3.8467977
Au	-4.1715633	0.9632618	3.8717976
Au	-1.2781651	0.9485618	3.8724976
Au	-2.6841638	3.4516601	3.9357976
Au	0.1834344	3.4086601	3.9158976
Au	3.0768326	3.3939601	3.9164976
Au	1.5518341	-4.0881358	3.7136987
Au	4.5000322	0.8740618	3.7868977
Au	-5.6436619	-1.5023365	3.8171977
Au	2.9614327	3.5400601	-0.8083990
Au	0.0687345	3.4856601	-0.7741990
Au	1.5331341	1.0168618	-0.8572990
Au	-1.3346651	0.9925618	-0.8424990
Au	-4.2274633	0.9382618	-0.8081990
Au	-2.7630637	-1.5304365	-0.8913990
Au	3.0195326	-1.4527365	-0.9350990
Au	0.1267345	-1.5070365	-0.9007990
Au	1.5911340	-3.9758358	-0.9839990
Au	-1.2766652	-4.0002358	-0.9691990
Au	-4.1693633	-4.0545358	-0.9348990
Au	-2.8211637	3.4622601	-0.7647990

Au	-5.6521619	-1.5659365	-0.9168990
Au	4.4331322	1.0467618	-0.8778990
H	3.7053304	3.0550243	-4.6770355
H	-0.4767008	2.6831765	-3.7272656
C	1.3939462	4.4566737	-4.5690587
F	1.2156745	4.6800535	-5.9096520
F	0.3098005	4.9848226	-3.9456971
F	2.4702847	5.2051073	-4.2018571

s-m-cn

S	-0.7186110	0.1335828	-3.2541483
C	0.6538326	1.1519671	-3.7545239
C	1.9437452	0.5979447	-3.8923768
C	0.4677198	2.5145065	-4.0349456
C	3.0261435	1.4015524	-4.2644299
H	2.0793508	-0.4637170	-3.7092449
C	1.5578637	3.3104302	-4.4078184
C	2.8493978	2.7571244	-4.5110405
H	4.0157201	0.9606610	-4.3371101
H	3.6879575	3.3911463	-4.7758862
H	-0.5190873	2.9533621	-3.9297845
C	1.3617993	4.7072712	-4.6412157
N	1.2061356	5.8450891	-4.8250186
Au	-4.1346050	-5.5097663	1.2770956
Au	-1.2412068	-5.4899663	1.2922956
Au	-2.6769054	-3.0029680	1.2818956
Au	0.1909928	-3.0123680	1.2770956

Au	3.0843910	-2.9925681	1.2922956
Au	1.6486924	-0.5056697	1.2818956
Au	-4.1346050	-0.5150697	1.2770956
Au	-1.2412068	-0.4952698	1.2922956
Au	-2.6769054	1.9917295	1.2818956
Au	0.1909928	1.9823296	1.2770956
Au	3.0843910	2.0021295	1.2922956
Au	1.6486924	-5.5003663	1.2818956
Au	4.5377906	-0.5031697	1.2368956
Au	-5.5772036	-2.9985680	1.2810956
Au	-4.1518050	-3.9101678	3.5657950
Au	-1.2583068	-3.9248678	3.5663950
Au	-2.6644054	-1.4217685	3.6297940
Au	0.2031928	-1.4647685	3.6097950
Au	3.0965910	-1.4794685	3.6104950
Au	1.6905924	1.0235298	3.6737940
Au	-4.0923050	1.0824298	3.6987940
Au	-1.1989068	1.0677298	3.6994940
Au	-2.6049054	3.5708281	3.7627939
Au	0.2626928	3.5278281	3.7428939
Au	3.1560910	3.5131281	3.7434939
Au	1.6310924	-3.9689678	3.5406950
Au	4.5792906	0.9932298	3.6138940
Au	-5.5644036	-1.3831685	3.6441940
Au	3.0406910	3.6592281	-0.9814027
Au	0.1479928	3.6048281	-0.9472027
Au	1.6123924	1.1360298	-1.0303027

Au	-1.2554068	1.1117298	-1.0155027
Au	-4.1482050	1.0574298	-0.9812027
Au	-2.6838054	-1.4112685	-1.0644027
Au	3.0987910	-1.3335685	-1.1081027
Au	0.2059928	-1.3878685	-1.0738027
Au	1.6703924	-3.8566678	-1.1570027
Au	-1.1974068	-3.8810678	-1.1422027
Au	-4.0901050	-3.9353678	-1.1079027
Au	-2.7419054	3.5814281	-0.9378027
Au	-5.5729036	-1.4467685	-1.0899027
Au	4.5123906	1.1659298	-1.0509027

s-m-no2

S	-0.6382589	-0.1412248	-3.1426285
C	0.7631724	0.8161015	-3.6924414
C	2.0033786	0.2040220	-3.9726021
C	0.6438508	2.2069458	-3.8366801
C	3.1047779	0.9766897	-4.3509879
H	2.0932155	-0.8727146	-3.8637954
C	1.7629583	2.9472909	-4.1959952
C	3.0039916	2.3616468	-4.4548780
H	4.0597247	0.4909809	-4.5262267
H	3.8539267	2.9865719	-4.6975280
H	-0.2955309	2.7036884	-3.6232264
N	1.6510069	4.4127391	-4.1813769
O	0.6168878	4.9047490	-3.7042996
O	2.6107468	5.0734551	-4.5968245

Au	-4.1955769	-5.5524203	1.3137822
Au	-1.3021787	-5.5326203	1.3289822
Au	-2.7378773	-3.0456220	1.3185822
Au	0.1300209	-3.0550220	1.3137822
Au	3.0234191	-3.0352220	1.3289822
Au	1.5877205	-0.5483237	1.3185822
Au	-4.1955769	-0.5577237	1.3137822
Au	-1.3021787	-0.5379237	1.3289822
Au	-2.7378773	1.9490756	1.3185822
Au	0.1300209	1.9396756	1.3137822
Au	3.0234191	1.9594756	1.3289822
Au	1.5877205	-5.5430203	1.3185822
Au	4.4768187	-0.5458237	1.2735822
Au	-5.6381755	-3.0412220	1.3177822
Au	-4.2127769	-3.9528217	3.6024816
Au	-1.3192787	-3.9675217	3.6030816
Au	-2.7253773	-1.4644224	3.6664806
Au	0.1422209	-1.5074224	3.6464816
Au	3.0356191	-1.5221224	3.6471816
Au	1.6296205	0.9808759	3.7104805
Au	-4.1532769	1.0397759	3.7354805
Au	-1.2598787	1.0250759	3.7361805
Au	-2.6658773	3.5281742	3.7994805
Au	0.2017209	3.4851742	3.7795805
Au	3.0951190	3.4704742	3.7801805
Au	1.5701205	-4.0116217	3.5773816
Au	4.5183186	0.9505759	3.6505806

Au	-5.6253755	-1.4258224	3.6808806
Au	2.9797191	3.6165741	-0.9447161
Au	0.0870209	3.5621742	-0.9105162
Au	1.5514205	1.0933759	-0.9936161
Au	-1.3163787	1.0690759	-0.9788161
Au	-4.2091769	1.0147759	-0.9445161
Au	-2.7447773	-1.4539224	-1.0277161
Au	3.0378191	-1.3762225	-1.0714161
Au	0.1450209	-1.4305224	-1.0371161
Au	1.6094205	-3.8993217	-1.1203161
Au	-1.2583787	-3.9237217	-1.1055161
Au	-4.1510769	-3.9780217	-1.0712161
Au	-2.8028773	3.5387742	-0.9011162
Au	-5.6338755	-1.4894224	-1.0532161
Au	4.4514187	1.1232758	-1.0142161

ORTHO

s-o-nh2

S	-0.6344495	0.1855304	-3.1968853
C	0.9953834	0.7280014	-3.6797785
C	2.0228729	-0.2303288	-3.8260371
C	1.2746593	2.0937252	-3.9693710
C	3.3249990	0.1490073	-4.1416020
H	1.7686186	-1.2769360	-3.6831945
C	2.6087272	2.4590529	-4.2479711
C	3.6176000	1.5097187	-4.3202448
H	4.1014683	-0.6032880	-4.2357521

H	2.8393458	3.5113938	-4.3937181
N	0.2660868	3.0307814	-3.9974537
H	0.5375764	3.9911386	-3.8141955
H	-0.5965315	2.7612834	-3.5268680
H	4.6354479	1.8269016	-4.5259497
Au	-4.2571789	-5.2925195	1.3061158
Au	-1.3637807	-5.2727195	1.3213158
Au	-2.7994793	-2.7857212	1.3109158
Au	0.0684189	-2.7951212	1.3061158
Au	2.9618171	-2.7753212	1.3213158
Au	1.5261185	-0.2884229	1.3109158
Au	-4.2571789	-0.2978229	1.3061158
Au	-1.3637807	-0.2780229	1.3213158
Au	-2.7994793	2.2089764	1.3109158
Au	0.0684189	2.1995764	1.3061158
Au	2.9618171	2.2193764	1.3213158
Au	1.5261185	-5.2831195	1.3109158
Au	4.4152167	-0.2859229	1.2659158
Au	-5.6997775	-2.7813212	1.3101158
Au	-4.2743789	-3.6929210	3.5948151
Au	-1.3808807	-3.7076210	3.5954151
Au	-2.7869793	-1.2045217	3.6588141
Au	0.0806189	-1.2475217	3.6388151
Au	2.9740171	-1.2622217	3.6395151
Au	1.5680185	1.2407766	3.7028141
Au	-4.2148789	1.2996766	3.7278141
Au	-1.3214807	1.2849766	3.7285141

Au	-2.7274793	3.7880749	3.7918141
Au	0.1401189	3.7450749	3.7719141
Au	3.0335171	3.7303749	3.7725141
Au	1.5085185	-3.7517210	3.5697152
Au	4.4567167	1.2104766	3.6429141
Au	-5.6869775	-1.1659217	3.6732141
Au	2.9181171	3.8764749	-0.9523826
Au	0.0254189	3.8220749	-0.9181826
Au	1.4898185	1.3532766	-1.0012826
Au	-1.3779807	1.3289766	-0.9864826
Au	-4.2707789	1.2746766	-0.9521826
Au	-2.8063793	-1.1940217	-1.0353826
Au	2.9762171	-1.1163217	-1.0790825
Au	0.0834189	-1.1706217	-1.0447825
Au	1.5478185	-3.6394210	-1.1279825
Au	-1.3199807	-3.6638210	-1.1131825
Au	-4.2126789	-3.7181210	-1.0788825
Au	-2.8644793	3.7986749	-0.9087826
Au	-5.6954775	-1.2295217	-1.0608825
Au	4.3898167	1.3831766	-1.0218826

s-o-och3

S	-0.4883381	-0.2071133	-2.9328618
C	0.8734132	0.8085461	-3.4934019
C	2.1788380	0.2997419	-3.6070470
C	0.6331130	2.1509830	-3.8806049
C	3.2419255	1.1176959	-3.9992225

H	2.3420688	-0.7490959	-3.3704355
C	1.7018079	2.9675204	-4.2587613
C	3.0010255	2.4561850	-4.3001287
H	4.2457497	0.7078610	-4.0476040
H	1.5297630	4.0114860	-4.4962884
C	-0.9441737	3.9455204	-4.0517030
O	-0.6610766	2.5630402	-3.8074215
H	-2.0126778	4.0535002	-3.8613772
H	-0.7111420	4.2158728	-5.0900146
H	-0.3835742	4.5850659	-3.3572018
H	3.8223560	3.1122917	-4.5713374
Au	-4.0886419	-5.6782950	1.5006725
Au	-1.1952437	-5.6584950	1.5158725
Au	-2.6309423	-3.1714967	1.5054725
Au	0.2369559	-3.1808967	1.5006725
Au	3.1303541	-3.1610967	1.5158725
Au	1.6946555	-0.6741984	1.5054725
Au	-4.0886419	-0.6835984	1.5006725
Au	-1.1952437	-0.6637984	1.5158725
Au	-2.6309423	1.8232009	1.5054725
Au	0.2369559	1.8138009	1.5006725
Au	3.1303541	1.8336009	1.5158725
Au	1.6946555	-5.6688950	1.5054725
Au	4.5837537	-0.6716984	1.4604725
Au	-5.5312405	-3.1670967	1.5046725
Au	-4.1058419	-4.0786964	3.7893719
Au	-1.2123437	-4.0933964	3.7899719

Au	-2.6184423	-1.5902971	3.8533709
Au	0.2491559	-1.6332971	3.8333719
Au	3.1425541	-1.6479971	3.8340719
Au	1.7365555	0.8550012	3.8973709
Au	-4.0463419	0.9139012	3.9223708
Au	-1.1529437	0.8992012	3.9230708
Au	-2.5589423	3.4022995	3.9863708
Au	0.3086559	3.3592995	3.9664708
Au	3.2020541	3.3445995	3.9670708
Au	1.6770555	-4.1374964	3.7642719
Au	4.6252537	0.8247012	3.8374709
Au	-5.5184405	-1.5516971	3.8677709
Au	3.0866541	3.4906994	-0.7578258
Au	0.1939559	3.4362995	-0.7236258
Au	1.6583555	0.9675012	-0.8067258
Au	-1.2094437	0.9432012	-0.7919258
Au	-4.1022419	0.8889012	-0.7576258
Au	-2.6378423	-1.5797971	-0.8408258
Au	3.1447541	-1.5020972	-0.8845258
Au	0.2519559	-1.5563971	-0.8502258
Au	1.7163555	-4.0251964	-0.9334258
Au	-1.1514437	-4.0495964	-0.9186258
Au	-4.0441419	-4.1038964	-0.8843258
Au	-2.6959423	3.4128995	-0.7142259
Au	-5.5269405	-1.6152971	-0.8663258
Au	4.5583537	0.9974011	-0.8273258

s-o-ch3

S	-0.5768201	-0.0432023	-3.0250144
C	0.7468322	1.0084650	-3.6084896
C	2.0718743	0.5250307	-3.6117340
C	0.4727414	2.3059460	-4.1009247
C	3.1322526	1.3465847	-3.9995708
H	2.2470422	-0.5100476	-3.3261999
C	1.5579221	3.1117001	-4.4617810
C	2.8754575	2.6576847	-4.3973751
H	4.1467846	0.9594047	-3.9748421
H	1.3579389	4.1282916	-4.7932047
H	3.6930248	3.3193059	-4.6667495
C	-0.9329053	2.8206972	-4.2050548
H	-1.5659008	2.1242618	-4.7659849
H	-1.3901668	2.9181957	-3.2081091
H	-0.9545599	3.8006456	-4.6898780
Au	-4.0663204	-5.5490714	1.4433695
Au	-1.1729222	-5.5292714	1.4585695
Au	-2.6086208	-3.0422731	1.4481695
Au	0.2592774	-3.0516731	1.4433695
Au	3.1526756	-3.0318731	1.4585695
Au	1.7169770	-0.5449748	1.4481695
Au	-4.0663204	-0.5543748	1.4433695
Au	-1.1729222	-0.5345748	1.4585695
Au	-2.6086208	1.9524245	1.4481695
Au	0.2592774	1.9430245	1.4433695
Au	3.1526756	1.9628245	1.4585695

Au	1.7169770	-5.5396714	1.4481695
Au	4.6060752	-0.5424748	1.4031695
Au	-5.5089190	-3.0378731	1.4473695
Au	-4.0835204	-3.9494729	3.7320688
Au	-1.1900222	-3.9641728	3.7326688
Au	-2.5961208	-1.4610736	3.7960678
Au	0.2714774	-1.5040735	3.7760688
Au	3.1648756	-1.5187735	3.7767688
Au	1.7588770	0.9842248	3.8400678
Au	-4.0240204	1.0431247	3.8650678
Au	-1.1306222	1.0284248	3.8657678
Au	-2.5366208	3.5315230	3.9290678
Au	0.3309774	3.4885231	3.9091678
Au	3.2243756	3.4738231	3.9097678
Au	1.6993770	-4.0082728	3.7069688
Au	4.6475752	0.9539248	3.7801678
Au	-5.4961190	-1.4224736	3.8104678
Au	3.1089756	3.6199230	-0.8151289
Au	0.2162774	3.5655230	-0.7809289
Au	1.6806770	1.0967247	-0.8640289
Au	-1.1871222	1.0724247	-0.8492289
Au	-4.0799204	1.0181248	-0.8149289
Au	-2.6155208	-1.4505736	-0.8981289
Au	3.1670756	-1.3728736	-0.9418289
Au	0.2742774	-1.4271736	-0.9075289
Au	1.7386770	-3.8959729	-0.9907288
Au	-1.1291222	-3.9203729	-0.9759288

Au	-4.0218204	-3.9746728	-0.9416289
Au	-2.6736208	3.5421230	-0.7715289
Au	-5.5046190	-1.4860735	-0.9236289
Au	4.5806752	1.1266247	-0.8846289

s-o-f

S	-0.6555222	0.1437381	-3.2711205
C	0.7091032	1.1372487	-3.8368410
C	2.0194709	0.6221380	-3.9296194
C	0.5271807	2.4731352	-4.2260290
C	3.0883241	1.4292589	-4.3211059
H	2.1755702	-0.4253698	-3.6825466
C	1.5793805	3.2913014	-4.6050571
C	2.8738759	2.7704918	-4.6404408
H	4.0883402	1.0079733	-4.3513019
H	1.3748010	4.3311545	-4.8387330
F	-0.7170017	3.0062700	-4.1860547
H	3.7068994	3.4122156	-4.9079829
Au	-4.1641081	-5.3723364	1.2017533
Au	-1.2707099	-5.3525364	1.2169533
Au	-2.7064085	-2.8655381	1.2065533
Au	0.1614896	-2.8749381	1.2017533
Au	3.0548878	-2.8551381	1.2169533
Au	1.6191892	-0.3682398	1.2065533
Au	-4.1641081	-0.3776398	1.2017533
Au	-1.2707099	-0.3578398	1.2169533
Au	-2.7064085	2.1291595	1.2065533

Au	0.1614896	2.1197595	1.2017533
Au	3.0548878	2.1395595	1.2169533
Au	1.6191892	-5.3629364	1.2065533
Au	4.5082874	-0.3657398	1.1615534
Au	-5.6067067	-2.8611381	1.2057533
Au	-4.1813081	-3.7727378	3.4904527
Au	-1.2878099	-3.7874378	3.4910527
Au	-2.6939085	-1.2843385	3.5544517
Au	0.1736896	-1.3273385	3.5344527
Au	3.0670878	-1.3420385	3.5351527
Au	1.6610892	1.1609598	3.5984517
Au	-4.1218081	1.2198598	3.6234517
Au	-1.2284100	1.2051598	3.6241517
Au	-2.6344086	3.7082581	3.6874516
Au	0.2331896	3.6652581	3.6675516
Au	3.1265878	3.6505581	3.6681516
Au	1.6015892	-3.8315378	3.4653527
Au	4.5497874	1.1306598	3.5385517
Au	-5.5939067	-1.2457385	3.5688517
Au	3.0111878	3.7966580	-1.0567450
Au	0.1184897	3.7422581	-1.0225450
Au	1.5828892	1.2734598	-1.1056450
Au	-1.2849099	1.2491598	-1.0908450
Au	-4.1777081	1.1948598	-1.0565450
Au	-2.7133085	-1.2738385	-1.1397450
Au	3.0692878	-1.1961385	-1.1834450
Au	0.1764896	-1.2504385	-1.1491450

Au	1.6408892	-3.7192378	-1.2323450
Au	-1.2269100	-3.7436378	-1.2175450
Au	-4.1196081	-3.7979378	-1.1832450
Au	-2.7714085	3.7188581	-1.0131450
Au	-5.6024067	-1.3093385	-1.1652450
Au	4.4828874	1.3033597	-1.1262450

s-o-cl

S	-0.4895622	0.1314927	-3.2528957
C	0.8442090	1.1566035	-3.8523776
C	2.1228695	0.6585937	-4.1790662
C	0.6257513	2.5366578	-4.0416661
C	3.1624729	1.5076196	-4.5598365
Cl	2.4725931	-1.0494325	-4.0531168
C	1.6496594	3.3830793	-4.4415709
C	2.9306463	2.8744375	-4.6765209
H	4.1455285	1.0876725	-4.7427555
H	1.4557804	4.4471640	-4.5359583
H	-0.3613308	2.9291192	-3.8138402
H	3.7470927	3.5358844	-4.9482707
Au	-4.1510996	-5.3654463	1.1999246
Au	-1.2577014	-5.3456463	1.2151246
Au	-2.6934000	-2.8586480	1.2047246
Au	0.1744982	-2.8680480	1.1999246
Au	3.0678963	-2.8482480	1.2151246
Au	1.6321977	-0.3613497	1.2047246
Au	-4.1510996	-0.3707497	1.1999246

Au	-1.2577014	-0.3509497	1.2151246
Au	-2.6934000	2.1360496	1.2047246
Au	0.1744982	2.1266496	1.1999246
Au	3.0678963	2.1464496	1.2151246
Au	1.6321977	-5.3560463	1.2047246
Au	4.5212959	-0.3588497	1.1597246
Au	-5.5936982	-2.8542480	1.2039246
Au	-4.1682996	-3.7658478	3.4886239
Au	-1.2748014	-3.7805478	3.4892239
Au	-2.6809000	-1.2774485	3.5526229
Au	0.1866981	-1.3204485	3.5326239
Au	3.0800963	-1.3351485	3.5333239
Au	1.6740977	1.1678498	3.5966229
Au	-4.1087996	1.2267498	3.6216229
Au	-1.2154015	1.2120498	3.6223229
Au	-2.6214001	3.7151481	3.6856229
Au	0.2461981	3.6721481	3.6657229
Au	3.1395963	3.6574481	3.6663229
Au	1.6145977	-3.8246478	3.4635239
Au	4.5627959	1.1375498	3.5367229
Au	-5.5808982	-1.2388485	3.5670229
Au	3.0241964	3.8035481	-1.0585738
Au	0.1314982	3.7491481	-1.0243738
Au	1.5958978	1.2803498	-1.1074738
Au	-1.2719014	1.2560498	-1.0926738
Au	-4.1646996	1.2017498	-1.0583738
Au	-2.7003000	-1.2669485	-1.1415738

Au	3.0822963	-1.1892485	-1.1852738
Au	0.1894981	-1.2435485	-1.1509738
Au	1.6538977	-3.7123478	-1.2341737
Au	-1.2139015	-3.7367478	-1.2193738
Au	-4.1065996	-3.7910478	-1.1850738
Au	-2.7584000	3.7257481	-1.0149738
Au	-5.5893982	-1.3024485	-1.1670738
Au	4.4958959	1.3102498	-1.1280738

s-o-ococh3

S	-0.6794627	0.3142742	-2.7121169
C	0.7017823	1.2646148	-3.3239823
C	1.9610652	0.6887215	-3.5866531
C	0.5539663	2.6417222	-3.5780234
C	3.0443739	1.4651767	-3.9921428
C	1.6264958	3.4158336	-3.9981642
H	-0.4124823	3.0954534	-3.3739578
C	2.8822230	2.8331342	-4.1917597
H	4.0092505	0.9833956	-4.1127403
H	1.4906331	4.4836622	-4.1390401
H	3.7332092	3.4410180	-4.4821021
C	0.9466088	-1.3993954	-5.2312075
H	1.4446301	-0.6149638	-5.8080830
C	1.7050959	-1.7204990	-3.9734919
H	-0.0506861	-1.0369801	-4.9561979
H	0.8491809	-2.3132790	-5.8168642
O	1.9105726	-2.8288935	-3.5418298

O	2.2379889	-0.6479110	-3.2726015
Au	-4.2855768	-5.1479868	1.7473804
Au	-1.3921776	-5.1281868	1.7625804
Au	-2.8278772	-2.6411885	1.7521804
Au	0.0400210	-2.6505885	1.7473804
Au	2.9334191	-2.6307885	1.7625804
Au	1.4977205	-0.1438902	1.7521804
Au	-4.2855768	-0.1532902	1.7473804
Au	-1.3921776	-0.1334902	1.7625804
Au	-2.8278772	2.3535091	1.7521804
Au	0.0400210	2.3441091	1.7473804
Au	2.9334191	2.3639091	1.7625804
Au	1.4977205	-5.1385868	1.7521804
Au	4.3868187	-0.1413902	1.7071804
Au	-5.7281754	-2.6367885	1.7513804
Au	-4.3027768	-3.5483882	4.0360787
Au	-1.4092776	-3.5630882	4.0366787
Au	-2.8153772	-1.0599889	4.1000787
Au	0.0522210	-1.1029889	4.0800787
Au	2.9456191	-1.1176889	4.0807787
Au	1.5396205	1.3853094	4.1440787
Au	-4.2432768	1.4442094	4.1690787
Au	-1.3498777	1.4295094	4.1697787
Au	-2.7558773	3.9326077	4.2330787
Au	0.1117209	3.8896077	4.2131787
Au	3.0051191	3.8749077	4.2137787
Au	1.4801206	-3.6071882	4.0109788

Au	4.4283187	1.3550094	4.0841787
Au	-5.7153754	-1.0213889	4.1144787
Au	2.8897192	4.0210077	-0.5111190
Au	-0.0029790	3.9666077	-0.4769190
Au	1.4614206	1.4978094	-0.5600190
Au	-1.4063776	1.4735094	-0.5452190
Au	-4.2991768	1.4192094	-0.5109190
Au	-2.8347772	-1.0494889	-0.5941190
Au	2.9478191	-0.9717889	-0.6378189
Au	0.0550210	-1.0260889	-0.6035190
Au	1.5194205	-3.4948882	-0.6867189
Au	-1.3483777	-3.5192882	-0.6719189
Au	-4.2410768	-3.5735882	-0.6376189
Au	-2.8928772	3.9432077	-0.4675190
Au	-5.7238754	-1.0849889	-0.6196189
Au	4.3614187	1.5277094	-0.5806190

s-o-cf3

S	-0.7783166	0.2682574	-3.1070401
C	0.3101077	1.5647033	-3.6732069
C	1.6502393	1.4026585	-4.0956670
C	-0.2076086	2.8793507	-3.6548436
C	2.4388326	2.5315881	-4.3717402
C	0.5745960	3.9846441	-3.9542600
C	1.9244649	3.8166966	-4.2803583
H	3.4765389	2.3824286	-4.6507750
H	0.1363607	4.9776405	-3.9057717

H	2.5600023	4.6738609	-4.4772745
H	-1.2482067	3.0062350	-3.3691566
C	2.3129658	0.0779777	-4.3933498
F	2.3121609	-0.1524471	-5.7485698
F	3.6295832	0.0803634	-4.0228492
F	1.7531923	-1.0058181	-3.8261944
Au	-4.2269718	-5.5134831	1.4708684
Au	-1.3335736	-5.4936831	1.4860684
Au	-2.7692722	-3.0066848	1.4756684
Au	0.0986260	-3.0160848	1.4708684
Au	2.9920242	-2.9962848	1.4860684
Au	1.5563256	-0.5093865	1.4756684
Au	-4.2269718	-0.5187865	1.4708684
Au	-1.3335736	-0.4989865	1.4860684
Au	-2.7692722	1.9880128	1.4756684
Au	0.0986260	1.9786128	1.4708684
Au	2.9920242	1.9984128	1.4860684
Au	1.5563256	-5.5040831	1.4756684
Au	4.4454238	-0.5068865	1.4306684
Au	-5.6695704	-3.0022848	1.4748684
Au	-4.2441718	-3.9138846	3.7595678
Au	-1.3506736	-3.9285846	3.7601678
Au	-2.7567722	-1.4254853	3.8235667
Au	0.1108260	-1.4684853	3.8035677
Au	3.0042242	-1.4831853	3.8042677
Au	1.5982256	1.0198130	3.8675667
Au	-4.1846718	1.0787130	3.8925667

Au	-1.2912736	1.0640130	3.8932667
Au	-2.6972722	3.5671113	3.9565667
Au	0.1703260	3.5241113	3.9366667
Au	3.0637242	3.5094113	3.9372667
Au	1.5387256	-3.9726846	3.7344678
Au	4.4869238	0.9895130	3.8076667
Au	-5.6567704	-1.3868853	3.8379667
Au	2.9483242	3.6555113	-0.7876300
Au	0.0556260	3.6011113	-0.7534300
Au	1.5200256	1.1323130	-0.8365299
Au	-1.3477736	1.1080130	-0.8217300
Au	-4.2405718	1.0537130	-0.7874300
Au	-2.7761722	-1.4149853	-0.8706299
Au	3.0064242	-1.3372853	-0.9143299
Au	0.1136260	-1.3915853	-0.8800299
Au	1.5780256	-3.8603846	-0.9632299
Au	-1.2897736	-3.8847846	-0.9484299
Au	-4.1824718	-3.9390846	-0.9141299
Au	-2.8342722	3.5777113	-0.7440300
Au	-5.6652704	-1.4504853	-0.8961299
Au	4.4200238	1.1622130	-0.8571299

s-o-cn

S	-0.7056726	0.1251590	-3.2842172
C	0.5645816	1.2276186	-3.8148024
C	1.9097035	0.8023941	-3.8764376
C	0.2817595	2.5787219	-4.1522622

C	2.9304354	1.6911997	-4.2135132
H	2.1308945	-0.2375390	-3.6545820
C	1.3200320	3.4604612	-4.4974400
C	2.6401224	3.0254590	-4.5146938
H	3.9579348	1.3400643	-4.2142493
H	1.0749880	4.4941926	-4.7199853
H	3.4384184	3.7209606	-4.7516114
C	-1.0442705	3.0901333	-4.0502657
N	-2.1022252	3.5638943	-3.9280963
Au	-4.0563036	-5.4901577	1.2759903
Au	-1.1629054	-5.4703577	1.2911903
Au	-2.5986040	-2.9833594	1.2807903
Au	0.2692942	-2.9927594	1.2759903
Au	3.1626924	-2.9729594	1.2911903
Au	1.7269938	-0.4860611	1.2807903
Au	-4.0563036	-0.4954611	1.2759903
Au	-1.1629054	-0.4756611	1.2911903
Au	-2.5986040	2.0113382	1.2807903
Au	0.2692942	2.0019382	1.2759903
Au	3.1626924	2.0217382	1.2911903
Au	1.7269938	-5.4807577	1.2807903
Au	4.6160920	-0.4835611	1.2357903
Au	-5.4989022	-2.9789594	1.2799903
Au	-4.0735036	-3.8905592	3.5646896
Au	-1.1800054	-3.9052591	3.5652896
Au	-2.5861040	-1.4021598	3.6286886
Au	0.2814942	-1.4451598	3.6086896

Au	3.1748924	-1.4598598	3.6093896
Au	1.7688938	1.0431385	3.6726886
Au	-4.0140036	1.1020384	3.6976886
Au	-1.1206054	1.0873385	3.6983886
Au	-2.5266040	3.5904367	3.7616886
Au	0.3409942	3.5474368	3.7417886
Au	3.2343924	3.5327368	3.7423886
Au	1.7093938	-3.9493591	3.5395897
Au	4.6575920	1.0128385	3.6127886
Au	-5.4861022	-1.3635599	3.6430886
Au	3.1189924	3.6788367	-0.9825081
Au	0.2262942	3.6244367	-0.9483081
Au	1.6906938	1.1556384	-1.0314081
Au	-1.1771054	1.1313384	-1.0166081
Au	-4.0699036	1.0770385	-0.9823081
Au	-2.6055040	-1.3916599	-1.0655081
Au	3.1770924	-1.3139599	-1.1092080
Au	0.2842942	-1.3682599	-1.0749080
Au	1.7486938	-3.8370592	-1.1581080
Au	-1.1191054	-3.8614592	-1.1433080
Au	-4.0118036	-3.9157591	-1.1090080
Au	-2.6636040	3.6010367	-0.9389081
Au	-5.4946022	-1.4271598	-1.0910080
Au	4.5906920	1.1855384	-1.0520081

s-o-no2

S	-0.7332460	0.1769765	-3.1323175

C	0.6263985	1.1095149	-3.7808609
C	1.9081106	0.5699327	-4.0544239
C	0.4867687	2.5038929	-3.9448629
C	3.0082282	1.3837095	-4.3512488
C	1.5608158	3.3054781	-4.2975223
C	2.8363530	2.7528757	-4.4774648
H	3.9773743	0.9140262	-4.4733939
H	1.4140365	4.3776308	-4.3876218
H	3.6860470	3.3875791	-4.7048806
H	-0.4820298	2.9440741	-3.7264610
N	2.1475328	-0.8532972	-3.9353263
O	1.2279632	-1.6408208	-4.1707611
O	3.2811631	-1.2154659	-3.5031781
Au	-4.2139322	-5.2825465	1.3390339
Au	-1.3205340	-5.2627465	1.3542339
Au	-2.7562326	-2.7757482	1.3438339
Au	0.1116656	-2.7851482	1.3390339
Au	3.0050638	-2.7653482	1.3542339
Au	1.5693652	-0.2784499	1.3438339
Au	-4.2139322	-0.2878499	1.3390339
Au	-1.3205340	-0.2680499	1.3542339
Au	-2.7562326	2.2189494	1.3438339
Au	0.1116656	2.2095494	1.3390339
Au	3.0050638	2.2293494	1.3542339
Au	1.5693652	-5.2731465	1.3438339
Au	4.4584634	-0.2759499	1.2988339
Au	-5.6565308	-2.7713482	1.3430339

Au	-4.2311322	-3.6829479	3.6277333
Au	-1.3376340	-3.6976479	3.6283333
Au	-2.7437326	-1.1945486	3.6917323
Au	0.1238656	-1.2375486	3.6717333
Au	3.0172638	-1.2522486	3.6724333
Au	1.6112652	1.2507497	3.7357323
Au	-4.1716322	1.3096497	3.7607323
Au	-1.2782340	1.2949497	3.7614323
Au	-2.6842326	3.7980480	3.8247322
Au	0.1833656	3.7550480	3.8048322
Au	3.0767637	3.7403480	3.8054322
Au	1.5517652	-3.7417479	3.6026333
Au	4.4999633	1.2204497	3.6758323
Au	-5.6437308	-1.1559486	3.7061323
Au	2.9613638	3.8864480	-0.9194644
Au	0.0686656	3.8320480	-0.8852644
Au	1.5330652	1.3632497	-0.9683644
Au	-1.3347340	1.3389497	-0.9535644
Au	-4.2275322	1.2846497	-0.9192644
Au	-2.7631326	-1.1840486	-1.0024644
Au	3.0194638	-1.1063486	-1.0461644
Au	0.1266656	-1.1606486	-1.0118644
Au	1.5910652	-3.6294479	-1.0950644
Au	-1.2767340	-3.6538479	-1.0802644
Au	-4.1694322	-3.7081479	-1.0459644
Au	-2.8212326	3.8086480	-0.8758644
Au	-5.6522308	-1.2195486	-1.0279644

Au 4.4330634 1.3931497 -0.9889644

Coordinates from the Selenium-based Ligands

META

se-m-nh2

Se -0.8224831 0.0695508 -3.2094624

C 0.9975417 0.6098377 -3.6967188

C 1.9571155 -0.3948182 -3.9161193

C 1.3347047 1.9582801 -3.8161035

C 3.2674428 -0.0134284 -4.2134378

H 1.6791235 -1.4408887 -3.8420249

C 2.6601095 2.3345280 -4.1144019

C 3.6298545 1.3310897 -4.2895701

H 4.0250834 -0.7788075 -4.3571350

H 4.6597772 1.6090460 -4.4965322

H 0.5817519 2.7245463 -3.6506847

N	2.9807334	3.6813547	-4.2384469
H	2.3526244	4.3061161	-3.7364463
H	3.9491566	3.9045084	-4.0320412
Au	-4.4117667	-5.2868588	1.3070186
Au	-1.5183685	-5.2670588	1.3222186
Au	-2.9540671	-2.7800605	1.3118186
Au	-0.0861689	-2.7894605	1.3070186
Au	2.8072293	-2.7696605	1.3222186
Au	1.3715307	-0.2827622	1.3118186
Au	-4.4117667	-0.2921622	1.3070186
Au	-1.5183685	-0.2723622	1.3222186
Au	-2.9540671	2.2146371	1.3118186
Au	-0.0861689	2.2052371	1.3070186
Au	2.8072293	2.2250371	1.3222186
Au	1.3715307	-5.2774588	1.3118186
Au	4.2606289	-0.2802622	1.2668186
Au	-5.8543653	-2.7756605	1.3110186
Au	-4.4289667	-3.6872602	3.5957180
Au	-1.5354685	-3.7019602	3.5963180
Au	-2.9415671	-1.1988609	3.6597169
Au	-0.0739689	-1.2418609	3.6397179
Au	2.8194293	-1.2565609	3.6404179
Au	1.4134307	1.2464374	3.7037169
Au	-4.3694667	1.3053374	3.7287169
Au	-1.4760685	1.2906374	3.7294169
Au	-2.8820671	3.7937357	3.7927169
Au	-0.0144689	3.7507357	3.7728169

Au	2.8789292	3.7360357	3.7734169
Au	1.3539307	-3.7460602	3.5706180
Au	4.3021288	1.2161374	3.6438169
Au	-5.8415653	-1.1602609	3.6741169
Au	2.7635293	3.8821357	-0.9514798
Au	-0.1291689	3.8277357	-0.9172798
Au	1.3352307	1.3589374	-1.0003798
Au	-1.5325685	1.3346374	-0.9855798
Au	-4.4253667	1.2803374	-0.9512798
Au	-2.9609671	-1.1883609	-1.0344797
Au	2.8216293	-1.1106609	-1.0781797
Au	-0.0711689	-1.1649609	-1.0438797
Au	1.3932307	-3.6337602	-1.1270797
Au	-1.4745685	-3.6581602	-1.1122797
Au	-4.3672667	-3.7124602	-1.0779797
Au	-3.0190671	3.8043357	-0.9078798
Au	-5.8500653	-1.2238609	-1.0599797
Au	4.2352289	1.3888374	-1.0209797

se-m-och3

Se	-0.5705403	-0.3230028	-3.0149557
C	0.9275749	0.8281511	-3.5469244
C	2.2092401	0.2964212	-3.7263251
C	0.6701372	2.1831577	-3.7992377
C	3.2497007	1.1484633	-4.1158715
H	2.3916399	-0.7591744	-3.5467656
C	1.7267287	3.0215569	-4.1653932

C	3.0218061	2.5018604	-4.3244325
H	4.2528672	0.7471187	-4.2272582
H	3.8280585	3.1783165	-4.5869048
H	-0.3302805	2.5684071	-3.6393241
C	0.3338155	4.9557661	-4.0203688
O	1.5958153	4.3672986	-4.3616279
H	0.4641431	6.0293230	-4.1594469
H	0.0798916	4.7403145	-2.9689792
H	-0.4667605	4.5855730	-4.6736274
Au	-4.1767806	-5.7670950	1.4801705
Au	-1.2833824	-5.7472950	1.4953705
Au	-2.7190810	-3.2602967	1.4849705
Au	0.1488172	-3.2696967	1.4801705
Au	3.0422153	-3.2498967	1.4953705
Au	1.6065167	-0.7629984	1.4849705
Au	-4.1767806	-0.7723984	1.4801705
Au	-1.2833824	-0.7525984	1.4953705
Au	-2.7190810	1.7344009	1.4849705
Au	0.1488172	1.7250009	1.4801705
Au	3.0422153	1.7448009	1.4953705
Au	1.6065167	-5.7576950	1.4849705
Au	4.4956149	-0.7604984	1.4399705
Au	-5.6193792	-3.2558967	1.4841705
Au	-4.1939806	-4.1674964	3.7688699
Au	-1.3004824	-4.1821964	3.7694699
Au	-2.7065810	-1.6790971	3.8328689
Au	0.1610171	-1.7220971	3.8128699

Au	3.0544153	-1.7367971	3.8135699
Au	1.6484167	0.7662012	3.8768689
Au	-4.1344806	0.8251012	3.9018689
Au	-1.2410825	0.8104012	3.9025689
Au	-2.6470811	3.3134995	3.9658688
Au	0.2205171	3.2704995	3.9459688
Au	3.1139153	3.2557995	3.9465688
Au	1.5889167	-4.2262964	3.7437699
Au	4.5371149	0.7359012	3.8169689
Au	-5.6065792	-1.6404971	3.8472689
Au	2.9985154	3.4018995	-0.7783278
Au	0.1058172	3.3474995	-0.7441278
Au	1.5702168	0.8787012	-0.8272278
Au	-1.2975824	0.8544012	-0.8124278
Au	-4.1903806	0.8001012	-0.7781278
Au	-2.7259810	-1.6685971	-0.8613278
Au	3.0566153	-1.5908971	-0.9050278
Au	0.1638171	-1.6451971	-0.8707278
Au	1.6282167	-4.1139964	-0.9539278
Au	-1.2395825	-4.1383964	-0.9391278
Au	-4.1322806	-4.1926964	-0.9048278
Au	-2.7840810	3.3240995	-0.7347278
Au	-5.6150792	-1.7040971	-0.8868278
Au	4.4702149	0.9086012	-0.8478278

se-m-ch3

Se	-0.6528585	-0.2341474	-3.0692713

C	0.8585538	0.8931534	-3.6088758
C	2.1354204	0.3462682	-3.8000952
C	0.6353607	2.2535903	-3.8485928
C	3.1872191	1.1862733	-4.1684831
H	2.3019503	-0.7155925	-3.6374207
C	1.6881914	3.1005957	-4.2068709
C	2.9692307	2.5520189	-4.3552267
H	4.1843561	0.7719831	-4.2872270
H	3.8034347	3.2010356	-4.6072204
H	-0.3592521	2.6646572	-3.6903920
C	1.4500023	4.5840872	-4.3185082
H	0.4966322	4.8010121	-4.8111533
H	1.4076066	5.0227159	-3.3108188
H	2.2535419	5.0817667	-4.8696536
Au	-4.2476167	-5.6585220	1.4256747
Au	-1.3542185	-5.6387220	1.4408747
Au	-2.7899171	-3.1517237	1.4304747
Au	0.0779811	-3.1611237	1.4256747
Au	2.9713793	-3.1413237	1.4408747
Au	1.5356807	-0.6544254	1.4304747
Au	-4.2476167	-0.6638254	1.4256747
Au	-1.3542185	-0.6440254	1.4408747
Au	-2.7899171	1.8429739	1.4304747
Au	0.0779811	1.8335739	1.4256747
Au	2.9713793	1.8533739	1.4408747
Au	1.5356807	-5.6491220	1.4304747
Au	4.4247789	-0.6519254	1.3854747

Au	-5.6902153	-3.1473237	1.4296747
Au	-4.2648167	-4.0589235	3.7143740
Au	-1.3713185	-4.0736235	3.7149740
Au	-2.7774171	-1.5705242	3.7783730
Au	0.0901811	-1.6135241	3.7583740
Au	2.9835793	-1.6282241	3.7590740
Au	1.5775807	0.8747742	3.8223730
Au	-4.2053167	0.9336741	3.8473730
Au	-1.3119185	0.9189741	3.8480730
Au	-2.7179171	3.4220724	3.9113730
Au	0.1496811	3.3790725	3.8914730
Au	3.0430793	3.3643725	3.8920730
Au	1.5180807	-4.1177234	3.6892740
Au	4.4662789	0.8444742	3.7624730
Au	-5.6774153	-1.5319242	3.7927730
Au	2.9276793	3.5104724	-0.8328237
Au	0.0349811	3.4560724	-0.7986237
Au	1.4993807	0.9872741	-0.8817237
Au	-1.3684185	0.9629741	-0.8669237
Au	-4.2612167	0.9086741	-0.8326237
Au	-2.7968171	-1.5600242	-0.9158237
Au	2.9857793	-1.4823242	-0.9595237
Au	0.0929811	-1.5366242	-0.9252237
Au	1.5573807	-4.0054235	-1.0084236
Au	-1.3104185	-4.0298235	-0.9936236
Au	-4.2031167	-4.0841235	-0.9593237
Au	-2.8549171	3.4326724	-0.7892237

Au	-5.6859153	-1.5955241	-0.9413237
Au	4.3993789	1.0171741	-0.9023237

se-m-h

Se	-0.5802829	0.0651270	-3.3025677
C	0.9089124	1.2118007	-3.8612433
C	2.1989358	0.6825585	-4.0102556
C	0.6692481	2.5604295	-4.1590926
C	3.2531353	1.5146717	-4.3927432
H	2.3755423	-0.3697891	-3.8019337
C	1.7258580	3.3794114	-4.5497339
C	3.0212893	2.8658469	-4.6514122
H	4.2562693	1.1043607	-4.4648049
H	3.8465372	3.5171689	-4.9219649
H	-0.3287478	2.9700146	-4.0318579
H	1.5398233	4.4321804	-4.7433851
Au	-4.1649015	-5.3828982	1.1947696
Au	-1.2715033	-5.3630982	1.2099696
Au	-2.7072019	-2.8760999	1.1995696
Au	0.1606963	-2.8854999	1.1947696
Au	3.0540945	-2.8656999	1.2099696
Au	1.6183959	-0.3788016	1.1995696
Au	-4.1649015	-0.3882016	1.1947696
Au	-1.2715033	-0.3684016	1.2099696
Au	-2.7072019	2.1185977	1.1995696
Au	0.1606963	2.1091977	1.1947696
Au	3.0540945	2.1289977	1.2099696

Au	1.6183959	-5.3734982	1.1995696
Au	4.5074941	-0.3763016	1.1545696
Au	-5.6075001	-2.8716999	1.1987696
Au	-4.1821015	-3.7832996	3.4834689
Au	-1.2886033	-3.7979996	3.4840689
Au	-2.6947019	-1.2949003	3.5474679
Au	0.1728963	-1.3379003	3.5274689
Au	3.0662945	-1.3526003	3.5281689
Au	1.6602959	1.1503980	3.5914679
Au	-4.1226015	1.2092980	3.6164679
Au	-1.2292033	1.1945980	3.6171679
Au	-2.6352019	3.6976963	3.6804679
Au	0.2323963	3.6546963	3.6605679
Au	3.1257945	3.6399963	3.6611679
Au	1.6007959	-3.8420996	3.4583689
Au	4.5489941	1.1200980	3.5315679
Au	-5.5947001	-1.2563003	3.5618679
Au	3.0103945	3.7860962	-1.0637288
Au	0.1176963	3.7316963	-1.0295288
Au	1.5820959	1.2628980	-1.1126288
Au	-1.2857033	1.2385980	-1.0978288
Au	-4.1785015	1.1842980	-1.0635288
Au	-2.7141019	-1.2844003	-1.1467288
Au	3.0684945	-1.2067003	-1.1904288
Au	0.1756963	-1.2610003	-1.1561288
Au	1.6400959	-3.7297996	-1.2393287
Au	-1.2277033	-3.7541996	-1.2245288

Au	-4.1204015	-3.8084996	-1.1902288
Au	-2.7722019	3.7082963	-1.0201288
Au	-5.6032001	-1.3199003	-1.1722288
Au	4.4820941	1.2927980	-1.1332288

se-m-f

Se	-0.5935028	0.0542075	-3.2986883
C	0.9157487	1.1686810	-3.8627066
C	2.1984358	0.6203406	-4.0093097
C	0.6868023	2.5160174	-4.1694048
C	3.2627048	1.4359478	-4.4001002
H	2.3610079	-0.4321621	-3.7944357
C	1.7668769	3.2891114	-4.5643723
C	3.0597419	2.7885861	-4.6718488
H	4.2604683	1.0140271	-4.4739899
H	3.8735049	3.4474545	-4.9517127
H	-0.2930929	2.9659357	-4.0480123
F	1.5548157	4.6055712	-4.8307194
Au	-4.1689285	-5.3719330	1.1991253
Au	-1.2755304	-5.3521330	1.2143253
Au	-2.7112290	-2.8651347	1.2039253
Au	0.1566692	-2.8745347	1.1991253
Au	3.0500674	-2.8547347	1.2143253
Au	1.6143688	-0.3678364	1.2039253
Au	-4.1689285	-0.3772364	1.1991253
Au	-1.2755304	-0.3574364	1.2143253
Au	-2.7112290	2.1295629	1.2039253

Au	0.1566692	2.1201629	1.1991253
Au	3.0500674	2.1399629	1.2143253
Au	1.6143688	-5.3625330	1.2039253
Au	4.5034670	-0.3653364	1.1589253
Au	-5.6115271	-2.8607347	1.2031253
Au	-4.1861285	-3.7723344	3.4878246
Au	-1.2926304	-3.7870344	3.4884246
Au	-2.6987290	-1.2839351	3.5518236
Au	0.1688692	-1.3269351	3.5318246
Au	3.0622674	-1.3416351	3.5325246
Au	1.6562688	1.1613632	3.5958236
Au	-4.1266286	1.2202632	3.6208236
Au	-1.2332304	1.2055632	3.6215236
Au	-2.6392290	3.7086615	3.6848236
Au	0.2283692	3.6656615	3.6649236
Au	3.1217674	3.6509615	3.6655236
Au	1.5967688	-3.8311344	3.4627246
Au	4.5449670	1.1310632	3.5359236
Au	-5.5987271	-1.2453352	3.5662236
Au	3.0063674	3.7970614	-1.0593731
Au	0.1136692	3.7426614	-1.0251731
Au	1.5780688	1.2738631	-1.1082731
Au	-1.2897304	1.2495631	-1.0934731
Au	-4.1825285	1.1952632	-1.0591731
Au	-2.7181290	-1.2734351	-1.1423731
Au	3.0644674	-1.1957352	-1.1860730
Au	0.1716692	-1.2500352	-1.1517731

Au	1.6360688	-3.7188345	-1.2349730
Au	-1.2317304	-3.7432345	-1.2201730
Au	-4.1244286	-3.7975344	-1.1858730
Au	-2.7762289	3.7192615	-1.0157731
Au	-5.6072271	-1.3089351	-1.1678731
Au	4.4780670	1.3037631	-1.1288731

se-m-cl

Se	-0.5945373	0.0501013	-3.3221058
C	0.9183139	1.1654958	-3.8704764
C	2.1882415	0.6072223	-4.0697775
C	0.7088976	2.5343518	-4.0843802
C	3.2587125	1.4309292	-4.4217498
H	2.3389600	-0.4574393	-3.9145536
C	1.7954769	3.3328797	-4.4253895
C	3.0769297	2.8043881	-4.5856304
H	4.2495037	1.0022046	-4.5380240
H	3.9102718	3.4573619	-4.8165722
H	-0.2690267	2.9751010	-3.9212430
Cl	1.5540763	5.0594364	-4.5604751
Au	-4.1708603	-5.3835566	1.1860146
Au	-1.2774621	-5.3637566	1.2012146
Au	-2.7131607	-2.8767583	1.1908146
Au	0.1547375	-2.8861583	1.1860146
Au	3.0481357	-2.8663583	1.2012146
Au	1.6124371	-0.3794600	1.1908146
Au	-4.1708603	-0.3888600	1.1860146

Au	-1.2774621	-0.3690600	1.2012146
Au	-2.7131607	2.1179393	1.1908146
Au	0.1547375	2.1085393	1.1860146
Au	3.0481357	2.1283393	1.2012146
Au	1.6124371	-5.3741566	1.1908146
Au	4.5015353	-0.3769600	1.1458146
Au	-5.6134589	-2.8723583	1.1900146
Au	-4.1880603	-3.7839580	3.4747139
Au	-1.2945621	-3.7986580	3.4753139
Au	-2.7006607	-1.2955587	3.5387129
Au	0.1669375	-1.3385587	3.5187139
Au	3.0603357	-1.3532587	3.5194139
Au	1.6543371	1.1497396	3.5827129
Au	-4.1285603	1.2086396	3.6077129
Au	-1.2351621	1.1939396	3.6084129
Au	-2.6411607	3.6970379	3.6717129
Au	0.2264375	3.6540379	3.6518129
Au	3.1198357	3.6393379	3.6524129
Au	1.5948371	-3.8427580	3.4496139
Au	4.5430353	1.1194396	3.5228129
Au	-5.6006589	-1.2569587	3.5531129
Au	3.0044357	3.7854379	-1.0724838
Au	0.1117375	3.7310379	-1.0382838
Au	1.5761371	1.2622396	-1.1213838
Au	-1.2916621	1.2379396	-1.1065838
Au	-4.1844603	1.1836396	-1.0722838
Au	-2.7200607	-1.2850587	-1.1554838

Au	3.0625357	-1.2073587	-1.1991838
Au	0.1697375	-1.2616587	-1.1648838
Au	1.6341371	-3.7304580	-1.2480837
Au	-1.2336621	-3.7548580	-1.2332837
Au	-4.1263603	-3.8091580	-1.1989838
Au	-2.7781607	3.7076379	-1.0288838
Au	-5.6091589	-1.3205587	-1.1809838
Au	4.4761353	1.2921396	-1.1419838

se-m-ococh3

Se	-0.6206515	-0.3995615	-2.6764927
C	0.8206466	0.7981505	-3.2314806
C	2.1290229	0.3127314	-3.3760656
C	0.5369687	2.1432693	-3.4964244
C	3.1550052	1.1852349	-3.7464956
H	2.3354441	-0.7361266	-3.1830060
C	1.5724729	2.9849361	-3.8875591
C	2.8862798	2.5296444	-3.9990339
H	4.1722504	0.8125509	-3.8190394
H	3.6728493	3.2281052	-4.2622989
H	-0.4639327	2.5377324	-3.3490506
C	0.7489171	4.8203128	-5.2415950
O	1.3223877	4.3504456	-4.0712351
O	0.5392428	6.0036113	-5.3364308
C	0.4426129	3.7948254	-6.3101881
H	0.1353061	4.3231255	-7.2123646
H	1.3144937	3.1648504	-6.5119482

H	-0.3628603	3.1326917	-5.9782061
Au	-4.1442810	-5.8775650	1.8573050
Au	-1.2508828	-5.8577650	1.8725050
Au	-2.6865814	-3.3707667	1.8621050
Au	0.1813168	-3.3801667	1.8573050
Au	3.0747150	-3.3603667	1.8725050
Au	1.6390164	-0.8734684	1.8621050
Au	-4.1442810	-0.8828684	1.8573050
Au	-1.2508828	-0.8630684	1.8725050
Au	-2.6865814	1.6239309	1.8621050
Au	0.1813168	1.6145309	1.8573050
Au	3.0747150	1.6343309	1.8725050
Au	1.6390164	-5.8681650	1.8621050
Au	4.5281146	-0.8709684	1.8171050
Au	-5.5868796	-3.3663667	1.8613050
Au	-4.1614810	-4.2779664	4.1460044
Au	-1.2679828	-4.2926664	4.1466044
Au	-2.6740814	-1.7895671	4.2100034
Au	0.1935168	-1.8325671	4.1900044
Au	3.0869150	-1.8472671	4.1907044
Au	1.6809164	0.6557312	4.2540033
Au	-4.1019810	0.7146312	4.2790033
Au	-1.2085828	0.6999312	4.2797033
Au	-2.6145814	3.2030295	4.3430033
Au	0.2530168	3.1600295	4.3231033
Au	3.1464150	3.1453295	4.3237033
Au	1.6214164	-4.3367664	4.1209044

Au	4.5696146	0.6254312	4.1941034
Au	-5.5740796	-1.7509672	4.2244034
Au	3.0310150	3.2914294	-0.4011933
Au	0.1383168	3.2370294	-0.3669934
Au	1.6027164	0.7682311	-0.4500933
Au	-1.2650828	0.7439311	-0.4352933
Au	-4.1578810	0.6896312	-0.4009933
Au	-2.6934814	-1.7790671	-0.4841933
Au	3.0891150	-1.7013672	-0.5278933
Au	0.1963168	-1.7556672	-0.4935933
Au	1.6607164	-4.2244665	-0.5767933
Au	-1.2070828	-4.2488665	-0.5619933
Au	-4.0997810	-4.3031664	-0.5276933
Au	-2.7515814	3.2136295	-0.3575934
Au	-5.5825796	-1.8145671	-0.5096933
Au	4.5027146	0.7981311	-0.4706933

se-m-cf3

Se	-0.6769125	-0.2347695	-3.0417742
C	0.8286182	0.8752750	-3.6139096
C	2.1056562	0.3271785	-3.7879471
C	0.6079310	2.2293412	-3.8910779
C	3.1707088	1.1438772	-4.1710960
H	2.2681593	-0.7286199	-3.5859105
C	1.6780426	3.0325281	-4.2783746
C	2.9660068	2.5013695	-4.4068938
H	4.1658250	0.7191916	-4.2602484

H	3.7952794	3.1472856	-4.6727814
H	-0.3777406	2.6600290	-3.7474403
C	1.4483894	4.4896542	-4.5676389
F	1.2680886	4.7182642	-5.9072230
F	0.3490484	4.9823918	-3.9435855
F	2.5044209	5.2609343	-4.1913930
Au	-4.2414646	-5.6493414	1.4607437
Au	-1.3480664	-5.6295414	1.4759437
Au	-2.7837650	-3.1425431	1.4655437
Au	0.0841332	-3.1519431	1.4607437
Au	2.9775314	-3.1321431	1.4759437
Au	1.5418328	-0.6452448	1.4655437
Au	-4.2414646	-0.6546448	1.4607437
Au	-1.3480664	-0.6348448	1.4759437
Au	-2.7837650	1.8521545	1.4655437
Au	0.0841332	1.8427545	1.4607437
Au	2.9775314	1.8625545	1.4759437
Au	1.5418328	-5.6399414	1.4655437
Au	4.4309310	-0.6427448	1.4205438
Au	-5.6840632	-3.1381431	1.4647437
Au	-4.2586646	-4.0497428	3.7494431
Au	-1.3651664	-4.0644428	3.7500431
Au	-2.7712650	-1.5613435	3.8134421
Au	0.0963332	-1.6043435	3.7934431
Au	2.9897314	-1.6190435	3.7941431
Au	1.5837328	0.8839548	3.8574421
Au	-4.1991646	0.9428548	3.8824421

Au	-1.3057664	0.9281548	3.8831421
Au	-2.7117650	3.4312531	3.9464420
Au	0.1558332	3.3882531	3.9265421
Au	3.0492313	3.3735531	3.9271421
Au	1.5242328	-4.1085428	3.7243431
Au	4.4724309	0.8536548	3.7975421
Au	-5.6712632	-1.5227435	3.8278421
Au	2.9338314	3.5196530	-0.7977546
Au	0.0411332	3.4652531	-0.7635546
Au	1.5055328	0.9964547	-0.8466546
Au	-1.3622664	0.9721548	-0.8318546
Au	-4.2550646	0.9178548	-0.7975546
Au	-2.7906650	-1.5508435	-0.8807546
Au	2.9919314	-1.4731436	-0.9244546
Au	0.0991332	-1.5274435	-0.8901546
Au	1.5635328	-3.9962428	-0.9733546
Au	-1.3042664	-4.0206428	-0.9585546
Au	-4.1969646	-4.0749428	-0.9242546
Au	-2.8487650	3.4418531	-0.7541546
Au	-5.6797632	-1.5863435	-0.9062546
Au	4.4055310	1.0263547	-0.8672546

se-m-cn

Se	-0.5828204	-0.0440658	-3.2301192
C	0.8828229	1.1293592	-3.7849117
C	2.1814052	0.6295483	-3.9550836
C	0.6139606	2.4761385	-4.0474238

C	3.2195336	1.4869262	-4.3270582
H	2.3809771	-0.4215848	-3.7645549
C	1.6603708	3.3297339	-4.4274986
C	2.9731969	2.8377885	-4.5506560
H	4.2287374	1.0972789	-4.4167551
H	3.7783263	3.5147517	-4.8132431
H	-0.3875973	2.8717063	-3.9134079
C	1.3952528	4.7172514	-4.6472197
N	1.1834092	5.8463712	-4.8271142
Au	-4.1803123	-5.5147080	1.2854774
Au	-1.2869141	-5.4949080	1.3006774
Au	-2.7226127	-3.0079097	1.2902774
Au	0.1452854	-3.0173097	1.2854774
Au	3.0386836	-2.9975098	1.3006774
Au	1.6029850	-0.5106114	1.2902774
Au	-4.1803123	-0.5200114	1.2854774
Au	-1.2869141	-0.5002115	1.3006774
Au	-2.7226127	1.9867878	1.2902774
Au	0.1452854	1.9773879	1.2854774
Au	3.0386836	1.9971878	1.3006774
Au	1.6029850	-5.5053080	1.2902774
Au	4.4920832	-0.5081114	1.2452774
Au	-5.6229109	-3.0035097	1.2894774
Au	-4.1975123	-3.9151095	3.5741767
Au	-1.3040141	-3.9298095	3.5747767
Au	-2.7101128	-1.4267102	3.6381757
Au	0.1574854	-1.4697102	3.6181767

Au	3.0508836	-1.4844102	3.6188767
Au	1.6448850	1.0185881	3.6821757
Au	-4.1380123	1.0774881	3.7071757
Au	-1.2446142	1.0627881	3.7078757
Au	-2.6506128	3.5658864	3.7711757
Au	0.2169854	3.5228864	3.7512757
Au	3.1103836	3.5081864	3.7518757
Au	1.5853850	-3.9739095	3.5490767
Au	4.5335832	0.9882881	3.6222757
Au	-5.6101109	-1.3881102	3.6525757
Au	2.9949836	3.6542864	-0.9730210
Au	0.1022855	3.5998864	-0.9388210
Au	1.5666850	1.1310881	-1.0219210
Au	-1.3011141	1.1067881	-1.0071210
Au	-4.1939123	1.0524881	-0.9728210
Au	-2.7295127	-1.4162102	-1.0560210
Au	3.0530836	-1.3385102	-1.0997209
Au	0.1602854	-1.3928102	-1.0654210
Au	1.6246850	-3.8616095	-1.1486209
Au	-1.2431142	-3.8860095	-1.1338209
Au	-4.1358123	-3.9403095	-1.0995209
Au	-2.7876127	3.5764864	-0.9294210
Au	-5.6186109	-1.4517102	-1.0815210
Au	4.4666832	1.1609881	-1.0425210

se-m-no2

Se	-0.6594375	-0.1541732	-3.1827133

C	0.8447102	0.9684234	-3.7251153
C	2.1120917	0.4223835	-3.9797697
C	0.6425233	2.3463947	-3.8591250
C	3.1810397	1.2546994	-4.3225295
H	2.2646442	-0.6476888	-3.8702204
C	1.7270200	3.1485901	-4.1933576
C	3.0039710	2.6329456	-4.4203426
H	4.1653468	0.8242453	-4.4771772
H	3.8230229	3.3054647	-4.6406337
H	-0.3235865	2.7943992	-3.6566351
N	1.5364181	4.6076561	-4.1822265
O	0.4599441	5.0405350	-3.7472618
O	2.4761754	5.3177115	-4.5606995
Au	-4.2213083	-5.5716883	1.3357185
Au	-1.3279101	-5.5518883	1.3509185
Au	-2.7636087	-3.0648900	1.3405185
Au	0.1042895	-3.0742900	1.3357185
Au	2.9976877	-3.0544900	1.3509185
Au	1.5619891	-0.5675917	1.3405185
Au	-4.2213083	-0.5769917	1.3357185
Au	-1.3279101	-0.5571917	1.3509185
Au	-2.7636087	1.9298076	1.3405185
Au	0.1042895	1.9204076	1.3357185
Au	2.9976877	1.9402076	1.3509185
Au	1.5619891	-5.5622883	1.3405185
Au	4.4510872	-0.5650917	1.2955185
Au	-5.6639069	-3.0604900	1.3397185

Au	-4.2385083	-3.9720898	3.6244179
Au	-1.3450101	-3.9867898	3.6250179
Au	-2.7511087	-1.4836905	3.6884169
Au	0.1164895	-1.5266904	3.6684179
Au	3.0098876	-1.5413904	3.6691179
Au	1.6038890	0.9616079	3.7324169
Au	-4.1790083	1.0205078	3.7574169
Au	-1.2856101	1.0058078	3.7581169
Au	-2.6916088	3.5089061	3.8214168
Au	0.1759894	3.4659062	3.8015168
Au	3.0693876	3.4512062	3.8021168
Au	1.5443891	-4.0308897	3.5993179
Au	4.4925872	0.9313079	3.6725169
Au	-5.6511069	-1.4450905	3.7028169
Au	2.9539877	3.5973061	-0.9227798
Au	0.0612895	3.5429061	-0.8885798
Au	1.5256891	1.0741078	-0.9716798
Au	-1.3421101	1.0498078	-0.9568798
Au	-4.2349083	0.9955079	-0.9225798
Au	-2.7705087	-1.4731905	-1.0057798
Au	3.0120876	-1.3954905	-1.0494798
Au	0.1192895	-1.4497905	-1.0151798
Au	1.5836890	-3.9185898	-1.0983798
Au	-1.2841101	-3.9429898	-1.0835798
Au	-4.1768083	-3.9972897	-1.0492798
Au	-2.8286087	3.5195061	-0.8791798
Au	-5.6596069	-1.5086904	-1.0312798

Au	4.4256872	1.1040078	-0.9922798

ORTHO

se-o-nh2

Se	-0.5321324	-0.0195328	-3.2057449
C	0.9813512	1.0982507	-3.7220267
C	2.2634571	0.5303262	-3.8325625
C	0.7937619	2.4661753	-4.0451847
C	3.3680830	1.2988304	-4.1908004
H	2.3788384	-0.5299585	-3.6180563
C	1.9251627	3.2303198	-4.3867713
C	3.1899638	2.6620634	-4.4509454
H	4.3502343	0.8414862	-4.2530927
H	1.7958025	4.2929852	-4.5790449
N	-0.4610965	3.0476142	-4.0298084
H	-0.4802109	4.0546645	-3.9122572
H	-1.1620030	2.5804987	-3.4559823
H	4.0433880	3.2865916	-4.6974114
Au	-4.1545817	-5.4999138	1.3253469
Au	-1.2611835	-5.4801138	1.3405469
Au	-2.6968821	-2.9931155	1.3301469
Au	0.1710160	-3.0025155	1.3253469

Au	3.0644142	-2.9827155	1.3405469
Au	1.6287156	-0.4958172	1.3301469
Au	-4.1545817	-0.5052172	1.3253469
Au	-1.2611835	-0.4854172	1.3405469
Au	-2.6968821	2.0015821	1.3301469
Au	0.1710160	1.9921821	1.3253469
Au	3.0644142	2.0119821	1.3405469
Au	1.6287156	-5.4905138	1.3301469
Au	4.5178138	-0.4933172	1.2851469
Au	-5.5971803	-2.9887155	1.3293469
Au	-4.1717817	-3.9003152	3.6140463
Au	-1.2782835	-3.9150152	3.6146463
Au	-2.6843821	-1.4119159	3.6780453
Au	0.1832160	-1.4549159	3.6580463
Au	3.0766142	-1.4696159	3.6587463
Au	1.6706156	1.0333824	3.7220453
Au	-4.1122817	1.0922824	3.7470453
Au	-1.2188836	1.0775824	3.7477453
Au	-2.6248822	3.5806807	3.8110452
Au	0.2427160	3.5376807	3.7911452
Au	3.1361142	3.5229807	3.7917452
Au	1.6111156	-3.9591152	3.5889463
Au	4.5593138	1.0030824	3.6621453
Au	-5.5843803	-1.3733159	3.6924453
Au	3.0207142	3.6690806	-0.9331514
Au	0.1280161	3.6146807	-0.8989514
Au	1.5924156	1.1458824	-0.9820514

Au	-1.2753835	1.1215824	-0.9672514
Au	-4.1681817	1.0672824	-0.9329514
Au	-2.7037821	-1.4014159	-1.0161514
Au	3.0788142	-1.3237159	-1.0598514
Au	0.1860160	-1.3780159	-1.0255514
Au	1.6504156	-3.8468152	-1.1087514
Au	-1.2173836	-3.8712152	-1.0939514
Au	-4.1100817	-3.9255152	-1.0596514
Au	-2.7618821	3.5912807	-0.8895514
Au	-5.5928803	-1.4369159	-1.0416514
Au	4.4924138	1.1757823	-1.0026514

se-o-och3

Se	-0.5264964	-0.2852685	-2.9913868
C	0.9721057	0.8321998	-3.5636514
C	2.2732912	0.3255834	-3.6599535
C	0.7243274	2.1728656	-3.9289157
C	3.3366051	1.1491928	-4.0381944
H	2.4474855	-0.7196204	-3.4138372
C	1.7927100	2.9995904	-4.2910000
C	3.0916423	2.4888173	-4.3312635
H	4.3430720	0.7449412	-4.0784330
H	1.6196327	4.0455429	-4.5189216
C	-0.8634839	3.9614321	-4.0833366
O	-0.5736726	2.5757392	-3.8648484
H	-1.9316204	4.0616804	-3.8875818

H	-0.6346923	4.2499183	-5.1176562
H	-0.3028813	4.5899711	-3.3793100
H	3.9130888	3.1490554	-4.5924995
Au	-4.0886419	-5.6782950	1.5006725
Au	-1.1952437	-5.6584950	1.5158725
Au	-2.6309423	-3.1714967	1.5054725
Au	0.2369559	-3.1808967	1.5006725
Au	3.1303541	-3.1610967	1.5158725
Au	1.6946555	-0.6741984	1.5054725
Au	-4.0886419	-0.6835984	1.5006725
Au	-1.1952437	-0.6637984	1.5158725
Au	-2.6309423	1.8232009	1.5054725
Au	0.2369559	1.8138009	1.5006725
Au	3.1303541	1.8336009	1.5158725
Au	1.6946555	-5.6688950	1.5054725
Au	4.5837537	-0.6716984	1.4604725
Au	-5.5312405	-3.1670967	1.5046725
Au	-4.1058419	-4.0786964	3.7893719
Au	-1.2123437	-4.0933964	3.7899719
Au	-2.6184423	-1.5902971	3.8533709
Au	0.2491559	-1.6332971	3.8333719
Au	3.1425541	-1.6479971	3.8340719
Au	1.7365555	0.8550012	3.8973709
Au	-4.0463419	0.9139012	3.9223708
Au	-1.1529437	0.8992012	3.9230708
Au	-2.5589423	3.4022995	3.9863708
Au	0.3086559	3.3592995	3.9664708

Au	3.2020541	3.3445995	3.9670708
Au	1.6770555	-4.1374964	3.7642719
Au	4.6252537	0.8247012	3.8374709
Au	-5.5184405	-1.5516971	3.8677709
Au	3.0866541	3.4906994	-0.7578258
Au	0.1939559	3.4362995	-0.7236258
Au	1.6583555	0.9675012	-0.8067258
Au	-1.2094437	0.9432012	-0.7919258
Au	-4.1022419	0.8889012	-0.7576258
Au	-2.6378423	-1.5797971	-0.8408258
Au	3.1447541	-1.5020972	-0.8845258
Au	0.2519559	-1.5563971	-0.8502258
Au	1.7163555	-4.0251964	-0.9334258
Au	-1.1514437	-4.0495964	-0.9186258
Au	-4.0441419	-4.1038964	-0.8843258
Au	-2.6959423	3.4128995	-0.7142259
Au	-5.5269405	-1.6152971	-0.8663258
Au	4.5583537	0.9974011	-0.8273258

se-o-ch3

Se	-0.5555644	-0.1421131	-3.0700863
C	0.8886713	1.0314174	-3.6791975
C	2.2105004	0.5585591	-3.6661032
C	0.6000274	2.3312032	-4.1437941
C	3.2670044	1.3927848	-4.0340413
H	2.4037513	-0.4710933	-3.3724623
C	1.6820008	3.1522311	-4.4858003

C	3.0013668	2.7065870	-4.4173130
H	4.2851113	1.0157356	-4.0005112
H	1.4764218	4.1726369	-4.8013853
H	3.8159582	3.3785987	-4.6703432
C	-0.8064425	2.8497068	-4.2398357
H	-1.4467955	2.1655764	-4.8075099
H	-1.2554515	2.9407904	-3.2392129
H	-0.8261841	3.8345114	-4.7145545
Au	-4.0663204	-5.5490714	1.4433695
Au	-1.1729222	-5.5292714	1.4585695
Au	-2.6086208	-3.0422731	1.4481695
Au	0.2592774	-3.0516731	1.4433695
Au	3.1526756	-3.0318731	1.4585695
Au	1.7169770	-0.5449748	1.4481695
Au	-4.0663204	-0.5543748	1.4433695
Au	-1.1729222	-0.5345748	1.4585695
Au	-2.6086208	1.9524245	1.4481695
Au	0.2592774	1.9430245	1.4433695
Au	3.1526756	1.9628245	1.4585695
Au	1.7169770	-5.5396714	1.4481695
Au	4.6060752	-0.5424748	1.4031695
Au	-5.5089190	-3.0378731	1.4473695
Au	-4.0835204	-3.9494729	3.7320688
Au	-1.1900222	-3.9641728	3.7326688
Au	-2.5961208	-1.4610736	3.7960678
Au	0.2714774	-1.5040735	3.7760688
Au	3.1648756	-1.5187735	3.7767688

Au	1.7588770	0.9842248	3.8400678
Au	-4.0240204	1.0431247	3.8650678
Au	-1.1306222	1.0284248	3.8657678
Au	-2.5366208	3.5315230	3.9290678
Au	0.3309774	3.4885231	3.9091678
Au	3.2243756	3.4738231	3.9097678
Au	1.6993770	-4.0082728	3.7069688
Au	4.6475752	0.9539248	3.7801678
Au	-5.4961190	-1.4224736	3.8104678
Au	3.1089756	3.6199230	-0.8151289
Au	0.2162774	3.5655230	-0.7809289
Au	1.6806770	1.0967247	-0.8640289
Au	-1.1871222	1.0724247	-0.8492289
Au	-4.0799204	1.0181248	-0.8149289
Au	-2.6155208	-1.4505736	-0.8981289
Au	3.1670756	-1.3728736	-0.9418289
Au	0.2742774	-1.4271736	-0.9075289
Au	1.7386770	-3.8959729	-0.9907288
Au	-1.1291222	-3.9203729	-0.9759288
Au	-4.0218204	-3.9746728	-0.9416289
Au	-2.6736208	3.5421230	-0.7715289
Au	-5.5046190	-1.4860735	-0.9236289
Au	4.5806752	1.1266247	-0.8846289

se-o-f

Se	-0.5992513	0.0595928	-3.3140909
C	0.9027118	1.1612161	-3.8956709

C	2.2102835	0.6520938	-3.9774441
C	0.7069915	2.4929768	-4.2710707
C	3.2768121	1.4703744	-4.3493939
H	2.3795918	-0.3914686	-3.7235013
C	1.7564325	3.3278186	-4.6276828
C	3.0526172	2.8141370	-4.6543311
H	4.2808601	1.0586895	-4.3758545
H	1.5462477	4.3680620	-4.8537146
F	-0.5443493	3.0115922	-4.2474384
H	3.8834202	3.4644596	-4.9087360
Au	-4.1641081	-5.3723364	1.2017533
Au	-1.2707099	-5.3525364	1.2169533
Au	-2.7064085	-2.8655381	1.2065533
Au	0.1614896	-2.8749381	1.2017533
Au	3.0548878	-2.8551381	1.2169533
Au	1.6191892	-0.3682398	1.2065533
Au	-4.1641081	-0.3776398	1.2017533
Au	-1.2707099	-0.3578398	1.2169533
Au	-2.7064085	2.1291595	1.2065533
Au	0.1614896	2.1197595	1.2017533
Au	3.0548878	2.1395595	1.2169533
Au	1.6191892	-5.3629364	1.2065533
Au	4.5082874	-0.3657398	1.1615534
Au	-5.6067067	-2.8611381	1.2057533
Au	-4.1813081	-3.7727378	3.4904527
Au	-1.2878099	-3.7874378	3.4910527
Au	-2.6939085	-1.2843385	3.5544517

Au	0.1736896	-1.3273385	3.5344527
Au	3.0670878	-1.3420385	3.5351527
Au	1.6610892	1.1609598	3.5984517
Au	-4.1218081	1.2198598	3.6234517
Au	-1.2284100	1.2051598	3.6241517
Au	-2.6344086	3.7082581	3.6874516
Au	0.2331896	3.6652581	3.6675516
Au	3.1265878	3.6505581	3.6681516
Au	1.6015892	-3.8315378	3.4653527
Au	4.5497874	1.1306598	3.5385517
Au	-5.5939067	-1.2457385	3.5688517
Au	3.0111878	3.7966580	-1.0567450
Au	0.1184897	3.7422581	-1.0225450
Au	1.5828892	1.2734598	-1.1056450
Au	-1.2849099	1.2491598	-1.0908450
Au	-4.1777081	1.1948598	-1.0565450
Au	-2.7133085	-1.2738385	-1.1397450
Au	3.0692878	-1.1961385	-1.1834450
Au	0.1764896	-1.2504385	-1.1491450
Au	1.6408892	-3.7192378	-1.2323450
Au	-1.2269100	-3.7436378	-1.2175450
Au	-4.1196081	-3.7979378	-1.1832450
Au	-2.7714085	3.7188581	-1.0131450
Au	-5.6024067	-1.3093385	-1.1652450
Au	4.4828874	1.3033597	-1.1262450

se-o-cl

Se	-0.6055611	0.0335404	-3.3112422
C	0.8482300	1.1902335	-3.9089421
C	2.1646849	0.6962984	-3.9669299
C	0.6283175	2.5192114	-4.3039886
C	3.2281753	1.5189885	-4.3310058
H	2.3415014	-0.3439362	-3.7051125
C	1.6924997	3.3545234	-4.6456344
C	2.9938388	2.8585705	-4.6465127
H	4.2363531	1.1162732	-4.3407584
H	1.4908662	4.3918758	-4.8903027
Cl	-0.9790136	3.1917521	-4.3159494
H	3.8196892	3.5183663	-4.8926460
Au	-4.1405626	-5.3855498	1.2034683
Au	-1.2471644	-5.3657498	1.2186683
Au	-2.6828630	-2.8787515	1.2082683
Au	0.1850352	-2.8881515	1.2034683
Au	3.0784334	-2.8683515	1.2186683
Au	1.6427348	-0.3814532	1.2082683
Au	-4.1405626	-0.3908532	1.2034683
Au	-1.2471644	-0.3710532	1.2186683
Au	-2.6828630	2.1159461	1.2082683
Au	0.1850352	2.1065461	1.2034683
Au	3.0784334	2.1263461	1.2186683
Au	1.6427348	-5.3761498	1.2082683
Au	4.5318330	-0.3789532	1.1632683
Au	-5.5831612	-2.8743515	1.2074683
Au	-4.1577626	-3.7859512	3.4921676

Au	-1.2642644	-3.8006512	3.4927676
Au	-2.6703630	-1.2975519	3.5561666
Au	0.1972352	-1.3405519	3.5361676
Au	3.0906334	-1.3552519	3.5368676
Au	1.6846348	1.1477464	3.6001666
Au	-4.0982626	1.2066464	3.6251666
Au	-1.2048644	1.1919464	3.6258666
Au	-2.6108630	3.6950447	3.6891666
Au	0.2567352	3.6520447	3.6692666
Au	3.1501334	3.6373447	3.6698666
Au	1.6251348	-3.8447512	3.4670677
Au	4.5733330	1.1174464	3.5402666
Au	-5.5703612	-1.2589519	3.5705666
Au	3.0347334	3.7834446	-1.0550301
Au	0.1420352	3.7290447	-1.0208301
Au	1.6064348	1.2602464	-1.1039301
Au	-1.2613644	1.2359464	-1.0891301
Au	-4.1541626	1.1816464	-1.0548301
Au	-2.6897630	-1.2870519	-1.1380301
Au	3.0928334	-1.2093520	-1.1817300
Au	0.2000352	-1.2636519	-1.1474300
Au	1.6644348	-3.7324512	-1.2306300
Au	-1.2033644	-3.7568512	-1.2158300
Au	-4.0960626	-3.8111512	-1.1815300
Au	-2.7478630	3.7056447	-1.0114301
Au	-5.5788612	-1.3225519	-1.1635300
Au	4.5064330	1.2901463	-1.1245301

se-o-ococh3

Se	-0.7216236	0.2476884	-2.7603822
C	0.7921253	1.3182427	-3.3674662
C	2.0704922	0.7757693	-3.5784116
C	0.6084277	2.6872902	-3.6212829
C	3.1473253	1.5810612	-3.9451392
C	1.6749137	3.4916401	-4.0058206
H	-0.3729525	3.1206535	-3.4481285
C	2.9514965	2.9434745	-4.1534972
H	4.1297693	1.1275653	-4.0286735
H	1.5152253	4.5554786	-4.1522383
H	3.7946685	3.5752641	-4.4136576
C	1.2666828	-1.3258203	-5.3154383
H	1.8299961	-0.5724464	-5.8736548
C	1.9340486	-1.6395235	-4.0039697
H	0.2664075	-0.9218949	-5.1206357
H	1.1779458	-2.2495670	-5.8862696
O	2.1488218	-2.7416438	-3.5663509
O	2.3661241	-0.5539931	-3.2519539
Au	-4.3480810	-5.1802023	1.7566920
Au	-1.4546818	-5.1604023	1.7718920
Au	-2.8903814	-2.6734040	1.7614920
Au	-0.0224832	-2.6828040	1.7566920
Au	2.8709150	-2.6630040	1.7718920
Au	1.4352164	-0.1761057	1.7614920
Au	-4.3480810	-0.1855057	1.7566920

Au	-1.4546818	-0.1657057	1.7718920
Au	-2.8903814	2.3212936	1.7614920
Au	-0.0224832	2.3118936	1.7566920
Au	2.8709150	2.3316936	1.7718920
Au	1.4352164	-5.1708023	1.7614920
Au	4.3243146	-0.1736057	1.7164920
Au	-5.7906796	-2.6690040	1.7606920
Au	-4.3652810	-3.5806038	4.0453903
Au	-1.4717818	-3.5953038	4.0459903
Au	-2.8778814	-1.0922045	4.1093903
Au	-0.0102832	-1.1352045	4.0893903
Au	2.8831150	-1.1499045	4.0900903
Au	1.4771164	1.3530938	4.1533903
Au	-4.3057810	1.4119938	4.1783903
Au	-1.4123818	1.3972938	4.1790903
Au	-2.8183814	3.9003921	4.2423903
Au	0.0492168	3.8573921	4.2224903
Au	2.9426150	3.8426921	4.2230903
Au	1.4176164	-3.6394038	4.0202903
Au	4.3658146	1.3227938	4.0934903
Au	-5.7778796	-1.0536045	4.1237903
Au	2.8272150	3.9887921	-0.5018074
Au	-0.0654832	3.9343921	-0.4676074
Au	1.3989164	1.4655938	-0.5507074
Au	-1.4688818	1.4412938	-0.5359074
Au	-4.3616810	1.3869938	-0.5016074
Au	-2.8972814	-1.0817045	-0.5848074

Au	2.8853150	-1.0040045	-0.6285074
Au	-0.0074832	-1.0583045	-0.5942074
Au	1.4569164	-3.5271038	-0.6774074
Au	-1.4108818	-3.5515038	-0.6626074
Au	-4.3035810	-3.6058038	-0.6283074
Au	-2.9553814	3.9109921	-0.4582074
Au	-5.7863796	-1.1172045	-0.6103074
Au	4.2989146	1.4954938	-0.5713074

se-o-cf3

Se	-0.5423950	0.0815134	-3.0643069
C	0.6575440	1.4972709	-3.7231604
C	1.9845446	1.3468153	-4.1601926
C	0.1020720	2.7895032	-3.7236717
C	2.7560438	2.4819473	-4.4498667
C	0.8627532	3.9056113	-4.0480641
C	2.2142286	3.7583751	-4.3680937
H	3.7947874	2.3480113	-4.7333059
H	0.4072394	4.8908444	-4.0098269
H	2.8341443	4.6254817	-4.5709953
H	-0.9358072	2.9090846	-3.4244440
C	2.6543529	0.0249761	-4.4519206
F	2.6480767	-0.2154928	-5.8036272
F	3.9672519	0.0268792	-4.0817690
F	2.0869552	-1.0531342	-3.8738324
Au	-4.2269718	-5.5134831	1.4708684
Au	-1.3335736	-5.4936831	1.4860684

Au	-2.7692722	-3.0066848	1.4756684
Au	0.0986260	-3.0160848	1.4708684
Au	2.9920242	-2.9962848	1.4860684
Au	1.5563256	-0.5093865	1.4756684
Au	-4.2269718	-0.5187865	1.4708684
Au	-1.3335736	-0.4989865	1.4860684
Au	-2.7692722	1.9880128	1.4756684
Au	0.0986260	1.9786128	1.4708684
Au	2.9920242	1.9984128	1.4860684
Au	1.5563256	-5.5040831	1.4756684
Au	4.4454238	-0.5068865	1.4306684
Au	-5.6695704	-3.0022848	1.4748684
Au	-4.2441718	-3.9138846	3.7595678
Au	-1.3506736	-3.9285846	3.7601678
Au	-2.7567722	-1.4254853	3.8235667
Au	0.1108260	-1.4684853	3.8035677
Au	3.0042242	-1.4831853	3.8042677
Au	1.5982256	1.0198130	3.8675667
Au	-4.1846718	1.0787130	3.8925667
Au	-1.2912736	1.0640130	3.8932667
Au	-2.6972722	3.5671113	3.9565667
Au	0.1703260	3.5241113	3.9366667
Au	3.0637242	3.5094113	3.9372667
Au	1.5387256	-3.9726846	3.7344678
Au	4.4869238	0.9895130	3.8076667
Au	-5.6567704	-1.3868853	3.8379667
Au	2.9483242	3.6555113	-0.7876300

Au	0.0556260	3.6011113	-0.7534300
Au	1.5200256	1.1323130	-0.8365299
Au	-1.3477736	1.1080130	-0.8217300
Au	-4.2405718	1.0537130	-0.7874300
Au	-2.7761722	-1.4149853	-0.8706299
Au	3.0064242	-1.3372853	-0.9143299
Au	0.1136260	-1.3915853	-0.8800299
Au	1.5780256	-3.8603846	-0.9632299
Au	-1.2897736	-3.8847846	-0.9484299
Au	-4.1824718	-3.9390846	-0.9141299
Au	-2.8342722	3.5777113	-0.7440300
Au	-5.6652704	-1.4504853	-0.8961299
Au	4.4200238	1.1622130	-0.8571299

se-o-cn

Se	-0.5902992	-0.0657079	-3.2573163
C	0.7719576	1.1888919	-3.8566592
C	2.1181838	0.8021194	-3.9222044
C	0.4270608	2.5147156	-4.2014995
C	3.1064501	1.7259408	-4.2630947
H	2.3872347	-0.2226173	-3.6817859
C	1.4329316	3.4398060	-4.5363464
C	2.7672671	3.0509276	-4.5500404
H	4.1460269	1.4125393	-4.2705032
H	1.1501797	4.4645263	-4.7543295
H	3.5403996	3.7769723	-4.7790935
C	-0.9252421	2.9676737	-4.1460669

N	-2.0102152	3.3865661	-4.0906074
Au	-4.0563036	-5.4901577	1.2759903
Au	-1.1629054	-5.4703577	1.2911903
Au	-2.5986040	-2.9833594	1.2807903
Au	0.2692942	-2.9927594	1.2759903
Au	3.1626924	-2.9729594	1.2911903
Au	1.7269938	-0.4860611	1.2807903
Au	-4.0563036	-0.4954611	1.2759903
Au	-1.1629054	-0.4756611	1.2911903
Au	-2.5986040	2.0113382	1.2807903
Au	0.2692942	2.0019382	1.2759903
Au	3.1626924	2.0217382	1.2911903
Au	1.7269938	-5.4807577	1.2807903
Au	4.6160920	-0.4835611	1.2357903
Au	-5.4989022	-2.9789594	1.2799903
Au	-4.0735036	-3.8905592	3.5646896
Au	-1.1800054	-3.9052591	3.5652896
Au	-2.5861040	-1.4021598	3.6286886
Au	0.2814942	-1.4451598	3.6086896
Au	3.1748924	-1.4598598	3.6093896
Au	1.7688938	1.0431385	3.6726886
Au	-4.0140036	1.1020384	3.6976886
Au	-1.1206054	1.0873385	3.6983886
Au	-2.5266040	3.5904367	3.7616886
Au	0.3409942	3.5474368	3.7417886
Au	3.2343924	3.5327368	3.7423886
Au	1.7093938	-3.9493591	3.5395897

Au	4.6575920	1.0128385	3.6127886
Au	-5.4861022	-1.3635599	3.6430886
Au	3.1189924	3.6788367	-0.9825081
Au	0.2262942	3.6244367	-0.9483081
Au	1.6906938	1.1556384	-1.0314081
Au	-1.1771054	1.1313384	-1.0166081
Au	-4.0699036	1.0770385	-0.9823081
Au	-2.6055040	-1.3916599	-1.0655081
Au	3.1770924	-1.3139599	-1.1092080
Au	0.2842942	-1.3682599	-1.0749080
Au	1.7486938	-3.8370592	-1.1581080
Au	-1.1191054	-3.8614592	-1.1433080
Au	-4.0118036	-3.9157591	-1.1090080
Au	-2.6636040	3.6010367	-0.9389081
Au	-5.4946022	-1.4271598	-1.0910080
Au	4.5906920	1.1855384	-1.0520081

se-o-no2

Se	-0.6172883	-0.1392302	-3.1660226
C	0.8085627	1.0292575	-3.8030083
C	2.0969946	0.4788040	-3.9413278
C	0.6715657	2.4085696	-4.0488908
C	3.1907417	1.2715813	-4.2709850
H	2.2296298	-0.5820727	-3.7482243
C	1.7777328	3.2207383	-4.3203354
C	3.0371215	2.6525950	-4.4404708
H	4.1720881	0.8143675	-4.3536314

H	1.6200655	4.2881488	-4.4187986
H	3.8971295	3.2803522	-4.6480476
N	-0.6159129	3.0736203	-3.9051456
O	-1.6509432	2.4028800	-4.0199142
O	-0.6054456	4.2810286	-3.5759708
Au	-4.0964296	-5.4910116	1.3317170
Au	-1.2030314	-5.4712116	1.3469170
Au	-2.6387300	-2.9842133	1.3365170
Au	0.2291682	-2.9936133	1.3317170
Au	3.1225664	-2.9738133	1.3469170
Au	1.6868678	-0.4869150	1.3365170
Au	-4.0964296	-0.4963150	1.3317170
Au	-1.2030314	-0.4765150	1.3469170
Au	-2.6387300	2.0104843	1.3365170
Au	0.2291682	2.0010843	1.3317170
Au	3.1225664	2.0208843	1.3469170
Au	1.6868678	-5.4816116	1.3365170
Au	4.5759660	-0.4844150	1.2915170
Au	-5.5390282	-2.9798133	1.3357170
Au	-4.1136296	-3.8914131	3.6204163
Au	-1.2201314	-3.9061131	3.6210163
Au	-2.6262300	-1.4030138	3.6844153
Au	0.2413682	-1.4460138	3.6644163
Au	3.1347664	-1.4607138	3.6651163
Au	1.7287678	1.0422845	3.7284153
Au	-4.0541296	1.1011845	3.7534153
Au	-1.1607314	1.0864845	3.7541153

Au	-2.5667300	3.5895828	3.8174153
Au	0.3008682	3.5465828	3.7975153
Au	3.1942664	3.5318828	3.7981153
Au	1.6692678	-3.9502131	3.5953163
Au	4.6174660	1.0119845	3.6685153
Au	-5.5262282	-1.3644138	3.6988153
Au	3.0788664	3.6779828	-0.9267814
Au	0.1861682	3.6235828	-0.8925814
Au	1.6505678	1.1547845	-0.9756814
Au	-1.2172314	1.1304845	-0.9608814
Au	-4.1100296	1.0761845	-0.9265814
Au	-2.6456300	-1.3925138	-1.0097814
Au	3.1369664	-1.3148138	-1.0534814
Au	0.2441682	-1.3691138	-1.0191814
Au	1.7085678	-3.8379131	-1.1023813
Au	-1.1592314	-3.8623131	-1.0875813
Au	-4.0519296	-3.9166131	-1.0532814
Au	-2.7037300	3.6001828	-0.8831814
Au	-5.5347282	-1.4280138	-1.0352814
Au	4.5505660	1.1846845	-0.9962814

Coordinates from the Tellurium-based Ligands

META

te-m-nh2

Te	-0.9889575	-0.0191644	-3.2944465
C	1.0292471	0.5955803	-3.8138439
C	1.9908320	-0.4053243	-4.0135656
C	1.3649784	1.9459102	-3.9049632
C	3.3058601	-0.0212824	-4.2823136
H	1.7238306	-1.4540372	-3.9308871
C	2.6940830	2.3254334	-4.1821620
C	3.6641454	1.3237696	-4.3541179
H	4.0690712	-0.7844258	-4.4091016
H	4.6971883	1.6032692	-4.5441131
H	0.6153770	2.7140863	-3.7299101
N	3.0186682	3.6743151	-4.2847416
H	2.3887771	4.2906408	-3.7746901
H	3.9852483	3.8899867	-4.0617662
Au	-4.4117667	-5.2868588	1.3070186
Au	-1.5183685	-5.2670588	1.3222186
Au	-2.9540671	-2.7800605	1.3118186
Au	-0.0861689	-2.7894605	1.3070186
Au	2.8072293	-2.7696605	1.3222186
Au	1.3715307	-0.2827622	1.3118186
Au	-4.4117667	-0.2921622	1.3070186
Au	-1.5183685	-0.2723622	1.3222186
Au	-2.9540671	2.2146371	1.3118186
Au	-0.0861689	2.2052371	1.3070186

Au	2.8072293	2.2250371	1.3222186
Au	1.3715307	-5.2774588	1.3118186
Au	4.2606289	-0.2802622	1.2668186
Au	-5.8543653	-2.7756605	1.3110186
Au	-4.4289667	-3.6872602	3.5957180
Au	-1.5354685	-3.7019602	3.5963180
Au	-2.9415671	-1.1988609	3.6597169
Au	-0.0739689	-1.2418609	3.6397179
Au	2.8194293	-1.2565609	3.6404179
Au	1.4134307	1.2464374	3.7037169
Au	-4.3694667	1.3053374	3.7287169
Au	-1.4760685	1.2906374	3.7294169
Au	-2.8820671	3.7937357	3.7927169
Au	-0.0144689	3.7507357	3.7728169
Au	2.8789292	3.7360357	3.7734169
Au	1.3539307	-3.7460602	3.5706180
Au	4.3021288	1.2161374	3.6438169
Au	-5.8415653	-1.1602609	3.6741169
Au	2.7635293	3.8821357	-0.9514798
Au	-0.1291689	3.8277357	-0.9172798
Au	1.3352307	1.3589374	-1.0003798
Au	-1.5325685	1.3346374	-0.9855798
Au	-4.4253667	1.2803374	-0.9512798
Au	-2.9609671	-1.1883609	-1.0344797
Au	2.8216293	-1.1106609	-1.0781797
Au	-0.0711689	-1.1649609	-1.0438797
Au	1.3932307	-3.6337602	-1.1270797

Au	-1.4745685	-3.6581602	-1.1122797
Au	-4.3672667	-3.7124602	-1.0779797
Au	-3.0190671	3.8043357	-0.9078798
Au	-5.8500653	-1.2238609	-1.0599797
Au	4.2352289	1.3888374	-1.0209797

te-m-och3

Te	-0.6520061	-0.4120627	-3.1168370
C	1.0204586	0.8690791	-3.6484117
C	2.3067045	0.3449101	-3.8023513
C	0.7659040	2.2300580	-3.8592381
C	3.3552133	1.2121273	-4.1342560
H	2.4976403	-0.7104856	-3.6312776
C	1.8296267	3.0844128	-4.1670834
C	3.1282140	2.5708713	-4.3077395
H	4.3638261	0.8192734	-4.2261828
H	3.9403040	3.2566010	-4.5252544
H	-0.2365148	2.6158326	-3.7095611
C	0.4224231	5.0080041	-4.0102792
O	1.6997614	4.4360194	-4.3209270
H	0.5527319	6.0859207	-4.1098614
H	0.1276971	4.7583514	-2.9776627
H	-0.3492323	4.6566186	-4.7075155
Au	-4.2098161	-5.7851111	1.4893825
Au	-1.3164179	-5.7653111	1.5045825
Au	-2.7521165	-3.2783128	1.4941825
Au	0.1157817	-3.2877128	1.4893825

Au	3.0091799	-3.2679128	1.5045825
Au	1.5734813	-0.7810145	1.4941825
Au	-4.2098161	-0.7904145	1.4893825
Au	-1.3164179	-0.7706145	1.5045825
Au	-2.7521165	1.7163848	1.4941825
Au	0.1157817	1.7069848	1.4893825
Au	3.0091799	1.7267848	1.5045825
Au	1.5734813	-5.7757111	1.4941825
Au	4.4625795	-0.7785145	1.4491825
Au	-5.6524147	-3.2739128	1.4933825
Au	-4.2270161	-4.1855125	3.7780819
Au	-1.3335179	-4.2002125	3.7786819
Au	-2.7396165	-1.6971132	3.8420809
Au	0.1279817	-1.7401132	3.8220819
Au	3.0213799	-1.7548132	3.8227819
Au	1.6153813	0.7481851	3.8860809
Au	-4.1675161	0.8070851	3.9110808
Au	-1.2741179	0.7923851	3.9117808
Au	-2.6801165	3.2954834	3.9750808
Au	0.1874817	3.2524834	3.9551808
Au	3.0808799	3.2377834	3.9557808
Au	1.5558813	-4.2443125	3.7529819
Au	4.5040795	0.7178851	3.8261809
Au	-5.6396147	-1.6585132	3.8564809
Au	2.9654799	3.3838834	-0.7691158
Au	0.0727817	3.3294834	-0.7349158
Au	1.5371813	0.8606851	-0.8180158

Au	-1.3306179	0.8363851	-0.8032158
Au	-4.2234161	0.7820851	-0.7689158
Au	-2.7590165	-1.6866132	-0.8521158
Au	3.0235799	-1.6089132	-0.8958158
Au	0.1307817	-1.6632132	-0.8615158
Au	1.5951813	-4.1320125	-0.9447158
Au	-1.2726179	-4.1564125	-0.9299158
Au	-4.1653161	-4.2107125	-0.8956158
Au	-2.8171165	3.3060834	-0.7255158
Au	-5.6481147	-1.7221132	-0.8776158
Au	4.4371795	0.8905851	-0.8386158

te-m-ch3

Te	-0.7353510	-0.3161828	-3.1650702
C	0.9467566	0.9429333	-3.7149756
C	2.2290882	0.4035908	-3.8754699
C	0.7244043	2.3067179	-3.9250760
C	3.2871211	1.2549803	-4.1981106
H	2.4051529	-0.6562127	-3.7115007
C	1.7836362	3.1661560	-4.2383035
C	3.0680160	2.6227545	-4.3653008
H	4.2900204	0.8481704	-4.2937360
H	3.9065285	3.2792106	-4.5831635
H	-0.2726305	2.7187494	-3.7822234
C	1.5428937	4.6509838	-4.3327069
H	0.5909705	4.8708690	-4.8270418
H	1.4963383	5.0796079	-3.3215681

H	2.3468566	5.1555823	-4.8772196
Au	-4.2773564	-5.6780609	1.4407959
Au	-1.3839582	-5.6582609	1.4559959
Au	-2.8196568	-3.1712626	1.4455959
Au	0.0482414	-3.1806626	1.4407959
Au	2.9416396	-3.1608626	1.4559959
Au	1.5059410	-0.6739643	1.4455959
Au	-4.2773564	-0.6833643	1.4407959
Au	-1.3839582	-0.6635643	1.4559959
Au	-2.8196568	1.8234350	1.4455959
Au	0.0482414	1.8140350	1.4407959
Au	2.9416396	1.8338350	1.4559959
Au	1.5059410	-5.6686609	1.4455959
Au	4.3950392	-0.6714643	1.4005959
Au	-5.7199550	-3.1668626	1.4447959
Au	-4.2945564	-4.0784623	3.7294953
Au	-1.4010582	-4.0931623	3.7300953
Au	-2.8071568	-1.5900630	3.7934943
Au	0.0604414	-1.6330630	3.7734953
Au	2.9538396	-1.6477630	3.7741953
Au	1.5478410	0.8552353	3.8374942
Au	-4.2350564	0.9141353	3.8624942
Au	-1.3416582	0.8994353	3.8631942
Au	-2.7476568	3.4025336	3.9264942
Au	0.1199414	3.3595336	3.9065942
Au	3.0133396	3.3448336	3.9071942
Au	1.4883410	-4.1372623	3.7043953

Au	4.4365392	0.8249353	3.7775943
Au	-5.7071550	-1.5514630	3.8078943
Au	2.8979396	3.4909336	-0.8177024
Au	0.0052414	3.4365336	-0.7835025
Au	1.4696410	0.9677353	-0.8666024
Au	-1.3981582	0.9434353	-0.8518024
Au	-4.2909564	0.8891353	-0.8175024
Au	-2.8265568	-1.5795630	-0.9007024
Au	2.9560396	-1.5018630	-0.9444024
Au	0.0632414	-1.5561630	-0.9101024
Au	1.5276410	-4.0249623	-0.9933024
Au	-1.3401582	-4.0493623	-0.9785024
Au	-4.2328564	-4.1036623	-0.9442024
Au	-2.8846568	3.4131336	-0.7741025
Au	-5.7156550	-1.6150630	-0.9262024
Au	4.3696392	0.9976353	-0.8872024

te-m-h

Te	-0.6870543	-0.0482420	-3.4014040
C	0.9688509	1.2354324	-3.9762855
C	2.2592148	0.7125087	-4.1221360
C	0.7240178	2.5887481	-4.2360935
C	3.3101190	1.5548781	-4.4924405
H	2.4484065	-0.3374801	-3.9124769
C	1.7793183	3.4208052	-4.6083501
C	3.0724059	2.9085608	-4.7308306
H	4.3154434	1.1505619	-4.5704359

H	1.5928690	4.4785362	-4.7732513
H	3.8949675	3.5662871	-4.9953159
H	-0.2726423	2.9995679	-4.0970979
Au	-4.1674123	-5.3865679	1.1950580
Au	-1.2740141	-5.3667679	1.2102580
Au	-2.7097127	-2.8797696	1.1998580
Au	0.1581855	-2.8891696	1.1950580
Au	3.0515837	-2.8693696	1.2102580
Au	1.6158851	-0.3824713	1.1998580
Au	-4.1674123	-0.3918713	1.1950580
Au	-1.2740141	-0.3720713	1.2102580
Au	-2.7097127	2.1149280	1.1998580
Au	0.1581855	2.1055280	1.1950580
Au	3.0515837	2.1253280	1.2102580
Au	1.6158851	-5.3771679	1.1998580
Au	4.5049833	-0.3799713	1.1548580
Au	-5.6100108	-2.8753696	1.1990580
Au	-4.1846122	-3.7869694	3.4837573
Au	-1.2911141	-3.8016694	3.4843573
Au	-2.6972127	-1.2985701	3.5477563
Au	0.1703855	-1.3415701	3.5277573
Au	3.0637837	-1.3562701	3.5284573
Au	1.6577851	1.1467282	3.5917563
Au	-4.1251123	1.2056282	3.6167563
Au	-1.2317141	1.1909282	3.6174563
Au	-2.6377127	3.6940265	3.6807563
Au	0.2298855	3.6510265	3.6608563

Au	3.1232837	3.6363265	3.6614563
Au	1.5982851	-3.8457694	3.4586573
Au	4.5464833	1.1164282	3.5318563
Au	-5.5972109	-1.2599701	3.5621563
Au	3.0078837	3.7824265	-1.0634404
Au	0.1151855	3.7280265	-1.0292404
Au	1.5795851	1.2592282	-1.1123404
Au	-1.2882141	1.2349282	-1.0975404
Au	-4.1810122	1.1806282	-1.0632404
Au	-2.7166127	-1.2880701	-1.1464404
Au	3.0659837	-1.2103701	-1.1901404
Au	0.1731855	-1.2646701	-1.1558404
Au	1.6375851	-3.7334694	-1.2390404
Au	-1.2302141	-3.7578694	-1.2242404
Au	-4.1229123	-3.8121694	-1.1899404
Au	-2.7747126	3.7046265	-1.0198404
Au	-5.6057108	-1.3235701	-1.1719404
Au	4.4795833	1.2891282	-1.1329404

te-m-f

Te	-0.6905395	-0.0346280	-3.3970252
C	1.0041799	1.2025037	-3.9668233
C	2.2863390	0.6503133	-4.0814447
C	0.7923535	2.5600834	-4.2309419
C	3.3686925	1.4721642	-4.4036342
H	2.4451237	-0.4051818	-3.8778832
C	1.8908866	3.3413233	-4.5585850

C	3.1821472	2.8342329	-4.6372720
H	4.3676362	1.0483776	-4.4495220
H	4.0101602	3.4969540	-4.8622336
H	-0.1854404	3.0202469	-4.1263752
F	1.6969066	4.6685418	-4.7839268
Au	-4.1954095	-5.3810597	1.2064733
Au	-1.3020113	-5.3612597	1.2216733
Au	-2.7377099	-2.8742614	1.2112733
Au	0.1301883	-2.8836614	1.2064733
Au	3.0235865	-2.8638614	1.2216733
Au	1.5878879	-0.3769631	1.2112733
Au	-4.1954095	-0.3863631	1.2064733
Au	-1.3020113	-0.3665631	1.2216733
Au	-2.7377099	2.1204362	1.2112733
Au	0.1301883	2.1110362	1.2064733
Au	3.0235865	2.1308362	1.2216733
Au	1.5878879	-5.3716597	1.2112733
Au	4.4769861	-0.3744631	1.1662733
Au	-5.6380081	-2.8698614	1.2104733
Au	-4.2126095	-3.7814611	3.4951726
Au	-1.3191113	-3.7961611	3.4957726
Au	-2.7252099	-1.2930618	3.5591716
Au	0.1423883	-1.3360618	3.5391726
Au	3.0357865	-1.3507618	3.5398726
Au	1.6297879	1.1522365	3.6031716
Au	-4.1531095	1.2111364	3.6281716
Au	-1.2597113	1.1964365	3.6288716

Au	-2.6657099	3.6995347	3.6921716
Au	0.2018883	3.6565348	3.6722716
Au	3.0952864	3.6418348	3.6728716
Au	1.5702879	-3.8402611	3.4700726
Au	4.5184860	1.1219365	3.5432716
Au	-5.6252081	-1.2544619	3.5735716
Au	2.9798865	3.7879347	-1.0520251
Au	0.0871883	3.7335347	-1.0178251
Au	1.5515879	1.2647364	-1.1009251
Au	-1.3162113	1.2404364	-1.0861251
Au	-4.2090095	1.1861365	-1.0518251
Au	-2.7446099	-1.2825619	-1.1350251
Au	3.0379865	-1.2048619	-1.1787250
Au	0.1451883	-1.2591619	-1.1444251
Au	1.6095879	-3.7279612	-1.2276250
Au	-1.2582113	-3.7523612	-1.2128250
Au	-4.1509095	-3.8066611	-1.1785250
Au	-2.8027099	3.7101347	-1.0084251
Au	-5.6337081	-1.3180618	-1.1605251
Au	4.4515861	1.2946364	-1.1215251

te-m-cl

Te	-0.6700275	-0.0386890	-3.4066791
C	1.0164926	1.2134512	-3.9745202
C	2.2926845	0.6645611	-4.1492947
C	0.8044693	2.5843222	-4.1562964
C	3.3664487	1.5022784	-4.4564294

H	2.4555478	-0.3988727	-3.9982170
C	1.8945746	3.3979639	-4.4529527
C	3.1802425	2.8772542	-4.5964602
H	4.3636115	1.0830612	-4.5552135
H	4.0157257	3.5387132	-4.7951190
H	-0.1761589	3.0246899	-4.0039608
Cl	1.6475074	5.1263719	-4.5628847
Au	-4.1959701	-5.3982685	1.2003009
Au	-1.3025720	-5.3784685	1.2155009
Au	-2.7382706	-2.8914702	1.2051009
Au	0.1296276	-2.9008702	1.2003009
Au	3.0230258	-2.8810702	1.2155009
Au	1.5873272	-0.3941719	1.2051009
Au	-4.1959701	-0.4035719	1.2003009
Au	-1.3025720	-0.3837719	1.2155009
Au	-2.7382706	2.1032274	1.2051009
Au	0.1296276	2.0938274	1.2003009
Au	3.0230258	2.1136274	1.2155009
Au	1.5873272	-5.3888685	1.2051009
Au	4.4764254	-0.3916719	1.1601009
Au	-5.6385687	-2.8870702	1.2043009
Au	-4.2131701	-3.7986700	3.4890002
Au	-1.3196719	-3.8133700	3.4896002
Au	-2.7257706	-1.3102707	3.5529992
Au	0.1418276	-1.3532706	3.5330002
Au	3.0352258	-1.3679706	3.5337002
Au	1.6292272	1.1350277	3.5969992

Au	-4.1536702	1.1939276	3.6219992
Au	-1.2602720	1.1792276	3.6226992
Au	-2.6662706	3.6823259	3.6859992
Au	0.2013276	3.6393260	3.6660992
Au	3.0947258	3.6246260	3.6666992
Au	1.5697272	-3.8574699	3.4639002
Au	4.5179254	1.1047277	3.5370992
Au	-5.6257687	-1.2716707	3.5673992
Au	2.9793258	3.7707259	-1.0581975
Au	0.0866277	3.7163259	-1.0239975
Au	1.5510272	1.2475276	-1.1070975
Au	-1.3167719	1.2232276	-1.0922975
Au	-4.2095701	1.1689276	-1.0579975
Au	-2.7451705	-1.2997707	-1.1411975
Au	3.0374258	-1.2220707	-1.1848975
Au	0.1446276	-1.2763707	-1.1505975
Au	1.6090272	-3.7451700	-1.2337975
Au	-1.2587720	-3.7695700	-1.2189975
Au	-4.1514702	-3.8238700	-1.1846975
Au	-2.8032705	3.6929259	-1.0145975
Au	-5.6342687	-1.3352706	-1.1666975
Au	4.4510254	1.2774276	-1.1276975

te-m-ococh3

Te	-0.7104566	-0.5281718	-2.7266696
C	0.9174142	0.7998957	-3.2903915
C	2.2309986	0.3296408	-3.4126356

C	0.6259186	2.1453022	-3.5364955
C	3.2573333	1.2213202	-3.7333743
H	2.4584136	-0.7134390	-3.2114633
C	1.6609666	3.0091914	-3.8807211
C	2.9808281	2.5686311	-3.9618617
H	4.2818209	0.8636557	-3.7792297
H	3.7684972	3.2805972	-4.1843032
H	-0.3791343	2.5363457	-3.4043055
C	0.8374429	4.8627731	-5.2063898
O	1.4017154	4.3758923	-4.0393144
O	0.6056229	6.0436639	-5.2768895
C	0.5719407	3.8588070	-6.3060561
H	0.2817190	4.4050260	-7.2030623
H	1.4568113	3.2441498	-6.4984030
H	-0.2340794	3.1794541	-6.0122311
Au	-4.2393989	-5.8959571	1.8800724
Au	-1.3460007	-5.8761571	1.8952724
Au	-2.7816993	-3.3891588	1.8848724
Au	0.0861989	-3.3985588	1.8800724
Au	2.9795971	-3.3787588	1.8952724
Au	1.5438985	-0.8918605	1.8848724
Au	-4.2393989	-0.9012605	1.8800724
Au	-1.3460007	-0.8814605	1.8952724
Au	-2.7816993	1.6055388	1.8848724
Au	0.0861989	1.5961388	1.8800724
Au	2.9795971	1.6159388	1.8952724
Au	1.5438985	-5.8865571	1.8848724

Au	4.4329967	-0.8893605	1.8398724
Au	-5.6819975	-3.3847588	1.8840724
Au	-4.2565989	-4.2963586	4.1687718
Au	-1.3631007	-4.3110586	4.1693718
Au	-2.7691993	-1.8079593	4.2327708
Au	0.0983989	-1.8509593	4.2127718
Au	2.9917971	-1.8656593	4.2134718
Au	1.5857985	0.6373390	4.2767708
Au	-4.1970989	0.6962390	4.3017707
Au	-1.3037007	0.6815390	4.3024707
Au	-2.7096993	3.1846373	4.3657707
Au	0.1578989	3.1416373	4.3458707
Au	3.0512971	3.1269373	4.3464707
Au	1.5262985	-4.3551586	4.1436718
Au	4.4744967	0.6070391	4.2168708
Au	-5.6691975	-1.7693593	4.2471708
Au	2.9358971	3.2730373	-0.3784259
Au	0.0431989	3.2186373	-0.3442260
Au	1.5075985	0.7498390	-0.4273259
Au	-1.3602007	0.7255390	-0.4125259
Au	-4.2529989	0.6712390	-0.3782259
Au	-2.7885993	-1.7974593	-0.4614259
Au	2.9939971	-1.7197593	-0.5051259
Au	0.1011989	-1.7740593	-0.4708259
Au	1.5655985	-4.2428586	-0.5540259
Au	-1.3022007	-4.2672586	-0.5392259
Au	-4.1948989	-4.3215586	-0.5049259

Au	-2.8466993	3.1952373	-0.3348260
Au	-5.6776975	-1.8329593	-0.4869259
Au	4.4075967	0.7797390	-0.4479259

te-m-cf3

Te	-0.8263253	-0.3665230	-3.1283079
C	0.8626760	0.8731721	-3.7124068
C	2.1419293	0.3273507	-3.8685930
C	0.6411894	2.2293235	-3.9708244
C	3.2092686	1.1476363	-4.2405993
H	2.3106010	-0.7246823	-3.6579303
C	1.7142696	3.0370143	-4.3488251
C	2.9996359	2.5039767	-4.4770851
H	4.2077133	0.7282754	-4.3170637
H	3.8296135	3.1522730	-4.7366793
H	-0.3425328	2.6646181	-3.8276704
C	1.4816121	4.4965673	-4.6300376
F	1.2942660	4.7303607	-5.9678583
F	0.3847752	4.9847624	-3.9988664
F	2.5384422	5.2678341	-4.2559223
Au	-4.2414646	-5.6493414	1.4607437
Au	-1.3480664	-5.6295414	1.4759437
Au	-2.7837650	-3.1425431	1.4655437
Au	0.0841332	-3.1519431	1.4607437
Au	2.9775314	-3.1321431	1.4759437
Au	1.5418328	-0.6452448	1.4655437
Au	-4.2414646	-0.6546448	1.4607437

Au	-1.3480664	-0.6348448	1.4759437
Au	-2.7837650	1.8521545	1.4655437
Au	0.0841332	1.8427545	1.4607437
Au	2.9775314	1.8625545	1.4759437
Au	1.5418328	-5.6399414	1.4655437
Au	4.4309310	-0.6427448	1.4205438
Au	-5.6840632	-3.1381431	1.4647437
Au	-4.2586646	-4.0497428	3.7494431
Au	-1.3651664	-4.0644428	3.7500431
Au	-2.7712650	-1.5613435	3.8134421
Au	0.0963332	-1.6043435	3.7934431
Au	2.9897314	-1.6190435	3.7941431
Au	1.5837328	0.8839548	3.8574421
Au	-4.1991646	0.9428548	3.8824421
Au	-1.3057664	0.9281548	3.8831421
Au	-2.7117650	3.4312531	3.9464420
Au	0.1558332	3.3882531	3.9265421
Au	3.0492313	3.3735531	3.9271421
Au	1.5242328	-4.1085428	3.7243431
Au	4.4724309	0.8536548	3.7975421
Au	-5.6712632	-1.5227435	3.8278421
Au	2.9338314	3.5196530	-0.7977546
Au	0.0411332	3.4652531	-0.7635546
Au	1.5055328	0.9964547	-0.8466546
Au	-1.3622664	0.9721548	-0.8318546
Au	-4.2550646	0.9178548	-0.7975546
Au	-2.7906650	-1.5508435	-0.8807546

Au	2.9919314	-1.4731436	-0.9244546
Au	0.0991332	-1.5274435	-0.8901546
Au	1.5635328	-3.9962428	-0.9733546
Au	-1.3042664	-4.0206428	-0.9585546
Au	-4.1969646	-4.0749428	-0.9242546
Au	-2.8487650	3.4418531	-0.7541546
Au	-5.6797632	-1.5863435	-0.9062546
Au	4.4055310	1.0263547	-0.8672546

te-m-cn

Te	-0.7198343	-0.1807758	-3.3111912
C	0.9155427	1.1365423	-3.8872873
C	2.2160254	0.6393032	-4.0384578
C	0.6460461	2.4838490	-4.1316229
C	3.2565292	1.5002207	-4.3945549
H	2.4203207	-0.4092524	-3.8370044
C	1.6945566	3.3427545	-4.4975781
C	3.0062044	2.8496144	-4.6188160
H	4.2688355	1.1155204	-4.4728931
H	3.8111651	3.5291095	-4.8761934
H	-0.3545581	2.8839595	-3.9952357
C	1.4265756	4.7308055	-4.7104094
N	1.2054464	5.8581864	-4.8906011
Au	-4.1803123	-5.5147080	1.2854774
Au	-1.2869141	-5.4949080	1.3006774
Au	-2.7226127	-3.0079097	1.2902774
Au	0.1452854	-3.0173097	1.2854774

Au	3.0386836	-2.9975098	1.3006774
Au	1.6029850	-0.5106114	1.2902774
Au	-4.1803123	-0.5200114	1.2854774
Au	-1.2869141	-0.5002115	1.3006774
Au	-2.7226127	1.9867878	1.2902774
Au	0.1452854	1.9773879	1.2854774
Au	3.0386836	1.9971878	1.3006774
Au	1.6029850	-5.5053080	1.2902774
Au	4.4920832	-0.5081114	1.2452774
Au	-5.6229109	-3.0035097	1.2894774
Au	-4.1975123	-3.9151095	3.5741767
Au	-1.3040141	-3.9298095	3.5747767
Au	-2.7101128	-1.4267102	3.6381757
Au	0.1574854	-1.4697102	3.6181767
Au	3.0508836	-1.4844102	3.6188767
Au	1.6448850	1.0185881	3.6821757
Au	-4.1380123	1.0774881	3.7071757
Au	-1.2446142	1.0627881	3.7078757
Au	-2.6506128	3.5658864	3.7711757
Au	0.2169854	3.5228864	3.7512757
Au	3.1103836	3.5081864	3.7518757
Au	1.5853850	-3.9739095	3.5490767
Au	4.5335832	0.9882881	3.6222757
Au	-5.6101109	-1.3881102	3.6525757
Au	2.9949836	3.6542864	-0.9730210
Au	0.1022855	3.5998864	-0.9388210
Au	1.5666850	1.1310881	-1.0219210

Au	-1.3011141	1.1067881	-1.0071210
Au	-4.1939123	1.0524881	-0.9728210
Au	-2.7295127	-1.4162102	-1.0560210
Au	3.0530836	-1.3385102	-1.0997209
Au	0.1602854	-1.3928102	-1.0654210
Au	1.6246850	-3.8616095	-1.1486209
Au	-1.2431142	-3.8860095	-1.1338209
Au	-4.1358123	-3.9403095	-1.0995209
Au	-2.7876127	3.5764864	-0.9294210
Au	-5.6186109	-1.4517102	-1.0815210
Au	4.4666832	1.1609881	-1.0425210

te-m-no2

Te	-0.7987699	-0.2911281	-3.2588765
C	0.8866454	0.9556792	-3.8430370
C	2.1549676	0.4126583	-4.0799049
C	0.6863306	2.3325229	-3.9545300
C	3.2288515	1.2477797	-4.4024773
H	2.3182359	-0.6553876	-3.9608167
C	1.7736476	3.1408486	-4.2688334
C	3.0503274	2.6260877	-4.4926183
H	4.2171408	0.8209627	-4.5430119
H	3.8713457	3.3006845	-4.6998456
H	-0.2769580	2.7866910	-3.7486745
N	1.5839269	4.6023296	-4.2543618
O	0.5048614	5.0344255	-3.8288552
O	2.5245058	5.3117961	-4.6306181

Au	-4.2213083	-5.5716883	1.3357185
Au	-1.3279101	-5.5518883	1.3509185
Au	-2.7636087	-3.0648900	1.3405185
Au	0.1042895	-3.0742900	1.3357185
Au	2.9976877	-3.0544900	1.3509185
Au	1.5619891	-0.5675917	1.3405185
Au	-4.2213083	-0.5769917	1.3357185
Au	-1.3279101	-0.5571917	1.3509185
Au	-2.7636087	1.9298076	1.3405185
Au	0.1042895	1.9204076	1.3357185
Au	2.9976877	1.9402076	1.3509185
Au	1.5619891	-5.5622883	1.3405185
Au	4.4510872	-0.5650917	1.2955185
Au	-5.6639069	-3.0604900	1.3397185
Au	-4.2385083	-3.9720898	3.6244179
Au	-1.3450101	-3.9867898	3.6250179
Au	-2.7511087	-1.4836905	3.6884169
Au	0.1164895	-1.5266904	3.6684179
Au	3.0098876	-1.5413904	3.6691179
Au	1.6038890	0.9616079	3.7324169
Au	-4.1790083	1.0205078	3.7574169
Au	-1.2856101	1.0058078	3.7581169
Au	-2.6916088	3.5089061	3.8214168
Au	0.1759894	3.4659062	3.8015168
Au	3.0693876	3.4512062	3.8021168
Au	1.5443891	-4.0308897	3.5993179
Au	4.4925872	0.9313079	3.6725169

Au	-5.6511069	-1.4450905	3.7028169
Au	2.9539877	3.5973061	-0.9227798
Au	0.0612895	3.5429061	-0.8885798
Au	1.5256891	1.0741078	-0.9716798
Au	-1.3421101	1.0498078	-0.9568798
Au	-4.2349083	0.9955079	-0.9225798
Au	-2.7705087	-1.4731905	-1.0057798
Au	3.0120876	-1.3954905	-1.0494798
Au	0.1192895	-1.4497905	-1.0151798
Au	1.5836890	-3.9185898	-1.0983798
Au	-1.2841101	-3.9429898	-1.0835798
Au	-4.1768083	-3.9972897	-1.0492798
Au	-2.8286087	3.5195061	-0.8791798
Au	-5.6596069	-1.5086904	-1.0312798
Au	4.4256872	1.1040078	-0.9922798

ORTHO

te-o-nh2

Te	-0.6609614	-0.0251935	-3.3021096
C	1.1969990	0.9689939	-3.8133274
C	2.3544842	0.1842040	-3.9320103
C	1.2594892	2.3629472	-4.0577629
C	3.5902995	0.7603927	-4.2138728

H	2.2803405	-0.8892194	-3.7707476
C	2.5238584	2.9316937	-4.3177446
C	3.6667973	2.1484417	-4.3850671
H	4.4775980	0.1392851	-4.2793283
H	2.5943845	4.0098556	-4.4419860
N	0.1260788	3.1553778	-4.0576603
H	0.2870799	4.1434571	-3.8901940
H	-0.6559642	2.8048724	-3.5041765
H	4.6271866	2.6227126	-4.5640800
Au	-4.2787541	-5.4158939	1.3292557
Au	-1.3853560	-5.3960939	1.3444557
Au	-2.8210546	-2.9090956	1.3340557
Au	0.0468436	-2.9184956	1.3292557
Au	2.9402418	-2.8986956	1.3444557
Au	1.5045432	-0.4117973	1.3340557
Au	-4.2787541	-0.4211973	1.3292557
Au	-1.3853560	-0.4013973	1.3444557
Au	-2.8210546	2.0856020	1.3340557
Au	0.0468436	2.0762020	1.3292557
Au	2.9402418	2.0960020	1.3444557
Au	1.5045432	-5.4064939	1.3340557
Au	4.3936414	-0.4092973	1.2890557
Au	-5.7213527	-2.9046956	1.3332557
Au	-4.2959541	-3.8162954	3.6179551
Au	-1.4024560	-3.8309954	3.6185551
Au	-2.8085546	-1.3278961	3.6819541
Au	0.0590436	-1.3708961	3.6619551

Au	2.9524418	-1.3855961	3.6626551
Au	1.5464432	1.1174022	3.7259540
Au	-4.2364542	1.1763022	3.7509540
Au	-1.3430560	1.1616022	3.7516540
Au	-2.7490546	3.6647005	3.8149540
Au	0.1185436	3.6217005	3.7950540
Au	3.0119418	3.6070005	3.7956540
Au	1.4869432	-3.8750954	3.5928551
Au	4.4351414	1.0871022	3.6660541
Au	-5.7085527	-1.2892961	3.6963541
Au	2.8965418	3.7531005	-0.9292426
Au	0.0038436	3.6987005	-0.8950427
Au	1.4682432	1.2299022	-0.9781426
Au	-1.3995560	1.2056022	-0.9633426
Au	-4.2923541	1.1513022	-0.9290426
Au	-2.8279546	-1.3173961	-1.0122426
Au	2.9546418	-1.2396961	-1.0559426
Au	0.0618436	-1.2939961	-1.0216426
Au	1.5262432	-3.7627954	-1.1048426
Au	-1.3415560	-3.7871954	-1.0900426
Au	-4.2342542	-3.8414954	-1.0557426
Au	-2.8860545	3.6753005	-0.8856427
Au	-5.7170527	-1.3528961	-1.0377426
Au	4.3682414	1.2598022	-0.9987426

te-o-och3

Te	-0.6074775	-0.3661429	-3.0900166

C	1.0757474	0.8712970	-3.6670349
C	2.3792785	0.3742758	-3.7328045
C	0.8300982	2.2205845	-3.9851870
C	3.4481651	1.2182516	-4.0470926
H	2.5624196	-0.6717591	-3.4976119
C	1.9009959	3.0709282	-4.2799180
C	3.2024581	2.5660414	-4.2994152
H	4.4595840	0.8250270	-4.0651329
H	1.7284437	4.1240602	-4.4733228
Au	-4.1198351	-5.7015636	1.5126684
Au	-1.2264369	-5.6817636	1.5278684
Au	-2.6621355	-3.1947653	1.5174684
Au	0.2057627	-3.2041653	1.5126684
Au	3.0991609	-3.1843653	1.5278684
Au	1.6634623	-0.6974670	1.5174684
Au	-4.1198351	-0.7068670	1.5126684
Au	-1.2264369	-0.6870670	1.5278684
Au	-2.6621355	1.7999323	1.5174684
Au	0.2057627	1.7905323	1.5126684
Au	3.0991609	1.8103323	1.5278684
Au	1.6634623	-5.6921636	1.5174684
Au	4.5525605	-0.6949670	1.4724684
Au	-5.5624337	-3.1903653	1.5166684
Au	-4.1370351	-4.1019651	3.8013677
Au	-1.2435369	-4.1166651	3.8019677
Au	-2.6496355	-1.6135658	3.8653667
Au	0.2179627	-1.6565658	3.8453677

Au	3.1113609	-1.6712658	3.8460677
Au	1.7053623	0.8317325	3.9093667
Au	-4.0775351	0.8906325	3.9343667
Au	-1.1841369	0.8759325	3.9350667
Au	-2.5901355	3.3790308	3.9983667
Au	0.2774627	3.3360308	3.9784667
Au	3.1708609	3.3213308	3.9790667
Au	1.6458623	-4.1607651	3.7762677
Au	4.5940605	0.8014325	3.8494667
Au	-5.5496337	-1.5749658	3.8797667
Au	3.0554609	3.4674308	-0.7458300
Au	0.1627627	3.4130308	-0.7116300
Au	1.6271623	0.9442325	-0.7947300
Au	-1.2406369	0.9199325	-0.7799300
Au	-4.1334351	0.8656325	-0.7456300
Au	-2.6690355	-1.6030658	-0.8288300
Au	3.1135609	-1.5253658	-0.8725300
Au	0.2207627	-1.5796658	-0.8382300
Au	1.6851623	-4.0484651	-0.9214299
Au	-1.1826369	-4.0728651	-0.9066300
Au	-4.0753351	-4.1271651	-0.8723300
Au	-2.7271355	3.3896308	-0.7022300
Au	-5.5581337	-1.6385658	-0.8543300
Au	4.5271605	0.9741325	-0.8153300
C	-0.7746309	4.0032153	-4.0938697
O	-0.4759214	2.6094474	-3.9519630
H	-1.8495067	4.0817989	-3.9278230

H	-0.5160422	4.3578817	-5.1000559
H	-0.2432161	4.5910077	-3.3341607
H	4.0276621	3.2399771	-4.5090377

te-o-ch3

Te	-0.6516846	-0.2616626	-3.1528343
C	0.9570799	1.0717512	-3.7679469
C	2.2752115	0.5973219	-3.7392311
C	0.6761318	2.3898733	-4.1775726
C	3.3392932	1.4447832	-4.0516554
H	2.4711492	-0.4339670	-3.4548415
C	1.7650554	3.2256000	-4.4590476
C	3.0812257	2.7720821	-4.3876845
H	4.3574176	1.0687709	-4.0042533
H	1.5677300	4.2609021	-4.7280961
Au	-4.0939024	-5.5715335	1.4551112
Au	-1.2005042	-5.5517335	1.4703111
Au	-2.6362028	-3.0647352	1.4599112
Au	0.2316954	-3.0741352	1.4551112
Au	3.1250936	-3.0543352	1.4703111
Au	1.6893950	-0.5674369	1.4599112
Au	-4.0939024	-0.5768369	1.4551112
Au	-1.2005042	-0.5570369	1.4703111
Au	-2.6362028	1.9299624	1.4599112
Au	0.2316954	1.9205624	1.4551112
Au	3.1250936	1.9403624	1.4703111
Au	1.6893950	-5.5621335	1.4599112

Au	4.5784932	-0.5649369	1.4149112
Au	-5.5365010	-3.0603352	1.4591112
Au	-4.1111024	-3.9719350	3.7438105
Au	-1.2176042	-3.9866350	3.7444105
Au	-2.6237028	-1.4835357	3.8078095
Au	0.2438954	-1.5265357	3.7878105
Au	3.1372936	-1.5412357	3.7885105
Au	1.7312950	0.9617626	3.8518095
Au	-4.0516024	1.0206626	3.8768095
Au	-1.1582042	1.0059626	3.8775095
Au	-2.5642028	3.5090609	3.9408095
Au	0.3033954	3.4660609	3.9209095
Au	3.1967935	3.4513609	3.9215095
Au	1.6717950	-4.0307350	3.7187105
Au	4.6199931	0.9314627	3.7919095
Au	-5.5237010	-1.4449357	3.8222095
Au	3.0813936	3.5974609	-0.8033872
Au	0.1886954	3.5430609	-0.7691872
Au	1.6530950	1.0742626	-0.8522872
Au	-1.2147042	1.0499626	-0.8374872
Au	-4.1075024	0.9956626	-0.8031872
Au	-2.6431028	-1.4730357	-0.8863872
Au	3.1394936	-1.3953357	-0.9300872
Au	0.2466954	-1.4496357	-0.8957872
Au	1.7110950	-3.9184350	-0.9789872
Au	-1.1567042	-3.9428350	-0.9641872
Au	-4.0494024	-3.9971350	-0.9298872

Au	-2.7012028	3.5196609	-0.7597872
Au	-5.5322010	-1.5085357	-0.9118872
Au	4.5530932	1.1041626	-0.8728872
H	3.9010817	3.4540840	-4.5929175
C	-0.7284139	2.9207979	-4.2757970
H	-1.3352194	2.3230711	-4.9664763
H	-1.2259348	2.8817292	-3.2965277
H	-0.7303360	3.9591584	-4.6187954

te-o-f

Te	-0.6662035	-0.0296976	-3.3993610
C	1.0160267	1.2013745	-4.0031718
C	2.3256336	0.7027487	-4.0639478
C	0.8182673	2.5386383	-4.3434523
C	3.3930977	1.5372843	-4.3951820
H	2.5080883	-0.3389167	-3.8113523
C	1.8662247	3.3938677	-4.6544524
C	3.1645437	2.8863977	-4.6707161
H	4.4017378	1.1360912	-4.4079235
H	1.6542315	4.4384337	-4.8582701
Au	-4.1888971	-5.3905570	1.2114595
Au	-1.2954989	-5.3707570	1.2266595
Au	-2.7311975	-2.8837587	1.2162595
Au	0.1367007	-2.8931587	1.2114595
Au	3.0300989	-2.8733587	1.2266595
Au	1.5944003	-0.3864604	1.2162595
Au	-4.1888971	-0.3958604	1.2114595

Au	-1.2954989	-0.3760604	1.2266595
Au	-2.7311975	2.1109389	1.2162595
Au	0.1367007	2.1015389	1.2114595
Au	3.0300989	2.1213389	1.2266595
Au	1.5944003	-5.3811570	1.2162595
Au	4.4834984	-0.3839604	1.1712595
Au	-5.6314957	-2.8793587	1.2154595
Au	-4.2060971	-3.7909585	3.5001589
Au	-1.3125989	-3.8056584	3.5007589
Au	-2.7186975	-1.3025592	3.5641579
Au	0.1489007	-1.3455591	3.5441589
Au	3.0422988	-1.3602591	3.5448589
Au	1.6363002	1.1427392	3.6081578
Au	-4.1465971	1.2016391	3.6331578
Au	-1.2531989	1.1869391	3.6338578
Au	-2.6591975	3.6900374	3.6971578
Au	0.2084006	3.6470375	3.6772578
Au	3.1017988	3.6323375	3.6778578
Au	1.5768003	-3.8497584	3.4750589
Au	4.5249984	1.1124392	3.5482579
Au	-5.6186957	-1.2639592	3.5785578
Au	2.9863989	3.7784374	-1.0470389
Au	0.0937007	3.7240374	-1.0128389
Au	1.5581003	1.2552391	-1.0959388
Au	-1.3096989	1.2309391	-1.0811388
Au	-4.2024971	1.1766392	-1.0468389
Au	-2.7380975	-1.2920592	-1.1300388

Au 3.0444988 -1.2143592 -1.1737388

Au 0.1517007 -1.2686592 -1.1394388

Au 1.6161002 -3.7374585 -1.2226388

Au -1.2516989 -3.7618585 -1.2078388

Au -4.1443971 -3.8161584 -1.1735388

Au -2.7961975 3.7006374 -1.0034389

Au -5.6271957 -1.3275591 -1.1555388

Au 4.4580985 1.2851391 -1.1165388

F -0.4413138 3.0472591 -4.3387499

H 3.9959348 3.5484642 -4.8915353

te-o-cl

Te -0.7000706 -0.0746785 -3.3930079

C 0.9337173 1.2278821 -3.9994506

C 2.2486580 0.7368542 -4.0405340

C 0.7201395 2.5690354 -4.3423062

C 3.3183256 1.5739703 -4.3524004

H 2.4311557 -0.3056136 -3.7915257

C 1.7877962 3.4228060 -4.6228768

C 3.0879612 2.9238868 -4.6203070

H 4.3282258 1.1749731 -4.3517899

H 1.5915853 4.4707715 -4.8236258

H 3.9186018 3.5935111 -4.8206419

Cl -0.8927834 3.2412620 -4.3663254

Au -4.1622777 -5.3976884 1.2100377

Au -1.2688795 -5.3778884 1.2252377

Au -2.7045781 -2.8908901 1.2148377

Au	0.1633201	-2.9002901	1.2100377
Au	3.0567183	-2.8804901	1.2252377
Au	1.6210197	-0.3935918	1.2148377
Au	-4.1622777	-0.4029918	1.2100377
Au	-1.2688795	-0.3831918	1.2252377
Au	-2.7045781	2.1038075	1.2148377
Au	0.1633201	2.0944075	1.2100377
Au	3.0567183	2.1142075	1.2252377
Au	1.6210197	-5.3882884	1.2148377
Au	4.5101179	-0.3910918	1.1698377
Au	-5.6048763	-2.8864901	1.2140377
Au	-4.1794777	-3.7980899	3.4987370
Au	-1.2859795	-3.8127899	3.4993370
Au	-2.6920781	-1.3096906	3.5627360
Au	0.1755201	-1.3526906	3.5427370
Au	3.0689183	-1.3673906	3.5434370
Au	1.6629197	1.1356077	3.6067360
Au	-4.1199777	1.1945077	3.6317360
Au	-1.2265795	1.1798077	3.6324360
Au	-2.6325781	3.6829060	3.6957360
Au	0.2350201	3.6399060	3.6758360
Au	3.1284183	3.6252060	3.6764360
Au	1.6034197	-3.8568899	3.4736370
Au	4.5516179	1.1053077	3.5468360
Au	-5.5920763	-1.2710906	3.5771360
Au	3.0130183	3.7713060	-1.0484607
Au	0.1203201	3.7169060	-1.0142607

Au	1.5847197	1.2481077	-1.0973607
Au	-1.2830795	1.2238077	-1.0825607
Au	-4.1758777	1.1695077	-1.0482607
Au	-2.7114781	-1.2991906	-1.1314607
Au	3.0711183	-1.2214906	-1.1751607
Au	0.1783201	-1.2757906	-1.1408607
Au	1.6427197	-3.7445899	-1.2240606
Au	-1.2250795	-3.7689899	-1.2092607
Au	-4.1177777	-3.8232899	-1.1749607
Au	-2.7695781	3.6935060	-1.0048607
Au	-5.6005763	-1.3346906	-1.1569607
Au	4.4847179	1.2780077	-1.1179607

te-o-ococh3

Te	-0.8059256	0.2250665	-2.8400909
C	0.9419821	1.3675752	-3.4342084
C	2.2026680	0.7772391	-3.6009858
C	0.8234154	2.7458248	-3.6656590
C	3.3252593	1.5374925	-3.9215717
C	1.9358049	3.5104190	-4.0045464
H	-0.1404609	3.2252404	-3.5150823
C	3.1905134	2.9092636	-4.1231460
H	4.2918983	1.0463502	-3.9737053
H	1.8278505	4.5825752	-4.1377298
H	4.0670546	3.5083368	-4.3489479
C	1.6035084	-1.2853810	-5.4560416
H	2.3524833	-0.6714985	-5.9665499

C	2.0752530	-1.6328182	-4.0680576
H	0.6784172	-0.7016042	-5.4064297
H	1.4267922	-2.2102972	-6.0037212
O	2.2099665	-2.7436997	-3.6226508
O	2.4225546	-0.5636075	-3.2544387
Au	-4.4397399	-5.1851527	1.7772143
Au	-1.5463407	-5.1653527	1.7924143
Au	-2.9820403	-2.6783544	1.7820143
Au	-0.1141421	-2.6877544	1.7772143
Au	2.7792561	-2.6679544	1.7924143
Au	1.3435575	-0.1810561	1.7820143
Au	-4.4397399	-0.1904561	1.7772143
Au	-1.5463407	-0.1706561	1.7924143
Au	-2.9820403	2.3163432	1.7820143
Au	-0.1141421	2.3069432	1.7772143
Au	2.7792561	2.3267432	1.7924143
Au	1.3435575	-5.1757527	1.7820143
Au	4.2326557	-0.1785561	1.7370143
Au	-5.8823385	-2.6739544	1.7812143
Au	-4.4569399	-3.5855542	4.0659126
Au	-1.5634407	-3.6002542	4.0665126
Au	-2.9695403	-1.0971549	4.1299126
Au	-0.1019421	-1.1401549	4.1099126
Au	2.7914561	-1.1548549	4.1106126
Au	1.3854575	1.3481434	4.1739126
Au	-4.3974399	1.4070434	4.1989126
Au	-1.5040407	1.3923434	4.1996126

Au	-2.9100403	3.8954417	4.2629126
Au	-0.0424421	3.8524417	4.2430126
Au	2.8509561	3.8377417	4.2436126
Au	1.3259575	-3.6443542	4.0408126
Au	4.2741557	1.3178434	4.1140126
Au	-5.8695385	-1.0585549	4.1443126
Au	2.7355561	3.9838417	-0.4812851
Au	-0.1571421	3.9294417	-0.4470851
Au	1.3072575	1.4606434	-0.5301851
Au	-1.5605407	1.4363434	-0.5153851
Au	-4.4533399	1.3820434	-0.4810851
Au	-2.9889403	-1.0866549	-0.5642851
Au	2.7936561	-1.0089549	-0.6079851
Au	-0.0991421	-1.0632549	-0.5736851
Au	1.3652575	-3.5320542	-0.6568850
Au	-1.5025407	-3.5564542	-0.6420850
Au	-4.3952399	-3.6107542	-0.6077851
Au	-3.0470402	3.9060417	-0.4376851
Au	-5.8780385	-1.1221549	-0.5897851
Au	4.2072557	1.4905434	-0.5507851

te-o-cf3

Te	-0.6700756	0.1473238	-3.1759782
C	0.7058127	1.7166593	-3.8572368
C	2.0402225	1.5751126	-4.2726244
C	0.1493997	3.0050368	-3.8320250
C	2.8123068	2.7129131	-4.5466640

C	0.9108623	4.1275689	-4.1405891
C	2.2605849	3.9855658	-4.4623885
H	3.8550599	2.5865502	-4.8192197
H	0.4553493	5.1124698	-4.0890595
H	2.8772019	4.8571515	-4.6575244
H	-0.8867659	3.1284254	-3.5283025
C	2.7132574	0.2540609	-4.5547909
F	2.7013005	-0.0056379	-5.9027793
F	4.0235494	0.2573190	-4.1862178
F	2.1418519	-0.8172879	-3.9602740
Au	-4.2350245	-5.2851668	1.4398171
Au	-1.3416263	-5.2653668	1.4550171
Au	-2.7773249	-2.7783685	1.4446171
Au	0.0905733	-2.7877685	1.4398171
Au	2.9839715	-2.7679685	1.4550171
Au	1.5482729	-0.2810702	1.4446171
Au	-4.2350245	-0.2904702	1.4398171
Au	-1.3416263	-0.2706702	1.4550171
Au	-2.7773249	2.2163291	1.4446171
Au	0.0905733	2.2069291	1.4398171
Au	2.9839715	2.2267291	1.4550171
Au	1.5482729	-5.2757668	1.4446171
Au	4.4373711	-0.2785702	1.3996171
Au	-5.6776231	-2.7739685	1.4438171
Au	-4.2522245	-3.6855683	3.7285165
Au	-1.3587263	-3.7002683	3.7291165
Au	-2.7648249	-1.1971690	3.7925154

Au	0.1027733	-1.2401690	3.7725164
Au	2.9961715	-1.2548689	3.7732164
Au	1.5901729	1.2481293	3.8365154
Au	-4.1927245	1.3070293	3.8615154
Au	-1.2993263	1.2923293	3.8622154
Au	-2.7053249	3.7954276	3.9255154
Au	0.1622733	3.7524276	3.9056154
Au	3.0556714	3.7377277	3.9062154
Au	1.5306729	-3.7443682	3.7034165
Au	4.4788710	1.2178294	3.7766154
Au	-5.6648231	-1.1585690	3.8069154
Au	2.9402715	3.8838276	-0.8186813
Au	0.0475733	3.8294276	-0.7844813
Au	1.5119729	1.3606293	-0.8675813
Au	-1.3558263	1.3363293	-0.8527813
Au	-4.2486245	1.2820293	-0.8184813
Au	-2.7842249	-1.1866690	-0.9016812
Au	2.9983715	-1.1089690	-0.9453812
Au	0.1055733	-1.1632690	-0.9110812
Au	1.5699729	-3.6320683	-0.9942812
Au	-1.2978263	-3.6564683	-0.9794812
Au	-4.1905245	-3.7107683	-0.9451812
Au	-2.8423249	3.8060276	-0.7750813
Au	-5.6733231	-1.2221690	-0.9271812
Au	4.4119711	1.3905293	-0.8881813

te-o-cn

Te	-0.7504255	-0.1761354	-3.3014713
C	0.7813301	1.2504585	-3.9166509
C	2.1247473	0.8644158	-3.9695355
C	0.4319051	2.5769072	-4.2477510
C	3.1150391	1.7904389	-4.3001999
H	2.3965719	-0.1579895	-3.7203396
C	1.4384965	3.5060165	-4.5715977
C	2.7717384	3.1134127	-4.5865809
H	4.1559436	1.4808689	-4.3002778
H	1.1592029	4.5339000	-4.7801728
H	3.5447166	3.8421074	-4.8091920
C	-0.9232592	3.0255097	-4.1881476
N	-2.0115659	3.4353280	-4.1311904
Au	-4.0746253	-5.4305974	1.2874811
Au	-1.1812271	-5.4107974	1.3026811
Au	-2.6169257	-2.9237991	1.2922811
Au	0.2509725	-2.9331991	1.2874811
Au	3.1443707	-2.9133991	1.3026811
Au	1.7086721	-0.4265008	1.2922811
Au	-4.0746253	-0.4359008	1.2874811
Au	-1.1812271	-0.4161008	1.3026811
Au	-2.6169257	2.0708985	1.2922811
Au	0.2509725	2.0614985	1.2874811
Au	3.1443707	2.0812985	1.3026811
Au	1.7086721	-5.4211974	1.2922811
Au	4.5977703	-0.4240008	1.2472811
Au	-5.5172239	-2.9193991	1.2914811

Au	-4.0918253	-3.8309988	3.5761805
Au	-1.1983271	-3.8456988	3.5767805
Au	-2.6044257	-1.3425995	3.6401795
Au	0.2631725	-1.3855995	3.6201805
Au	3.1565707	-1.4002995	3.6208805
Au	1.7505721	1.1026988	3.6841795
Au	-4.0323253	1.1615988	3.7091795
Au	-1.1389271	1.1468988	3.7098795
Au	-2.5449257	3.6499971	3.7731794
Au	0.3226725	3.6069971	3.7532794
Au	3.2160707	3.5922971	3.7538794
Au	1.6910721	-3.8897988	3.5510805
Au	4.6392703	1.0723988	3.6242795
Au	-5.5044239	-1.3039995	3.6545795
Au	3.1006707	3.7383971	-0.9710172
Au	0.2079725	3.6839971	-0.9368172
Au	1.6723721	1.2151988	-1.0199172
Au	-1.1954271	1.1908988	-1.0051172
Au	-4.0882253	1.1365988	-0.9708172
Au	-2.6238257	-1.3320995	-1.0540172
Au	3.1587707	-1.2543995	-1.0977172
Au	0.2659725	-1.3086995	-1.0634172
Au	1.7303721	-3.7774988	-1.1466172
Au	-1.1374271	-3.8018988	-1.1318172
Au	-4.0301253	-3.8561988	-1.0975172
Au	-2.6819256	3.6605971	-0.9274172
Au	-5.5129239	-1.3675995	-1.0795172

Au	4.5723703	1.2450988	-1.0405172

te-o-no2

Te	-0.7193573	-0.2359609	-3.2285010
C	0.9186041	1.0628762	-3.8679831
C	2.2083376	0.5164745	-3.9619919
C	0.7883424	2.4424555	-4.0877592
C	3.3112773	1.3186472	-4.2469068
H	2.3478990	-0.5447074	-3.7716903
C	1.8969005	3.2693528	-4.2949929
C	3.1596419	2.7010752	-4.3917754
H	4.2966638	0.8660621	-4.3068436
H	1.7418096	4.3394184	-4.3693840
H	4.0254522	3.3336297	-4.5573677
N	-0.5123093	3.0839380	-3.9699759
O	-1.5314141	2.3916578	-4.1438092
O	-0.5402442	4.2732021	-3.5912057
Au	-4.0669008	-5.4827581	1.3490792
Au	-1.1735026	-5.4629581	1.3642792
Au	-2.6092012	-2.9759598	1.3538792
Au	0.2586970	-2.9853598	1.3490792
Au	3.1520952	-2.9655598	1.3642792
Au	1.7163966	-0.4786615	1.3538792
Au	-4.0669008	-0.4880615	1.3490792
Au	-1.1735026	-0.4682615	1.3642792
Au	-2.6092012	2.0187378	1.3538792
Au	0.2586970	2.0093378	1.3490792

Au	3.1520952	2.0291378	1.3642792
Au	1.7163966	-5.4733581	1.3538792
Au	4.6054948	-0.4761615	1.3088792
Au	-5.5094994	-2.9715598	1.3530792
Au	-4.0841008	-3.8831595	3.6377786
Au	-1.1906026	-3.8978595	3.6383786
Au	-2.5967012	-1.3947602	3.7017776
Au	0.2708970	-1.4377602	3.6817786
Au	3.1642952	-1.4524602	3.6824786
Au	1.7582966	1.0505381	3.7457775
Au	-4.0246008	1.1094381	3.7707775
Au	-1.1312026	1.0947381	3.7714775
Au	-2.5372012	3.5978364	3.8347775
Au	0.3303970	3.5548364	3.8148775
Au	3.2237952	3.5401364	3.8154775
Au	1.6987966	-3.9419595	3.6126786
Au	4.6469948	1.0202381	3.6858776
Au	-5.4966994	-1.3561602	3.7161776
Au	3.1083952	3.6862364	-0.9094191
Au	0.2156970	3.6318364	-0.8752192
Au	1.6800966	1.1630381	-0.9583191
Au	-1.1877026	1.1387381	-0.9435191
Au	-4.0805008	1.0844381	-0.9092191
Au	-2.6161012	-1.3842602	-0.9924191
Au	3.1664952	-1.3065602	-1.0361191
Au	0.2736970	-1.3608602	-1.0018191
Au	1.7380966	-3.8296595	-1.0850191

Au	-1.1297026	-3.8540595	-1.0702191
Au	-4.0224008	-3.9083595	-1.0359191
Au	-2.6742011	3.6084364	-0.8658192
Au	-5.5051994	-1.4197602	-1.0179191
Au	4.5800948	1.1929381	-0.9789191
